# A systematic review and meta-analysis on the prevalence and impact of coronary artery disease in hospitalized COVID-19 patients

**DOI:** 10.1016/j.heliyon.2023.e19493

**Published:** 2023-08-25

**Authors:** Mohammed A. Merzah, Dahy Sulaiman, Atiya Abul Karim, Mazin E. Khalil, Sabyasachi Gupta, Yasir Almuzaini, Shima Hashemi, Stany Mathew, Salina Khatoon, Mohima Benojir Hoque

**Affiliations:** a Department of Public Health and Epidemiology, Faculty of General Medicine, University of Debrecen, Debrecen, Hungary; b Health Technology Assessment Resource Centre, Department of Public Health, Kalyan Singh Super Specialty Cancer Institute, Lucknow, India; c Solutions 4 Health, UK; d School of Medicine, St. George's University, West Indies, Grenada; e Government of Canada, Canada; f Global Center of Mass Gatherings Medicine, Ministry of Health, Saudi Arabia; g Department of Epidemiology, Faculty of Health, Ilam University of Medical Sciences, Ilam, Iran; h Health Technology Assessment Resource Centre, National Centre for Disease Informatics and Research, Bangalore, India; i University of Sheffield, Sheffield, UK; j Department of Public Health, ASA University Bangladesh, Bangladesh

**Keywords:** Coronary artery disease, CAD, COVID-19, ARDS, Mortality, Prevalence

## Abstract

**Background:**

COVID-19 accounts for more than half a billion deaths globally. The clinical manifestations may vary in due course. Despite several studies aimed at determining the extent to which the disease's severity and mortality remain high when combined with other comorbidities, more research is required. Therefore, this review aimed to measure the pooled prevalence of coronary artery disease (CAD) among COVID-19 patients, specifically those with a history of CAD. Additionally, we aim to assess the association between mortality due to CAD and the severity of COVID-19 among hospitalized patients.

**Method:**

A comprehensive search in PubMed, Web of Science, the Cochrane Library, and the WHO COVID-19 database was conducted. English studies with original data on CAD, mortality, and ARDS among COVID-19 patients were included. PRISMA guidelines were followed.

**Results:**

Among the 2007 identified articles, 76 studies met the inclusion criteria. The pooled prevalence of CAD among COVID-19 patients was 14.4%(95% CI: 12.7–16.2). The highest prevalence was observed in European studies at 18.2%(95% CI: 13.3–24.2), while the lowest was in Asian studies at 10.4% (95% CI: 6.4–16.3). Participants with concurrent CAD at the time of hospital admission had twice the odds of mortality due to COVID-19 (2.64 [95% CI: 2.30–3.04]) with moderate heterogeneity (I2 = 45%, p < 0.01). Hospitalized COVID-19 patients with CAD had a 50% higher risk of ARDS (95% CI: 0.62–3.66), but this difference was not statistically significant.

**Conclusion:**

Although our analysis revealed evidence for a relationship between concurrent CAD at the time of hospital admission and mortality from COVID-19, however, global variation in health infrastructure, limitations of data reporting, and the effects of emerging variants must be considered in future investigations.

## 1 Introduction

The coronavirus disease 2019 (COVID-19) has posed a major threat to healthcare systems worldwide [1]. A total of 651,918,402 people have been infected and 6,656,601 have died due to COVID-19 up to December 22, 2022 [2].

The clinical manifestations of COVID-19 are varied [3]. The condition may proceed to Acute Respiratory Distress Syndrome (ARDS) in some patients, whereas it may be asymptomatic in others [4]. ARDS is a condition characterized by damage to the epithelial and endothelial barriers of the alveoli [5]. The incidence of ARDS is 15–18% of all coronary artery disease (CAD) patients and is associated with a mortality rate of 50% [6]. While the incidence of ARDS among COVID-19 patients was reported to be 33% [7]. An average of 25–50% of COVID-19 deaths appear to be related to the presence of severe ARDS [8].

CAD causes almost 7 million deaths and 129 million disability-adjusted life years (DALYs) lost annually, making it the leading cause of mortality and DALYs loss worldwide [9]. A high proportion of COVID-19 patients have comorbidities [10,11]. Several studies suggest that patients admitted to hospitals with severe COVID-19 have a relatively high prevalence of CAD, and CAD may be associated with a significant risk of mortality due to COVID-19 [[10], [11], [12], [13]]. A meta-analysis concludes that patients who have preexisting CAD and contract COVID-19 face an increased risk of mortality [14].Since CAD causes insufficient oxygen supply to the heart, an infection of the respiratory system may reduce oxygenation capacity, aggravating the heart's oxygen shortage. Recent studies suggest that CAD might lead to severe COVID-19 due to exacerbating hypoxemia [15,16]. Therefore, this review is essential to quantify evidence on the pooled prevalence of CAD among patients suffering from COVID-19, including those with a history of CAD. We further intend to measure the strength of evidence for an association between CAD and COVID-19 severity as measured by ARDS rate and in-hospital mortality.

## 2 Methodology

### 2.1 Literature search

The initial literature search was conducted using PubMed, where terms such as “COVID-19”, “Prevalence”, “Mortality”, and “Coronary Artery Disease” were used with Boolean operators to retrieve the first few articles. A subsequent search using all identified keywords and index terms was conducted up to 29th April 2022 in PubMed, Web of Science, Cochrane Central Register of Controlled Trials, and the World Health Organization (WHO) COVID-19 global literature on coronavirus disease (Detailed search strategy in supplementary appendix, Appendix A_ Tables A.1-A.5). Search results from electronic databases were exported into Rayyan software [17] for handling the articles. The protocol of this review was registered with the International Prospective Register of Systematic Reviews (PROSPERO), having a registration number (CRD42022308188), and the article followed PRISMA guidelines.

### 2.2 Inclusion and exclusion criteria

The study was restricted to hospitalized adult patients who were 18 years or above with a confirmed COVID-19. We confined our review to studies that had reported the prevalence of CAD with a comparison group i.e., patients with and without CAD. The search strategy excluded all non-human studies and all articles that were not written in English. Articles excluded from our search strategy included case reports, case series, letters, systematic reviews, meta-analyses, and those that lacked full texts.

### 2.3 Data extraction and synthesis

Two authors independently extracted the data from each study into an Excel database, while another two authors arbitrated when discrepancies occurred. Data on the study design, study period, study population, and demographics (sample size, age, gender, and confirmed diagnosis of COVID-19 infection) were extracted from articles meeting inclusion criteria. In addition, we retrieved effect estimates on the association between the severity of COVID-19 and CAD, ARDS, and mortality caused by these conditions.

### 2.4 Quality assessment of included studies

The Newcastle-Ottawa quality assessment scale (NOS) [18] was used to assess the methodological quality and risk of bias of all included articles. The tool has eight items with a minimum score of zero and a maximum score of nine. The overall risk of bias was defined as low, if the cumulative score on eight items for the respective study ranged between 7 and 9, moderate for a score of 4–6, and the study with a score of 0–3 was classified to have a high risk of bias. To maintain reliability, two reviewers were calibrated on 8 items of quality assessment tools and further assessed the quality of included studies independently. The supplementary appendix contains a summary of the risk of bias for all included articles (Appendix B).

### 2.5 Statistical analysis

A meta-analysis was performed in RStudio.Version (3.3.0) [19] for all eligible studies (n = 76). For the primary outcome of interest, the proportions of CAD in the study population for respective studies were pooled together and the precise summary estimates were reported as prevalence with 95% Confidence Intervals (CIs). We had pre-specified to use a random-effect model when I^2^ was higher than 50% [20]. We further evaluated statistical heterogeneity by using the I-squared test and defined considerable heterogeneity at an I^2^ value greater than 75%, moderate heterogeneity for a value between 25 and 27%, and for an I^2^ value of less than 25% to categorize a low degree of heterogeneity [21]. For studies that reported similar outcomes of interest, we reported pooled effect size as Odds Ratios (ORs), the degree of uncertainty as 95% CIs, and the strength of evidence for hypothesized associations in the forest plot with weights given to individual studies based on their sample size. We further conducted a pre-specified subgroup analysis to control for heterogeneity in pooled estimates under the assumption that any variations in the sampling of study participants in individual studies might have been caused by geographical and health system variation.

## 3 Results

### 3.1 Results of the search

**Fig. 1:**
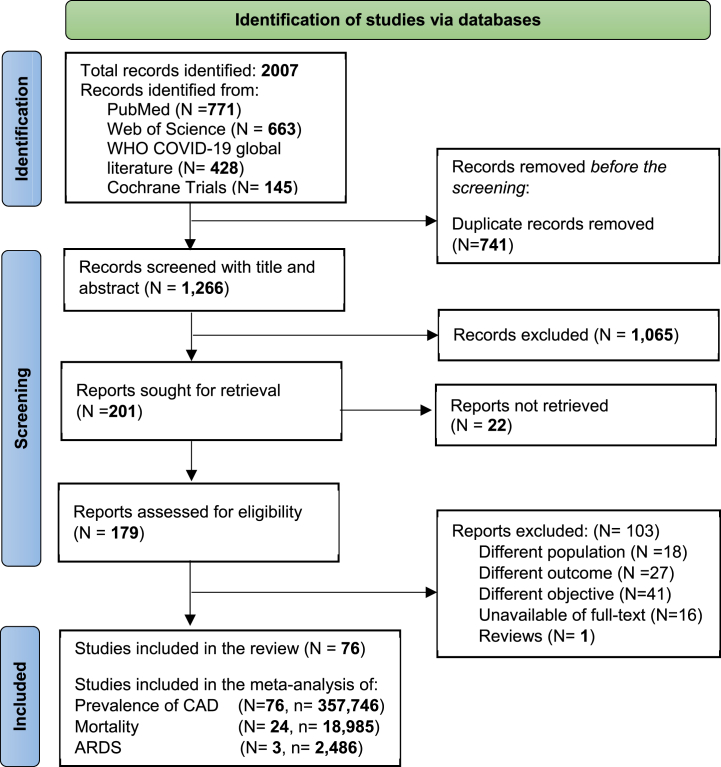
The PRISMA flowchart showing the studies included in the systematic review.

The results of our search are summarized in Fig. 1. Overall, we retrieved 2007 records from different databases. Prior to the screening, 741 records were removed due to duplication, leaving a total of 1266 records to be screened. After screening the title and abstract, 201 reports were kept and sought for retrieval. Then, the full-texts of 103 articles were excluded leaving 76 articles to be included in this analysis (Fig. 1). Reasons for excluding articles after full-text screening were provided in the supplementary appendix (Appendix A_ Tables A.6- A.7).

### 3.2 Participants and study settings

A total of 76 articles with 357,746 participants were included in this systematic review and meta-analysis. Forty-one percent (n = 31) of the included articles were conducted in Europe and 33% (n = 25) in the USA, while the rest were conducted in Asian countries (n = 20). Of the 76 included studies, 13 were cross-sectional, 7 were cohorts, and 1 was case-control, with the remainder being retrospective in design. No single randomized trial met our inclusion criteria. Characteristics of included articles are provided in Table 1.

**Table 1:** Characteristics of the included studies (N = 76).

NO	Author, Year	Country	Study type	Sample Size	Age Mean ± SD/median (IQR)	Male n (%)	SARS-2 method of confirmation
1	Loffi, 2020 [22]	Italy	Retrospective	1252	64.7 ± 15.5	798 (63.70)	RT-PCR
2	Yuan, 2021 [23]	China	Retrospective	2886	59.1± NR	1402 (49.1)	NR
3	Peterson, 2021 [24]	USA	Retrospective	355	66.21 ± 14.21	181 (51)	RT-PCR
4	Park, 2021 [25]	Korea	Retrospective	2269	55.5 ± 20.2	814 (35.9)	PCR
5	Yamada, 2021 [26]	Japan	Cross-sectional	693	68.3 ± 14.9	449 (64.8)	PCR
6	Bonnet, 2021 [27]	France	Cohort study	2878	66.6 ± 17.0	1666 (57.8)	RT_PCR or CT
7	Langnau, 2021 [28]	Germany	Cross-sectional	122	63.5 (47–79)	122 (100)	RT-PCR
8	Li, 2020 [29]	China	Retrospective	83	43 (32–64)	34 (50)	Laboratory-confirmed and CT
9	Toprak, 2021 [30]	Turkey	Cross-sectional	298	58.33 ± 15.52	156 (52.3)	RT-PCR
10	Gunawardene, 2021 [31]	Germany	Prospective cohort	414	68 ± 18	339 (58.24)	Positive for SARSCoV-2 using a reliable test method
11	Cheng, 2021 [32]	China	Retrospective	1157	NR	586 (50.6)	Laboratory-confirmed COVID-19 according to WHO guidelines
12	Akıllı, 2021 [33]	Turkey	Cross-sectional	582	NR	339 (58.2)	NR
13	Inciardi, 2020 [34]	Italy	Cross-sectional	99	67 ± 12	80 (80.8)	PCR
14	Xie, 2020 [35]	China	Cross-sectional	62	66 (53.3–73)	27 (43.5)	RT-PCR
15	Jalali, 2021 [36]	Iran	Cohort study	196	65 (52–67)	104 (53.1)	RT-PCR
16	Xiong, 2020 [37]	China	Cross-sectional	116	58.5 (47–69)	80 (69.0)	RT-PCR
17	Zhang, 2020 [38]	China	Retrospective	541	53.25 ± 16.29	183 (33.8)	RT-PCR
18	Li, 2021 [39]	China	Cohort study	2954	60 (50–68)	1493 (50.5)	RT-PCR
19	Aoun, 2021 [40]	Lebanon	Retrospective	231	61.46 ± 13.99	128 (55.4)	RT-PCR
20	Scoccia, 2021 [41]	Italy	Retrospective	1625	69 (58–77)	1092 (67.2)	laboratory-confirmed SARS-CoV-2
21	Gottlieb, 2020 [42]	USA	Retrospective	1483	56 (44–68)	792 (53.4)	RT-PCR
22	Birtay, 2021 [43]	Turkey	Retrospective	124	79 (64–91)	62 (50)	RT_PCR
23	Angeli, 2020 [44]	Italy	Retrospective	954	72 (59–85)	543 (57)	RT-PCR
24	Brojakowska, 2021 [45]	USA	Retrospective	7032	NR	NR	RT-PCR
25	Pezel, 2021 [46]	France	Retrospective	481	68.4 ± 9.6	295 (61.3)	RT-PCR
26	Salinas, 2021 [47]	Spain	Case control	316	68 (58–78)	224 (70.9)	PCR
27	Xiong, 2020 [48]	China	Retrospective	472	43 (32–53.5)	250 (53)	RT-PCR
28	Caliskan, 2020 [49]	Turkey	Retrospective	565	48.0 ± 19.7	NR	PCR
29	Chacko, 2021 [50]	USA	Retrospective	255	65.4 ± 15.2	130 (51)	RT-PCR
30	Khawaja, 2021 [51]	U.K	Retrospective	498	67.4 ± 16.1	310 (62.2)	RT-PCR
31	Barman, 2021 [52]	Turkey	Retrospective	607	62.5 ± 14.3	334 (55)	RT-PCR
32	Mithal, 2021 [53]	India	Cross-sectional	401	54 (19–92)	276 (68.8)	RT-PCR
33	Terlecki, 2021 [54]	Poland	Retrospective	1729	63 (50–75)	886 (51.2)	NR
34	Cen, 2020 [55]	China	Cohort study	1007	61 (49–68)	493 (49)	RT-PCR
35	Keskin, 2021 [56]	Turkey	Cross-sectional	37	66 (27–84)	22 (59.5)	RT-PCR
36	Girardin, 2021 [57]	USA	Retrospective	4446	65.7 ± 16.4	1166 (26.2)	SARSCoV2 Xpert Xpress assay
37	Nguyen, 2020 [58]	USA	Retrospective	689	55 (40–68)	296 (43)	NR
38	Ko, 2020 [59]	USA	Retrospective	5416	NR	22,847 (53)	laboratory-confirmed COVID-19
39	Nicholson, 2021 [60]	USA	Retrospective	1042	64 (53–75)	592 (56.8)	RT-PCR
40	Ayten, 2020 [61]	Turkey	Retrospective	73	56.9 ± 13.3	47 (64.4)	RT-PCR
41	Aldabagh, 2021 [62]	USA	Retrospective	450	66.4 ± 13.1	271 (60.2)	RT_PCR
42	Ciceri, 2020 [63]	Italy	Cohort study	410	65 (56–75)	299 (72.9)	RT-PCR
43	Kantroo, 2021 [64]	India	Retrospective	1192	50 (35–61)	832 (70)	RT-PCR
44	Guarin, 2021 [65]	USA	Retrospective	275	64.69 ± 14.64	142 (51.6)	RT-PCR
45	Wei, 2020 [66]	China	Retrospective	566	61.5 (NR)	267 (47.2)	laboratory-confirmed COVID-19
46	Weizman, 2021 [67]	France	Retrospective	2878	66.9 ± 17	1666 (57.9)	RT-PCR
47	Mousseaux, 2021 [68]	France	Retrospective	169	65.6 ± 18.8	118 (69.8)	RT-PCR
48	Ciprian, 2021 [69]	Italy	Retrospective	109	71 (60–81)	73 (67)	laboratory-confirmed SARSCoV-2 infection and clinical and radiological signs of COVID-19
49	Poterucha, 2020 [70]	USA	Retrospective	887	64.1 ± 17.2	513 (57.8)	RT-PCR
50	Zhang, 2021 [71]	China	Retrospective	541	61.4 ± 13.6	255 (47.1)	
51	Lip, 2021 [72]	USA	Prospective cohort	280,592	72.5 ± 9.9	115,629 (41.2)	NR
52	Russo, 2021 [73]	Italy	Retrospective	467	66.88 ± 14.55	294 (63)	RT-PCR
53	Russo, 2020 [74]	Italy	Cross-sectional	192	67.7 ± 15.2	115 (60)	RT-PCR
54	Gsblom, 2021 [75]	Finland	Retrospective	585	57(46–70)	316 (54)	RT-PCR
55	Turagam, 2020 [76]	USA	Retrospective	140	61 (48–74)	102 (72.9)	RT-PCR
56	Nanda, 2021 [77]	USA	Retrospective	1169	43.9 ± 17.6	575 (49.2)	RT-PCR
57	Gupta, 2021 [78]	USA	Retrospective	180	68 ± 59-80	98 (54.4)	RT-PCR
58	Adrish, 2020 [79]	USA	Retrospective	1173	63 (53–73)	720 (61.4)	RT-PCR
59	Raghavan, 2021 [80]	India	Retrospective	845	NR	553 (65.4)	RT-PCR
60	Linschoten, 2020 [81]	Netherlands	Retrospective	3011	67 (56–76)	1890 (62.8)	NR
61	Koutroumpakis, 2021 [82]	USA	Retrospective	514	59 (48–71)	246 (47.9)	RT-PCR
62	Valenzuela, 2020 [83]	USA	Retrospective	2039	52 (38–65)	1081 (53)	RT-PCR
63	Gupta, 2021 [84]	USA	Retrospective	529	70 (61–80)	286 (54.1)	RT-PCR
64	Violi, 2021 [85]	Italy	Retrospective	373	67.4 ± 16.8	228 (61.1)	RT-PCR and CT
65	Erben, 2021 [86]	USA	Retrospective	915	60.8 ± 17.0	520 (56.8)	PCR or serology testing
66	Lodiqiani, 2020 [87]	Italy	Retrospective	388	66 (57–75)	264 (68)	laboratory-proven COVID-19
67	Guner, 2020 [88]	Turkey	Cross-sectional	222	50.6 ± 16.5	132 (59.5)	PCR
68	Maeda, 2021 [89]	USA	Retrospective	181	64 ± 16.6	101 (55.8)	RT-PCR
69	Lala, 2020 [90]	USA	Retrospective	2736	66.40 ± 15.80	1630 (59.6)	laboratory-confirmed SARS-CoV-2
70	Kini, 2021 [16]	USA	Retrospective	4695	64 ± 16.5	2643 (56.3)	laboratory-confirmed SARS-CoV2
71	Gormez, 2021 [91]	Turkey	Retrospective	103	45 (39–52)	50 (48.5)	RT-PCR
72	Gupta, 2020 [92]	USA	Cross-sectional	2215	60.5 ± 14.5	1436 (64.8)	Laboratory-confirmed COVID-19 (detected by nasopharyngeal or oropharyngeal swab)
73	Al-Ani, 2022 [93]	Iraq	Cross-sectional	101	53.05 ± 15.16	69 (68.3)	RT_PCR
74	Atlas, 2021 [94]	Turkey	Retrospective	102	69.1 ± 14.3	71 (69.6)	NR
75	Banoei, 2021 [95]	USA	Retrospective	250	69.34 ± 13.69	130 (52)	PCR
76	Akhavizadegan, 2021 [96]	Iran	Retrospective	1CE12	67.55 ± 12.57	77 (68.8)	RT_PCR

CT=Chest computed tomography; IQR=Interquartile range; N=Number of participants; RT-PCR=Real time_polymerase chain reaction; SD=Standard deviation; USA=United States of America; who = World Health Organization.

N, number of studies; n, cohort size; WHO, World Health Organization.

### 3.3 Quality and risk of bias assessment

Overall, the risk of bias in this meta-analysis of 76 studies was relatively low and only 4% (n = 3) of the studies were reported to have a high risk of bias (Appendix C_ fig. C1).

### 3.4 Prevalence of CAD among hospitalized COVID-19 patients

**Fig. 2:**
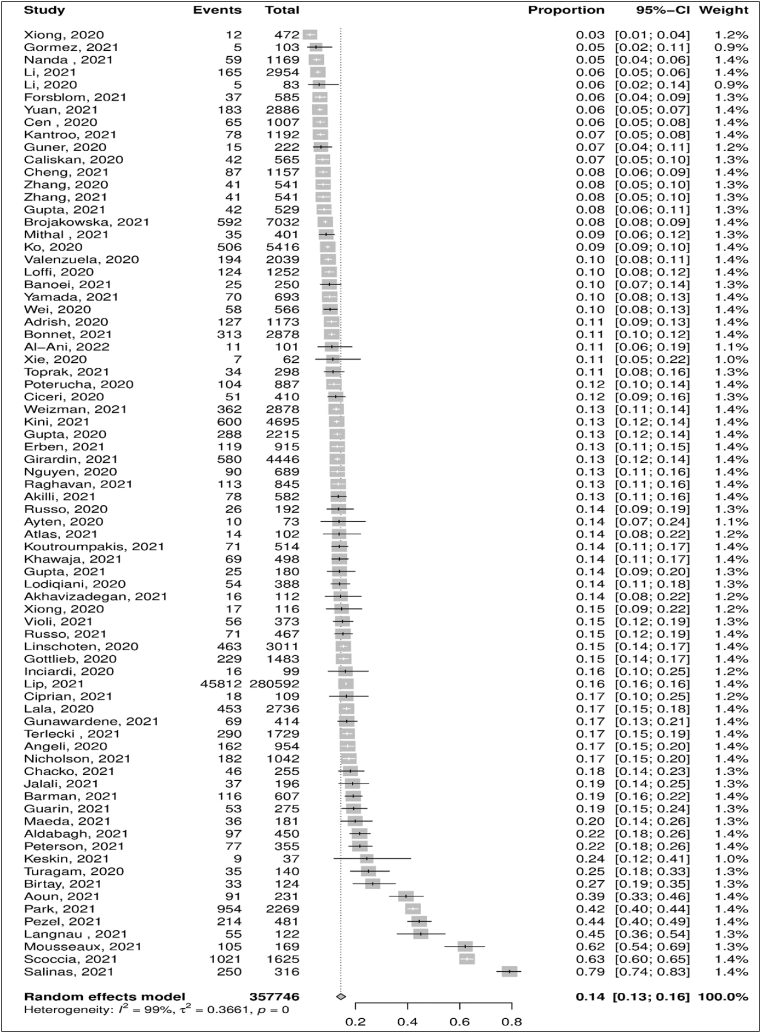
Forest plot estimating pooled prevalence of Coronary Artery Disease (CAD) among COVID-19 hospitalized patients.

The prevalence of CAD among hospitalized COVID-19 patients was reported in 76 observational studies with a total participant count of 357,746. Using the random-effect model (I^2^ = 98.7% [95% CI: 98.5–98.8]), the pooled prevalence of CAD was 14.4% [95% CI: 12.7–16.2] (Fig. 2). A sensitivity analysis was performed to investigate further potential sources of heterogeneity in prevalence. Using leave-one-out analysis, three studies were identified as influential studies with an effect of 3.9% [41], 5.6% [47], and 3.6% [68] above the overall estimate of CAD prevalence. The pooled prevalence got adjusted to 13.2% [95% CI: 11.9–14.6] with an I^2^ of 97.8% [95% CI: 97.5–98.0] after removing these 3 influential studies and studies with a high risk of bias [47,79,93](Appendix C_ fig. C.2 and C.3).

### 3.5 Mortality among hospitalized COVID-19 patients with CAD

**Fig. 3:**
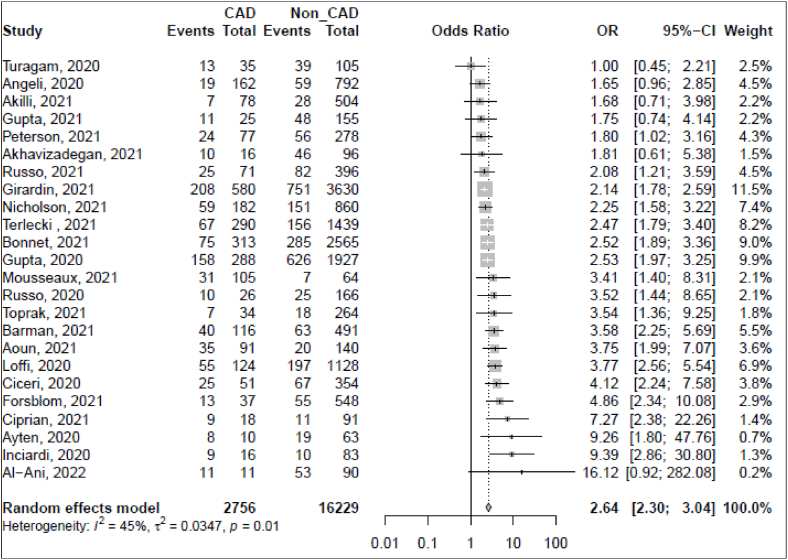
Forest plot for the association of CAD and mortality among hospitalized COVID-19 patients.

Data on mortality among hospitalized COVID-19 patients with CAD were available in twenty-four observational studies, with a total of 18,985 participants. Using a random-effect model (I^2^ = 44.7% [95% CI: 10.6–65.9]), the pooled odds ratio (OR) of mortality among hospitalized COVID-19 patients with CAD was 2.64 [95% CI: 2.30–3.04] (Fig. 3). A sensitivity analysis was performed to investigate further potential sources of heterogeneity for the estimated association. Using leave-one-out analysis, the highest estimated OR would be adjusted to 2.72 with an I^2^ of 42% and the lowest would be adjusted to 2.52 with an I^2^ of 41% after removing Girardin [57] and Loffi [22] studies, respectively (Appendix C_ fig. C4). Further analysis was performed, removing the high risk of bias study [93]. The adjusted OR was 2.63 [95% CI: 2.29–3.02] with an I^2^ of 45.0% [95% CI: 10.2–66.3] (Appendix C_ fig. C.5).

**Fig. 4:**
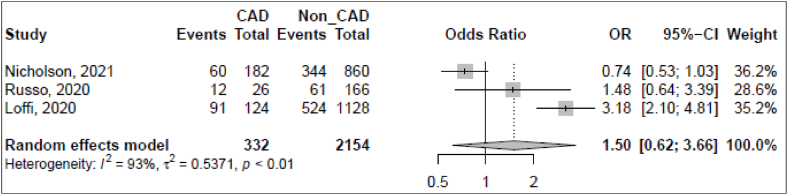
Forest plot for the association of CAD and ARDS among hospitalized COVID-19 patients.

### 3.6 ARDS among hospitalized COVID-19 patients with CAD

Out of 76 studies, we found only three studies reviewing the association between ARDS and CAD among hospitalized COVID-19 patients. With a total of 2486 participants reported for these studies, the pooled estimated association of ARDS with CAD was 1.50 [95% CI: 0.62–3.66). A high heterogeneity was reported among included studies (I^2^ = 93.0% [95% CI: 83.0–97.1]) (Fig. 4).

### 3.7 Subgroup analysis

We further evaluated the differences caused by geographical variations as a sub-group analysis. The pooled prevalence of CAD among hospitalized COVID-19 patients in European studies was 18.2% ([95% CI: 13.3–24.2], I^2^ = 98.9%), 13.5% ([95% CI: 12.0–15.1], I^2^ = 97.1%) in US studies and about 10.4% ([95% CI: 6.4–16.3], I^2^ = 99.0%) in Asian studies. The between-group analysis was not significant (χ^2^ = 4.69, *p* = 0.10). However, the prevalence was 4.7% higher among the European studies compared to US studies and 7.8% higher compared to the Asian studies (Appendix C_ fig. C.6).

For the association between mortality and CAD among hospitalized COVID-19 patients, the subgroup analysis showed that a higher pooled OR of mortality was among studies conducted in Asian countries (OR = 3.29 [95% CI: 1.88–5.76], I^2^ = 20%) compared to studies conducted in the US (OR = 2.18 [95% CI: 1.91–2.48], I^2^ = 14%) and Europe (OR = 3.07 [95% CI: 2.52–3.74], I^2^ = 40%). The estimated associations for the subgroup analysis were significant (χ^2^ = 9.16, *p* = 0.01) (Appendix C_ fig. C.7).

## 4 Discussion

### 4.1 Prevalence of CAD among hospitalized COVID-19 patients

Our review of 76 studies estimated a pooled prevalence of CAD in 14% of the patients with COVID-19 hospital admissions which varied by region. In comparison to Europe and the United States of America, despite the limited number of Asian studies, people living in Asia had a lower prevalence of CAD among patients infected with SARS-CoV2. Although the risk for mortality due to COVID-19 was two times higher among those with pre-existing CAD. Despite strong evidence for this relationship, the degree of certainty was relatively low. Likewise, our analysis revealed a 50% higher of ARDS among COVID-19 patients with CAD, although the association did not reach statistical significance.

### 4.2 CAD and mortality association among hospitalized COVID-19 patients

Numerous studies have reported that COVID-19 patients with CAD are at a higher risk of mortality [[97], [98], [99], [100]]. A high increment in the prevalence of mortality was observed across included studies, with a prevalence rate ranging between 9% [33] and 100% [93]. A possible explanation can be drawn from the fact that the S1 sub-unit of the spike protein of severe acute respiratory syndrome (SARS-CoV-2) binds to the host cells through an angiotensin-converting enzyme (ACE2) which triggers coagulation pathways, vasoconstriction, myocarditis and fibrosis [101]. The release of ACE2 is exacerbated as the cardiac condition progresses to systolic dysfunction and may lead to fatal outcomes, one being mortality. All of these increases the chance of severe outcomes among COVID-19 patients, one of which is mortality [102].

Our analysis revealed that the pooled estimated association of mortality among COVID-19 patients with CAD was high (OR = 2.62). Our finding was in harmony with the findings of a recent systematic review which reported that 22.9% of non-survival COVID-19 patients had CAD [4]. Studies show a higher rate of admission to the intensive unit and a more severe condition were observed among COVID-19 patients with CAD compared to those without CAD [4]. A multicenter cohort study reported that CAD was independently related to the higher risk of 28-days in hospital mortality (OR = 1.47, 95% CI: 1.07–2.02) [92]. Similarly another study reported that the likelihood of COVID-19 mortality was relatively higher at older ages compared to younger ages [57]. The study also suggested that the presence of comorbidities including CAD, diabetes mellitus, and hypertension may be attributed to the higher age-related mortality in COVID-19 patients [57]. Across included studies, the median age of the participants ranged between 57 and 72 years old, which might give justification for the high estimated association that was revealed by our analysis, as suggested by others [13,31,36,42,60,74,103].

Based on the geographical distribution, the subgroup meta-analysis showed the highest pooled estimate of mortality with CAD in Asian studies compared to those conducted in Europe and the US. However, a lower pooled estimate of mortality was observed in Asian studies after removing the study with a high risk of bias [93](OR = 2.99 [1.5524; 5.7895], I^2^ 21.9%). As a result, taking into account the pooled estimate of mortality after removing the influential study could yield a reliable estimate. The subgroup analysis was significant and the possible reason for this variation might be due to the healthcare services disturbed across the globe, which might have serious implications for the prognosis of COVID-19 patients with CAD [104].

#### 4.2.1 CAD and ARDS association among hospitalized COVID-19 patients

COVID-19 patients with prior CAD or heart failure are more vulnerable to developing adverse events or having severe clinical phenotypes [4]. A recent study reported that patients with CAD had an absolute increase of about 27% in the incidence of ARDS among COVID-19 patients during hospitalization [22]. Although, respiratory dysregulation has been causally linked to SARS-CoV-1 and Middle East respiratory syndrome (MERS). However, multifactorial etiology complicates the understanding of physiological links and pathogenesis involved in the development of respiratory changes and mortality associated with SARS-CoV-2 infection [105]. It is likely that genomic similarity between SARS-CoV-1 and SARS-CoV-2 could be a potential area for scientific inquiry and investigation of precise mechanisms and etiological links to the proliferation of inflammatory mediators and cellular pathways leading to the damage of pulmonary vessels and respiratory complications [106]. Despite observing an association between the occurrence of ARDS and CAD, albeit non-significant, our findings indicate a 50% increased likelihood of ARDS among patients with comorbid conditions such as CAD and COVID-19 infection [107]. Yet, no ARDS interventions that are used are effective enough to employ the advantages of early ARDS detection. Due to these considerations, identifying a reliable biomarker for ARDS is a challenge [107]. Hence, further knowledge of the impact of CAD on the occurrence of ARDS among hospitalized COVID-19 patients is needed.

### 4.3 Limitations

Our review included observational studies which were either cross-sectional in nature or were retrospectively conducted and the element of residual confounding and selection bias cannot be ruled out. Cause and effect link cannot be studied between mortality rate and the existence of CAD among hospitalized COVID-19 patients as none of the included studies were randomized control trial.

In some of the studies, the ascertainment of CAD relied on subjective assessments, which introduces the risk of non-differential misclassification and measurement errors. We would like to acknowledge the limitations associated with diagnosing CAD as a categorical variable. The categorical nature of CAD diagnosis is arbitrary and somewhat restrictive in terms of clinical assumptions.

Furthermore, our review did not evaluate the effects of existing CAD on the length of hospital stay among COVID-19 patients, which could have served as a proxy indicator for disease severity, complications, and its impact on the health system.

Additionally, the studies included could not ascertain the temporal links on whether infectivity with COVID-19 increases the length of hospital stays and adverse health outcomes or whether it is the existing chronic condition or comorbidity that can lead to multi-morbidity and mortality among COVID-19 patients. Despite acknowledging the geographical variations and implications of the prevalence of cardiac morbidities among COVID-19 patients. The analysis might have adjusted for differences in healthcare infrastructure which differs between these regions, however, we could not control for disparities in the severity of diseases caused by different variants of coronavirus infection and how this affects the relationship between mortality due to CAD among COVID-19.

The available data limited our ability to determine if severe outcomes varied based on the type and duration of CAD. Additionally, the majority of the included studies were conducted in Europe and the USA, limiting the generalizability of our findings to other regions. Testing protocols varied across the world and could not be controlled for in this study.

Finally, our literature search was restricted to English-language sources only, which introduces the risk of publication bias and the exclusion of evidence from unpublished papers and non-English language sources.

## 5 Conclusion

The existence of CAD was found relatively associated with increased severity and mortality in COVID-19 patients in our study. This study revealed a pooled prevalence of 14% of CAD among hospitalized COVID-19 patients. CAD and COVID-19 share the existence of ARDS, which might be the main reason for increased mortality among those patients who suffer from both diseases at once. Further research on determining how CAD affects the likelihood of ARDS in COVID-19 hospitalized patients is needed.

## Author contribution statement

Mohammed Merzah: Conceived and designed the experiments; Analyzed and interpret the data; Performed the experiments; Contributed analysis tools; Wrote the paper.

Atiya Abdul Karim: Performed the experiments; Analyzed and interpreted the data; Contributed analysis tools; Wrote the paper.

Dahy Sulaiman: Performed the experiments; Contributed analysis tools; Wrote the paper.

Mazin E. Khalil, Stany Mathew, Yasir Almuzaini, Shima Hashemi, Salina Khatoon, Sabyasachi Gupta, Mohima Benojir Hoquej: Performed the experiments; Wrote the paper.

## Data availability statement

Data included in article/supp. material/referenced in article.

## Declaration of competing interest

The authors declare that they have no known competing financial interests or personal relationships that could have appeared to influence the work reported in this paper.
