# Supplementary material for: A systematic review and meta-analysis on the prevalence and impact of coronary artery disease in hospitalized COVID-19 patients
Source: Heliyon. 2023 Aug 25;9(9):e19493. doi: 10.1016/j.heliyon.2023.e19493 (PMC10480662; doi:10.1016/j.heliyon.2023.e19493)
Supplement: Multimedia component 1 [file mmc1.docx]

**A systematic review and meta-analysis on the prevalence and impact of coronary artery disease in hospitalized COVID-19 patients.**

**Supplementary file**

**Contents**

[A. Search Strategy used in different databases and search results 1](#_Toc113240743)

[1. PubMed 1](#_Toc113240744)

[Table S 1 Keywords used for PubMed search 1](#_Toc113240745)

[Table S 2 PubMed search results 2](#_Toc113240746)

[2. Web of Science 51](#_Toc113240747)

[Table S 3 Web of Science search results 51](#_Toc113240748)

[3. World Health Organization Covid-19 Global literature on coronavirus disease 51](#_Toc113240749)

[Table S 4 WHO Covid-19 global literature on coronavirus disease search results 51](#_Toc113240750)

[4. Cochrane Library- Trials 51](#_Toc113240751)

[Table S 5 Cochrane Library search results 51](#_Toc113240752)

[B. Articles excluded from the review 53](#_Toc113240753)

[Table S 6 Reason for exclusion with numbers and percentages 53](#_Toc113240754)

[Table S 7 Details of excluded articles from the review 54](#_Toc113240755)

# A. Search Strategy used in different databases and search results

## 1. PubMed

Date of search = 21-03-2022

Final search result = 771

### Table S 1 Keywords used for PubMed search

| **BLOCKS** |  |
| --- | --- |
| **1** | **Covid-19**   1. SARS-CoV-2 (MeSH) 2. COVID-19 (MeSH) 3. Coronavirus Infections (MeSH) 4. 2019-nCoV (keyword) 5. coronavirus disease 2019 (keyword)   Filter- Humans, English, 2019:2022 (Publishing date) |
| **2** | **Prevalence**   1. Prevalence (MeSH) 2. Risk Factors (MeSH) 3. Risk Assessment (MeSH) 4. Epidemiology (MeSH) 5. Mortality (MeSH)   Filter- Humans, English |
| **3** | **CAD**   1. Coronary Artery Disease (MeSH) 2. Cardiovascular Diseases (MeSH) 3. Acute Coronary Syndrome (MeSH)   Filter- Humans, English |

### Table S 2 PubMed search results

| **Search number** | **Query** | **Filters** | **Search Details** | **Results** |
| --- | --- | --- | --- | --- |
| 1 | SARS-CoV-2 |  | "sars cov 2"[MeSH Terms] OR "sars cov 2"[All Fields] OR "sars cov 2"[All Fields] | 1,52,702 |
| 2 | COVID-19 |  | "covid 19"[All Fields] OR "covid 19"[MeSH Terms] OR "covid 19 vaccines"[All Fields] OR "covid 19 vaccines"[MeSH Terms] OR "covid 19 serotherapy"[All Fields] OR "covid 19 serotherapy"[Supplementary Concept] OR "covid 19 nucleic acid testing"[All Fields] OR "covid 19 nucleic acid testing"[MeSH Terms] OR "covid 19 serological testing"[All Fields] OR "covid 19 serological testing"[MeSH Terms] OR "covid 19 testing"[All Fields] OR "covid 19 testing"[MeSH Terms] OR "sars cov 2"[All Fields] OR "sars cov 2"[MeSH Terms] OR "severe acute respiratory syndrome coronavirus 2"[All Fields] OR "ncov"[All Fields] OR "2019 ncov"[All Fields] OR (("coronavirus"[MeSH Terms] OR "coronavirus"[All Fields] OR "cov"[All Fields]) AND 2019/11/01:3000/12/31[Date - Publication]) | 2,38,893 |
| 3 | Coronavirus Infections |  | "coronavirus infections"[MeSH Terms] OR ("coronavirus"[All Fields] AND "infections"[All Fields]) OR "coronavirus infections"[All Fields] | 1,63,082 |
| 4 | 2019-nCoV |  | "sars cov 2"[MeSH Terms] OR "sars cov 2"[All Fields] OR "2019 ncov"[All Fields] | 1,53,151 |
| 5 | SARS-CoV-2 OR COVID-19 OR Coronavirus Infections OR 2019-nCoV OR Pandemics OR coronavirus disease 2019 OR Hospitalization |  | "sars cov 2"[MeSH Terms] OR "sars cov 2"[All Fields] OR "sars cov 2"[All Fields] OR ("covid 19"[All Fields] OR "covid 19"[MeSH Terms] OR "covid 19 vaccines"[All Fields] OR "covid 19 vaccines"[MeSH Terms] OR "covid 19 serotherapy"[All Fields] OR "covid 19 serotherapy"[Supplementary Concept] OR "covid 19 nucleic acid testing"[All Fields] OR "covid 19 nucleic acid testing"[MeSH Terms] OR "covid 19 serological testing"[All Fields] OR "covid 19 serological testing"[MeSH Terms] OR "covid 19 testing"[All Fields] OR "covid 19 testing"[MeSH Terms] OR "sars cov 2"[All Fields] OR "sars cov 2"[MeSH Terms] OR "severe acute respiratory syndrome coronavirus 2"[All Fields] OR "ncov"[All Fields] OR "2019 ncov"[All Fields] OR (("coronavirus"[MeSH Terms] OR "coronavirus"[All Fields] OR "cov"[All Fields]) AND 2019/11/01:3000/12/31[Date - Publication])) OR ("coronavirus infections"[MeSH Terms] OR ("coronavirus"[All Fields] AND "infections"[All Fields]) OR "coronavirus infections"[All Fields]) OR ("sars cov 2"[MeSH Terms] OR "sars cov 2"[All Fields] OR "2019 ncov"[All Fields]) OR ("pandemic s"[All Fields] OR "pandemically"[All Fields] OR "pandemicity"[All Fields] OR "pandemics"[MeSH Terms] OR "pandemics"[All Fields] OR "pandemic"[All Fields]) OR ("covid 19"[MeSH Terms] OR "covid 19"[All Fields] OR "coronavirus disease 2019"[All Fields]) OR ("hospital s"[All Fields] OR "hospitalisation"[All Fields] OR "hospitalization"[MeSH Terms] OR "hospitalization"[All Fields] OR "hospitalising"[All Fields] OR "hospitality"[All Fields] OR "hospitalisations"[All Fields] OR "hospitalised"[All Fields] OR "hospitalizations"[All Fields] OR "hospitalized"[All Fields] OR "hospitalize"[All Fields] OR "hospitalizing"[All Fields] OR "hospitals"[MeSH Terms] OR "hospitals"[All Fields] OR "hospital"[All Fields]) | 62,57,678 |
| 6 | SARS-CoV-2 OR COVID-19 OR Coronavirus Infections OR 2019-nCoV OR coronavirus disease 2019 OR Hospitalization |  | "sars cov 2"[MeSH Terms] OR "sars cov 2"[All Fields] OR "sars cov 2"[All Fields] OR ("covid 19"[All Fields] OR "covid 19"[MeSH Terms] OR "covid 19 vaccines"[All Fields] OR "covid 19 vaccines"[MeSH Terms] OR "covid 19 serotherapy"[All Fields] OR "covid 19 serotherapy"[Supplementary Concept] OR "covid 19 nucleic acid testing"[All Fields] OR "covid 19 nucleic acid testing"[MeSH Terms] OR "covid 19 serological testing"[All Fields] OR "covid 19 serological testing"[MeSH Terms] OR "covid 19 testing"[All Fields] OR "covid 19 testing"[MeSH Terms] OR "sars cov 2"[All Fields] OR "sars cov 2"[MeSH Terms] OR "severe acute respiratory syndrome coronavirus 2"[All Fields] OR "ncov"[All Fields] OR "2019 ncov"[All Fields] OR (("coronavirus"[MeSH Terms] OR "coronavirus"[All Fields] OR "cov"[All Fields]) AND 2019/11/01:3000/12/31[Date - Publication])) OR ("coronavirus infections"[MeSH Terms] OR ("coronavirus"[All Fields] AND "infections"[All Fields]) OR "coronavirus infections"[All Fields]) OR ("sars cov 2"[MeSH Terms] OR "sars cov 2"[All Fields] OR "2019 ncov"[All Fields]) OR ("covid 19"[MeSH Terms] OR "covid 19"[All Fields] OR "coronavirus disease 2019"[All Fields]) OR ("hospital s"[All Fields] OR "hospitalisation"[All Fields] OR "hospitalization"[MeSH Terms] OR "hospitalization"[All Fields] OR "hospitalising"[All Fields] OR "hospitality"[All Fields] OR "hospitalisations"[All Fields] OR "hospitalised"[All Fields] OR "hospitalizations"[All Fields] OR "hospitalized"[All Fields] OR "hospitalize"[All Fields] OR "hospitalizing"[All Fields] OR "hospitals"[MeSH Terms] OR "hospitals"[All Fields] OR "hospital"[All Fields]) | 62,34,715 |
| 7 | Prevalence OR Risk Factors OR Risk Assessment OR Epidemiology OR Mortality |  | "epidemiology"[MeSH Subheading] OR "epidemiology"[All Fields] OR "prevalence"[All Fields] OR "prevalence"[MeSH Terms] OR "prevalance"[All Fields] OR "prevalences"[All Fields] OR "prevalence s"[All Fields] OR "prevalent"[All Fields] OR "prevalently"[All Fields] OR "prevalents"[All Fields] OR ("risk factors"[MeSH Terms] OR ("risk"[All Fields] AND "factors"[All Fields]) OR "risk factors"[All Fields]) OR ("risk assessment"[MeSH Terms] OR ("risk"[All Fields] AND "assessment"[All Fields]) OR "risk assessment"[All Fields]) OR ("epidemiologies"[All Fields] OR "epidemiology"[MeSH Subheading] OR "epidemiology"[All Fields] OR "epidemiology"[MeSH Terms] OR "epidemiology s"[All Fields]) OR ("mortality"[MeSH Terms] OR "mortality"[All Fields] OR "mortalities"[All Fields] OR "mortality"[MeSH Subheading]) | 47,49,964 |
| 8 | Coronary Artery Disease OR Cardiovascular Diseases OR Acute Coronary Syndrome OR Chronic Disease OR Disease Attributes OR Respiratory Distress Syndrome OR ARDS |  | "coronary artery disease"[MeSH Terms] OR ("coronary"[All Fields] AND "artery"[All Fields] AND "disease"[All Fields]) OR "coronary artery disease"[All Fields] OR ("cardiovascular diseases"[MeSH Terms] OR ("cardiovascular"[All Fields] AND "diseases"[All Fields]) OR "cardiovascular diseases"[All Fields]) OR ("acute coronary syndrome"[MeSH Terms] OR ("acute"[All Fields] AND "coronary"[All Fields] AND "syndrome"[All Fields]) OR "acute coronary syndrome"[All Fields]) OR ("chronic disease"[MeSH Terms] OR ("chronic"[All Fields] AND "disease"[All Fields]) OR "chronic disease"[All Fields]) OR ("disease attributes"[MeSH Terms] OR ("disease"[All Fields] AND "attributes"[All Fields]) OR "disease attributes"[All Fields]) OR ("respiratory distress syndrome"[MeSH Terms] OR ("respiratory"[All Fields] AND "distress"[All Fields] AND "syndrome"[All Fields]) OR "respiratory distress syndrome"[All Fields]) OR ("respiratory distress syndrome"[MeSH Terms] OR ("respiratory"[All Fields] AND "distress"[All Fields] AND "syndrome"[All Fields]) OR "respiratory distress syndrome"[All Fields] OR "ards"[All Fields]) | 43,53,773 |
| 9 | ((SARS-CoV-2 OR COVID-19 OR Coronavirus Infections OR 2019-nCoV OR coronavirus disease 2019 OR Hospitalization) AND (Prevalence OR Risk Factors OR Risk Assessment OR Epidemiology OR Mortality)) AND (Coronary Artery Disease OR Cardiovascular Diseases OR Acute Coronary Syndrome OR Chronic Disease OR Disease Attributes OR Respiratory Distress Syndrome OR ARDS) |  | ("sars cov 2"[MeSH Terms] OR "sars cov 2"[All Fields] OR "sars cov 2"[All Fields] OR ("covid 19"[All Fields] OR "covid 19"[MeSH Terms] OR "covid 19 vaccines"[All Fields] OR "covid 19 vaccines"[MeSH Terms] OR "covid 19 serotherapy"[All Fields] OR "covid 19 serotherapy"[Supplementary Concept] OR "covid 19 nucleic acid testing"[All Fields] OR "covid 19 nucleic acid testing"[MeSH Terms] OR "covid 19 serological testing"[All Fields] OR "covid 19 serological testing"[MeSH Terms] OR "covid 19 testing"[All Fields] OR "covid 19 testing"[MeSH Terms] OR "sars cov 2"[All Fields] OR "sars cov 2"[MeSH Terms] OR "severe acute respiratory syndrome coronavirus 2"[All Fields] OR "ncov"[All Fields] OR "2019 ncov"[All Fields] OR (("coronavirus"[MeSH Terms] OR "coronavirus"[All Fields] OR "cov"[All Fields]) AND 2019/11/01:3000/12/31[Date - Publication])) OR ("coronavirus infections"[MeSH Terms] OR ("coronavirus"[All Fields] AND "infections"[All Fields]) OR "coronavirus infections"[All Fields]) OR ("sars cov 2"[MeSH Terms] OR "sars cov 2"[All Fields] OR "2019 ncov"[All Fields]) OR ("covid 19"[MeSH Terms] OR "covid 19"[All Fields] OR "coronavirus disease 2019"[All Fields]) OR ("hospital s"[All Fields] OR "hospitalisation"[All Fields] OR "hospitalization"[MeSH Terms] OR "hospitalization"[All Fields] OR "hospitalising"[All Fields] OR "hospitality"[All Fields] OR "hospitalisations"[All Fields] OR "hospitalised"[All Fields] OR "hospitalizations"[All Fields] OR "hospitalized"[All Fields] OR "hospitalize"[All Fields] OR "hospitalizing"[All Fields] OR "hospitals"[MeSH Terms] OR "hospitals"[All Fields] OR "hospital"[All Fields])) AND ("epidemiology"[MeSH Subheading] OR "epidemiology"[All Fields] OR "prevalence"[All Fields] OR "prevalence"[MeSH Terms] OR "prevalance"[All Fields] OR "prevalences"[All Fields] OR "prevalence s"[All Fields] OR "prevalent"[All Fields] OR "prevalently"[All Fields] OR "prevalents"[All Fields] OR ("risk factors"[MeSH Terms] OR ("risk"[All Fields] AND "factors"[All Fields]) OR "risk factors"[All Fields]) OR ("risk assessment"[MeSH Terms] OR ("risk"[All Fields] AND "assessment"[All Fields]) OR "risk assessment"[All Fields]) OR ("epidemiologies"[All Fields] OR "epidemiology"[MeSH Subheading] OR "epidemiology"[All Fields] OR "epidemiology"[MeSH Terms] OR "epidemiology s"[All Fields]) OR ("mortality"[MeSH Terms] OR "mortality"[All Fields] OR "mortalities"[All Fields] OR "mortality"[MeSH Subheading])) AND ("coronary artery disease"[MeSH Terms] OR ("coronary"[All Fields] AND "artery"[All Fields] AND "disease"[All Fields]) OR "coronary artery disease"[All Fields] OR ("cardiovascular diseases"[MeSH Terms] OR ("cardiovascular"[All Fields] AND "diseases"[All Fields]) OR "cardiovascular diseases"[All Fields]) OR ("acute coronary syndrome"[MeSH Terms] OR ("acute"[All Fields] AND "coronary"[All Fields] AND "syndrome"[All Fields]) OR "acute coronary syndrome"[All Fields]) OR ("chronic disease"[MeSH Terms] OR ("chronic"[All Fields] AND "disease"[All Fields]) OR "chronic disease"[All Fields]) OR ("disease attributes"[MeSH Terms] OR ("disease"[All Fields] AND "attributes"[All Fields]) OR "disease attributes"[All Fields]) OR ("respiratory distress syndrome"[MeSH Terms] OR ("respiratory"[All Fields] AND "distress"[All Fields] AND "syndrome"[All Fields]) OR "respiratory distress syndrome"[All Fields]) OR ("respiratory distress syndrome"[MeSH Terms] OR ("respiratory"[All Fields] AND "distress"[All Fields] AND "syndrome"[All Fields]) OR "respiratory distress syndrome"[All Fields] OR "ards"[All Fields])) | 5,86,809 |
| 10 | ((SARS-CoV-2 OR COVID-19 OR Coronavirus Infections OR 2019-nCoV OR coronavirus disease 2019 OR Hospitalization) AND (Prevalence OR Risk Factors OR Risk Assessment OR Epidemiology OR Mortality)) AND (Coronary Artery Disease OR Cardiovascular Diseases OR Acute Coronary Syndrome OR Chronic Disease OR Disease Attributes OR Respiratory Distress Syndrome OR ARDS) NOT animal |  | (("sars cov 2"[MeSH Terms] OR "sars cov 2"[All Fields] OR "sars cov 2"[All Fields] OR ("covid 19"[All Fields] OR "covid 19"[MeSH Terms] OR "covid 19 vaccines"[All Fields] OR "covid 19 vaccines"[MeSH Terms] OR "covid 19 serotherapy"[All Fields] OR "covid 19 serotherapy"[Supplementary Concept] OR "covid 19 nucleic acid testing"[All Fields] OR "covid 19 nucleic acid testing"[MeSH Terms] OR "covid 19 serological testing"[All Fields] OR "covid 19 serological testing"[MeSH Terms] OR "covid 19 testing"[All Fields] OR "covid 19 testing"[MeSH Terms] OR "sars cov 2"[All Fields] OR "sars cov 2"[MeSH Terms] OR "severe acute respiratory syndrome coronavirus 2"[All Fields] OR "ncov"[All Fields] OR "2019 ncov"[All Fields] OR (("coronavirus"[MeSH Terms] OR "coronavirus"[All Fields] OR "cov"[All Fields]) AND 2019/11/01:3000/12/31[Date - Publication])) OR ("coronavirus infections"[MeSH Terms] OR ("coronavirus"[All Fields] AND "infections"[All Fields]) OR "coronavirus infections"[All Fields]) OR ("sars cov 2"[MeSH Terms] OR "sars cov 2"[All Fields] OR "2019 ncov"[All Fields]) OR ("covid 19"[MeSH Terms] OR "covid 19"[All Fields] OR "coronavirus disease 2019"[All Fields]) OR ("hospital s"[All Fields] OR "hospitalisation"[All Fields] OR "hospitalization"[MeSH Terms] OR "hospitalization"[All Fields] OR "hospitalising"[All Fields] OR "hospitality"[All Fields] OR "hospitalisations"[All Fields] OR "hospitalised"[All Fields] OR "hospitalizations"[All Fields] OR "hospitalized"[All Fields] OR "hospitalize"[All Fields] OR "hospitalizing"[All Fields] OR "hospitals"[MeSH Terms] OR "hospitals"[All Fields] OR "hospital"[All Fields])) AND ("epidemiology"[MeSH Subheading] OR "epidemiology"[All Fields] OR "prevalence"[All Fields] OR "prevalence"[MeSH Terms] OR "prevalance"[All Fields] OR "prevalences"[All Fields] OR "prevalence s"[All Fields] OR "prevalent"[All Fields] OR "prevalently"[All Fields] OR "prevalents"[All Fields] OR ("risk factors"[MeSH Terms] OR ("risk"[All Fields] AND "factors"[All Fields]) OR "risk factors"[All Fields]) OR ("risk assessment"[MeSH Terms] OR ("risk"[All Fields] AND "assessment"[All Fields]) OR "risk assessment"[All Fields]) OR ("epidemiologies"[All Fields] OR "epidemiology"[MeSH Subheading] OR "epidemiology"[All Fields] OR "epidemiology"[MeSH Terms] OR "epidemiology s"[All Fields]) OR ("mortality"[MeSH Terms] OR "mortality"[All Fields] OR "mortalities"[All Fields] OR "mortality"[MeSH Subheading])) AND ("coronary artery disease"[MeSH Terms] OR ("coronary"[All Fields] AND "artery"[All Fields] AND "disease"[All Fields]) OR "coronary artery disease"[All Fields] OR ("cardiovascular diseases"[MeSH Terms] OR ("cardiovascular"[All Fields] AND "diseases"[All Fields]) OR "cardiovascular diseases"[All Fields]) OR ("acute coronary syndrome"[MeSH Terms] OR ("acute"[All Fields] AND "coronary"[All Fields] AND "syndrome"[All Fields]) OR "acute coronary syndrome"[All Fields]) OR ("chronic disease"[MeSH Terms] OR ("chronic"[All Fields] AND "disease"[All Fields]) OR "chronic disease"[All Fields]) OR ("disease attributes"[MeSH Terms] OR ("disease"[All Fields] AND "attributes"[All Fields]) OR "disease attributes"[All Fields]) OR ("respiratory distress syndrome"[MeSH Terms] OR ("respiratory"[All Fields] AND "distress"[All Fields] AND "syndrome"[All Fields]) OR "respiratory distress syndrome"[All Fields]) OR ("respiratory distress syndrome"[MeSH Terms] OR ("respiratory"[All Fields] AND "distress"[All Fields] AND "syndrome"[All Fields]) OR "respiratory distress syndrome"[All Fields] OR "ards"[All Fields]))) NOT ("animals"[MeSH Terms:noexp] OR "animal"[All Fields]) | 5,63,545 |
| 11 | ((SARS-CoV-2 OR COVID-19 OR Coronavirus Infections OR 2019-nCoV OR coronavirus disease 2019 OR Hospitalization) AND (Prevalence OR Risk Factors OR Risk Assessment OR Epidemiology OR Mortality)) AND (Coronary Artery Disease OR Cardiovascular Diseases OR Acute Coronary Syndrome OR Chronic Disease OR Disease Attributes OR Respiratory Distress Syndrome OR ARDS) NOT animal | from 2019 - 2022 | ((("sars cov 2"[MeSH Terms] OR "sars cov 2"[All Fields] OR "sars cov 2"[All Fields] OR ("covid 19"[All Fields] OR "covid 19"[MeSH Terms] OR "covid 19 vaccines"[All Fields] OR "covid 19 vaccines"[MeSH Terms] OR "covid 19 serotherapy"[All Fields] OR "covid 19 serotherapy"[Supplementary Concept] OR "covid 19 nucleic acid testing"[All Fields] OR "covid 19 nucleic acid testing"[MeSH Terms] OR "covid 19 serological testing"[All Fields] OR "covid 19 serological testing"[MeSH Terms] OR "covid 19 testing"[All Fields] OR "covid 19 testing"[MeSH Terms] OR "sars cov 2"[All Fields] OR "sars cov 2"[MeSH Terms] OR "severe acute respiratory syndrome coronavirus 2"[All Fields] OR "ncov"[All Fields] OR "2019 ncov"[All Fields] OR (("coronavirus"[MeSH Terms] OR "coronavirus"[All Fields] OR "cov"[All Fields]) AND 2019/11/01:3000/12/31[Date - Publication])) OR ("coronavirus infections"[MeSH Terms] OR ("coronavirus"[All Fields] AND "infections"[All Fields]) OR "coronavirus infections"[All Fields]) OR ("sars cov 2"[MeSH Terms] OR "sars cov 2"[All Fields] OR "2019 ncov"[All Fields]) OR ("covid 19"[MeSH Terms] OR "covid 19"[All Fields] OR "coronavirus disease 2019"[All Fields]) OR ("hospital s"[All Fields] OR "hospitalisation"[All Fields] OR "hospitalization"[MeSH Terms] OR "hospitalization"[All Fields] OR "hospitalising"[All Fields] OR "hospitality"[All Fields] OR "hospitalisations"[All Fields] OR "hospitalised"[All Fields] OR "hospitalizations"[All Fields] OR "hospitalized"[All Fields] OR "hospitalize"[All Fields] OR "hospitalizing"[All Fields] OR "hospitals"[MeSH Terms] OR "hospitals"[All Fields] OR "hospital"[All Fields])) AND ("epidemiology"[MeSH Subheading] OR "epidemiology"[All Fields] OR "prevalence"[All Fields] OR "prevalence"[MeSH Terms] OR "prevalance"[All Fields] OR "prevalences"[All Fields] OR "prevalence s"[All Fields] OR "prevalent"[All Fields] OR "prevalently"[All Fields] OR "prevalents"[All Fields] OR ("risk factors"[MeSH Terms] OR ("risk"[All Fields] AND "factors"[All Fields]) OR "risk factors"[All Fields]) OR ("risk assessment"[MeSH Terms] OR ("risk"[All Fields] AND "assessment"[All Fields]) OR "risk assessment"[All Fields]) OR ("epidemiologies"[All Fields] OR "epidemiology"[MeSH Subheading] OR "epidemiology"[All Fields] OR "epidemiology"[MeSH Terms] OR "epidemiology s"[All Fields]) OR ("mortality"[MeSH Terms] OR "mortality"[All Fields] OR "mortalities"[All Fields] OR "mortality"[MeSH Subheading])) AND ("coronary artery disease"[MeSH Terms] OR ("coronary"[All Fields] AND "artery"[All Fields] AND "disease"[All Fields]) OR "coronary artery disease"[All Fields] OR ("cardiovascular diseases"[MeSH Terms] OR ("cardiovascular"[All Fields] AND "diseases"[All Fields]) OR "cardiovascular diseases"[All Fields]) OR ("acute coronary syndrome"[MeSH Terms] OR ("acute"[All Fields] AND "coronary"[All Fields] AND "syndrome"[All Fields]) OR "acute coronary syndrome"[All Fields]) OR ("chronic disease"[MeSH Terms] OR ("chronic"[All Fields] AND "disease"[All Fields]) OR "chronic disease"[All Fields]) OR ("disease attributes"[MeSH Terms] OR ("disease"[All Fields] AND "attributes"[All Fields]) OR "disease attributes"[All Fields]) OR ("respiratory distress syndrome"[MeSH Terms] OR ("respiratory"[All Fields] AND "distress"[All Fields] AND "syndrome"[All Fields]) OR "respiratory distress syndrome"[All Fields]) OR ("respiratory distress syndrome"[MeSH Terms] OR ("respiratory"[All Fields] AND "distress"[All Fields] AND "syndrome"[All Fields]) OR "respiratory distress syndrome"[All Fields] OR "ards"[All Fields]))) NOT ("animals"[MeSH Terms:noexp] OR "animal"[All Fields])) AND (2019:2022[pdat]) | 1,46,231 |
| 12 | ((SARS-CoV-2 OR COVID-19 OR Coronavirus Infections OR 2019-nCoV OR coronavirus disease 2019 OR Hospitalization) AND (Prevalence OR Risk Factors OR Risk Assessment OR Epidemiology OR Mortality)) AND (Coronary Artery Disease OR Cardiovascular Diseases OR Acute Coronary Syndrome OR Chronic Disease OR Disease Attributes OR Respiratory Distress Syndrome OR ARDS) NOT animal | English, from 2019 - 2022 | ((("sars cov 2"[MeSH Terms] OR "sars cov 2"[All Fields] OR "sars cov 2"[All Fields] OR ("covid 19"[All Fields] OR "covid 19"[MeSH Terms] OR "covid 19 vaccines"[All Fields] OR "covid 19 vaccines"[MeSH Terms] OR "covid 19 serotherapy"[All Fields] OR "covid 19 serotherapy"[Supplementary Concept] OR "covid 19 nucleic acid testing"[All Fields] OR "covid 19 nucleic acid testing"[MeSH Terms] OR "covid 19 serological testing"[All Fields] OR "covid 19 serological testing"[MeSH Terms] OR "covid 19 testing"[All Fields] OR "covid 19 testing"[MeSH Terms] OR "sars cov 2"[All Fields] OR "sars cov 2"[MeSH Terms] OR "severe acute respiratory syndrome coronavirus 2"[All Fields] OR "ncov"[All Fields] OR "2019 ncov"[All Fields] OR (("coronavirus"[MeSH Terms] OR "coronavirus"[All Fields] OR "cov"[All Fields]) AND 2019/11/01:3000/12/31[Date - Publication])) OR ("coronavirus infections"[MeSH Terms] OR ("coronavirus"[All Fields] AND "infections"[All Fields]) OR "coronavirus infections"[All Fields]) OR ("sars cov 2"[MeSH Terms] OR "sars cov 2"[All Fields] OR "2019 ncov"[All Fields]) OR ("covid 19"[MeSH Terms] OR "covid 19"[All Fields] OR "coronavirus disease 2019"[All Fields]) OR ("hospital s"[All Fields] OR "hospitalisation"[All Fields] OR "hospitalization"[MeSH Terms] OR "hospitalization"[All Fields] OR "hospitalising"[All Fields] OR "hospitality"[All Fields] OR "hospitalisations"[All Fields] OR "hospitalised"[All Fields] OR "hospitalizations"[All Fields] OR "hospitalized"[All Fields] OR "hospitalize"[All Fields] OR "hospitalizing"[All Fields] OR "hospitals"[MeSH Terms] OR "hospitals"[All Fields] OR "hospital"[All Fields])) AND ("epidemiology"[MeSH Subheading] OR "epidemiology"[All Fields] OR "prevalence"[All Fields] OR "prevalence"[MeSH Terms] OR "prevalance"[All Fields] OR "prevalences"[All Fields] OR "prevalence s"[All Fields] OR "prevalent"[All Fields] OR "prevalently"[All Fields] OR "prevalents"[All Fields] OR ("risk factors"[MeSH Terms] OR ("risk"[All Fields] AND "factors"[All Fields]) OR "risk factors"[All Fields]) OR ("risk assessment"[MeSH Terms] OR ("risk"[All Fields] AND "assessment"[All Fields]) OR "risk assessment"[All Fields]) OR ("epidemiologies"[All Fields] OR "epidemiology"[MeSH Subheading] OR "epidemiology"[All Fields] OR "epidemiology"[MeSH Terms] OR "epidemiology s"[All Fields]) OR ("mortality"[MeSH Terms] OR "mortality"[All Fields] OR "mortalities"[All Fields] OR "mortality"[MeSH Subheading])) AND ("coronary artery disease"[MeSH Terms] OR ("coronary"[All Fields] AND "artery"[All Fields] AND "disease"[All Fields]) OR "coronary artery disease"[All Fields] OR ("cardiovascular diseases"[MeSH Terms] OR ("cardiovascular"[All Fields] AND "diseases"[All Fields]) OR "cardiovascular diseases"[All Fields]) OR ("acute coronary syndrome"[MeSH Terms] OR ("acute"[All Fields] AND "coronary"[All Fields] AND "syndrome"[All Fields]) OR "acute coronary syndrome"[All Fields]) OR ("chronic disease"[MeSH Terms] OR ("chronic"[All Fields] AND "disease"[All Fields]) OR "chronic disease"[All Fields]) OR ("disease attributes"[MeSH Terms] OR ("disease"[All Fields] AND "attributes"[All Fields]) OR "disease attributes"[All Fields]) OR ("respiratory distress syndrome"[MeSH Terms] OR ("respiratory"[All Fields] AND "distress"[All Fields] AND "syndrome"[All Fields]) OR "respiratory distress syndrome"[All Fields]) OR ("respiratory distress syndrome"[MeSH Terms] OR ("respiratory"[All Fields] AND "distress"[All Fields] AND "syndrome"[All Fields]) OR "respiratory distress syndrome"[All Fields] OR "ards"[All Fields]))) NOT ("animals"[MeSH Terms:noexp] OR "animal"[All Fields])) AND ((english[Filter]) AND (2019:2022[pdat])) | 1,43,026 |
| 13 | ((SARS-CoV-2 OR COVID-19 OR Coronavirus Infections OR 2019-nCoV OR coronavirus disease 2019 OR Hospitalization) AND (Prevalence OR Risk Factors OR Risk Assessment OR Epidemiology OR Mortality)) AND (Coronary Artery Disease OR Cardiovascular Diseases OR Acute Coronary Syndrome OR Chronic Disease OR Disease Attributes OR Respiratory Distress Syndrome OR ARDS) NOT animal | English, Humans, from 2019 - 2022 | ((("sars cov 2"[MeSH Terms] OR "sars cov 2"[All Fields] OR "sars cov 2"[All Fields] OR ("covid 19"[All Fields] OR "covid 19"[MeSH Terms] OR "covid 19 vaccines"[All Fields] OR "covid 19 vaccines"[MeSH Terms] OR "covid 19 serotherapy"[All Fields] OR "covid 19 serotherapy"[Supplementary Concept] OR "covid 19 nucleic acid testing"[All Fields] OR "covid 19 nucleic acid testing"[MeSH Terms] OR "covid 19 serological testing"[All Fields] OR "covid 19 serological testing"[MeSH Terms] OR "covid 19 testing"[All Fields] OR "covid 19 testing"[MeSH Terms] OR "sars cov 2"[All Fields] OR "sars cov 2"[MeSH Terms] OR "severe acute respiratory syndrome coronavirus 2"[All Fields] OR "ncov"[All Fields] OR "2019 ncov"[All Fields] OR (("coronavirus"[MeSH Terms] OR "coronavirus"[All Fields] OR "cov"[All Fields]) AND 2019/11/01:3000/12/31[Date - Publication])) OR ("coronavirus infections"[MeSH Terms] OR ("coronavirus"[All Fields] AND "infections"[All Fields]) OR "coronavirus infections"[All Fields]) OR ("sars cov 2"[MeSH Terms] OR "sars cov 2"[All Fields] OR "2019 ncov"[All Fields]) OR ("covid 19"[MeSH Terms] OR "covid 19"[All Fields] OR "coronavirus disease 2019"[All Fields]) OR ("hospital s"[All Fields] OR "hospitalisation"[All Fields] OR "hospitalization"[MeSH Terms] OR "hospitalization"[All Fields] OR "hospitalising"[All Fields] OR "hospitality"[All Fields] OR "hospitalisations"[All Fields] OR "hospitalised"[All Fields] OR "hospitalizations"[All Fields] OR "hospitalized"[All Fields] OR "hospitalize"[All Fields] OR "hospitalizing"[All Fields] OR "hospitals"[MeSH Terms] OR "hospitals"[All Fields] OR "hospital"[All Fields])) AND ("epidemiology"[MeSH Subheading] OR "epidemiology"[All Fields] OR "prevalence"[All Fields] OR "prevalence"[MeSH Terms] OR "prevalance"[All Fields] OR "prevalences"[All Fields] OR "prevalence s"[All Fields] OR "prevalent"[All Fields] OR "prevalently"[All Fields] OR "prevalents"[All Fields] OR ("risk factors"[MeSH Terms] OR ("risk"[All Fields] AND "factors"[All Fields]) OR "risk factors"[All Fields]) OR ("risk assessment"[MeSH Terms] OR ("risk"[All Fields] AND "assessment"[All Fields]) OR "risk assessment"[All Fields]) OR ("epidemiologies"[All Fields] OR "epidemiology"[MeSH Subheading] OR "epidemiology"[All Fields] OR "epidemiology"[MeSH Terms] OR "epidemiology s"[All Fields]) OR ("mortality"[MeSH Terms] OR "mortality"[All Fields] OR "mortalities"[All Fields] OR "mortality"[MeSH Subheading])) AND ("coronary artery disease"[MeSH Terms] OR ("coronary"[All Fields] AND "artery"[All Fields] AND "disease"[All Fields]) OR "coronary artery disease"[All Fields] OR ("cardiovascular diseases"[MeSH Terms] OR ("cardiovascular"[All Fields] AND "diseases"[All Fields]) OR "cardiovascular diseases"[All Fields]) OR ("acute coronary syndrome"[MeSH Terms] OR ("acute"[All Fields] AND "coronary"[All Fields] AND "syndrome"[All Fields]) OR "acute coronary syndrome"[All Fields]) OR ("chronic disease"[MeSH Terms] OR ("chronic"[All Fields] AND "disease"[All Fields]) OR "chronic disease"[All Fields]) OR ("disease attributes"[MeSH Terms] OR ("disease"[All Fields] AND "attributes"[All Fields]) OR "disease attributes"[All Fields]) OR ("respiratory distress syndrome"[MeSH Terms] OR ("respiratory"[All Fields] AND "distress"[All Fields] AND "syndrome"[All Fields]) OR "respiratory distress syndrome"[All Fields]) OR ("respiratory distress syndrome"[MeSH Terms] OR ("respiratory"[All Fields] AND "distress"[All Fields] AND "syndrome"[All Fields]) OR "respiratory distress syndrome"[All Fields] OR "ards"[All Fields]))) NOT ("animals"[MeSH Terms:noexp] OR "animal"[All Fields])) AND ((humans[Filter]) AND (english[Filter]) AND (2019:2022[pdat])) | 1,28,670 |
| 14 | ((SARS-CoV-2 OR COVID-19 OR Coronavirus Infections OR 2019-nCoV OR coronavirus disease 2019 OR Hospitalization) AND (Prevalence OR Risk Factors OR Risk Assessment OR Epidemiology OR Mortality)) AND (Coronary Artery Disease OR Cardiovascular Diseases OR Acute Coronary Syndrome OR Chronic Disease OR Disease Attributes OR Respiratory Distress Syndrome OR ARDS) NOT animal | Review, Humans, English, from 2019 - 2022 | ((("sars cov 2"[MeSH Terms] OR "sars cov 2"[All Fields] OR "sars cov 2"[All Fields] OR ("covid 19"[All Fields] OR "covid 19"[MeSH Terms] OR "covid 19 vaccines"[All Fields] OR "covid 19 vaccines"[MeSH Terms] OR "covid 19 serotherapy"[All Fields] OR "covid 19 serotherapy"[Supplementary Concept] OR "covid 19 nucleic acid testing"[All Fields] OR "covid 19 nucleic acid testing"[MeSH Terms] OR "covid 19 serological testing"[All Fields] OR "covid 19 serological testing"[MeSH Terms] OR "covid 19 testing"[All Fields] OR "covid 19 testing"[MeSH Terms] OR "sars cov 2"[All Fields] OR "sars cov 2"[MeSH Terms] OR "severe acute respiratory syndrome coronavirus 2"[All Fields] OR "ncov"[All Fields] OR "2019 ncov"[All Fields] OR (("coronavirus"[MeSH Terms] OR "coronavirus"[All Fields] OR "cov"[All Fields]) AND 2019/11/01:3000/12/31[Date - Publication])) OR ("coronavirus infections"[MeSH Terms] OR ("coronavirus"[All Fields] AND "infections"[All Fields]) OR "coronavirus infections"[All Fields]) OR ("sars cov 2"[MeSH Terms] OR "sars cov 2"[All Fields] OR "2019 ncov"[All Fields]) OR ("covid 19"[MeSH Terms] OR "covid 19"[All Fields] OR "coronavirus disease 2019"[All Fields]) OR ("hospital s"[All Fields] OR "hospitalisation"[All Fields] OR "hospitalization"[MeSH Terms] OR "hospitalization"[All Fields] OR "hospitalising"[All Fields] OR "hospitality"[All Fields] OR "hospitalisations"[All Fields] OR "hospitalised"[All Fields] OR "hospitalizations"[All Fields] OR "hospitalized"[All Fields] OR "hospitalize"[All Fields] OR "hospitalizing"[All Fields] OR "hospitals"[MeSH Terms] OR "hospitals"[All Fields] OR "hospital"[All Fields])) AND ("epidemiology"[MeSH Subheading] OR "epidemiology"[All Fields] OR "prevalence"[All Fields] OR "prevalence"[MeSH Terms] OR "prevalance"[All Fields] OR "prevalences"[All Fields] OR "prevalence s"[All Fields] OR "prevalent"[All Fields] OR "prevalently"[All Fields] OR "prevalents"[All Fields] OR ("risk factors"[MeSH Terms] OR ("risk"[All Fields] AND "factors"[All Fields]) OR "risk factors"[All Fields]) OR ("risk assessment"[MeSH Terms] OR ("risk"[All Fields] AND "assessment"[All Fields]) OR "risk assessment"[All Fields]) OR ("epidemiologies"[All Fields] OR "epidemiology"[MeSH Subheading] OR "epidemiology"[All Fields] OR "epidemiology"[MeSH Terms] OR "epidemiology s"[All Fields]) OR ("mortality"[MeSH Terms] OR "mortality"[All Fields] OR "mortalities"[All Fields] OR "mortality"[MeSH Subheading])) AND ("coronary artery disease"[MeSH Terms] OR ("coronary"[All Fields] AND "artery"[All Fields] AND "disease"[All Fields]) OR "coronary artery disease"[All Fields] OR ("cardiovascular diseases"[MeSH Terms] OR ("cardiovascular"[All Fields] AND "diseases"[All Fields]) OR "cardiovascular diseases"[All Fields]) OR ("acute coronary syndrome"[MeSH Terms] OR ("acute"[All Fields] AND "coronary"[All Fields] AND "syndrome"[All Fields]) OR "acute coronary syndrome"[All Fields]) OR ("chronic disease"[MeSH Terms] OR ("chronic"[All Fields] AND "disease"[All Fields]) OR "chronic disease"[All Fields]) OR ("disease attributes"[MeSH Terms] OR ("disease"[All Fields] AND "attributes"[All Fields]) OR "disease attributes"[All Fields]) OR ("respiratory distress syndrome"[MeSH Terms] OR ("respiratory"[All Fields] AND "distress"[All Fields] AND "syndrome"[All Fields]) OR "respiratory distress syndrome"[All Fields]) OR ("respiratory distress syndrome"[MeSH Terms] OR ("respiratory"[All Fields] AND "distress"[All Fields] AND "syndrome"[All Fields]) OR "respiratory distress syndrome"[All Fields] OR "ards"[All Fields]))) NOT ("animals"[MeSH Terms:noexp] OR "animal"[All Fields])) AND ((review[Filter]) AND (humans[Filter]) AND (english[Filter]) AND (2019:2022[pdat])) | 12,610 |
| 15 | ((SARS-CoV-2 OR COVID-19 OR Coronavirus Infections OR 2019-nCoV OR coronavirus disease 2019 OR Hospitalization) AND (Prevalence OR Risk Factors OR Risk Assessment OR Epidemiology OR Mortality)) AND (Coronary Artery Disease OR Cardiovascular Diseases OR Acute Coronary Syndrome OR Chronic Disease OR Disease Attributes OR Respiratory Distress Syndrome OR ARDS) NOT animal | Humans, English, from 2019 - 2022 | ((("sars cov 2"[MeSH Terms] OR "sars cov 2"[All Fields] OR "sars cov 2"[All Fields] OR ("covid 19"[All Fields] OR "covid 19"[MeSH Terms] OR "covid 19 vaccines"[All Fields] OR "covid 19 vaccines"[MeSH Terms] OR "covid 19 serotherapy"[All Fields] OR "covid 19 serotherapy"[Supplementary Concept] OR "covid 19 nucleic acid testing"[All Fields] OR "covid 19 nucleic acid testing"[MeSH Terms] OR "covid 19 serological testing"[All Fields] OR "covid 19 serological testing"[MeSH Terms] OR "covid 19 testing"[All Fields] OR "covid 19 testing"[MeSH Terms] OR "sars cov 2"[All Fields] OR "sars cov 2"[MeSH Terms] OR "severe acute respiratory syndrome coronavirus 2"[All Fields] OR "ncov"[All Fields] OR "2019 ncov"[All Fields] OR (("coronavirus"[MeSH Terms] OR "coronavirus"[All Fields] OR "cov"[All Fields]) AND 2019/11/01:3000/12/31[Date - Publication])) OR ("coronavirus infections"[MeSH Terms] OR ("coronavirus"[All Fields] AND "infections"[All Fields]) OR "coronavirus infections"[All Fields]) OR ("sars cov 2"[MeSH Terms] OR "sars cov 2"[All Fields] OR "2019 ncov"[All Fields]) OR ("covid 19"[MeSH Terms] OR "covid 19"[All Fields] OR "coronavirus disease 2019"[All Fields]) OR ("hospital s"[All Fields] OR "hospitalisation"[All Fields] OR "hospitalization"[MeSH Terms] OR "hospitalization"[All Fields] OR "hospitalising"[All Fields] OR "hospitality"[All Fields] OR "hospitalisations"[All Fields] OR "hospitalised"[All Fields] OR "hospitalizations"[All Fields] OR "hospitalized"[All Fields] OR "hospitalize"[All Fields] OR "hospitalizing"[All Fields] OR "hospitals"[MeSH Terms] OR "hospitals"[All Fields] OR "hospital"[All Fields])) AND ("epidemiology"[MeSH Subheading] OR "epidemiology"[All Fields] OR "prevalence"[All Fields] OR "prevalence"[MeSH Terms] OR "prevalance"[All Fields] OR "prevalences"[All Fields] OR "prevalence s"[All Fields] OR "prevalent"[All Fields] OR "prevalently"[All Fields] OR "prevalents"[All Fields] OR ("risk factors"[MeSH Terms] OR ("risk"[All Fields] AND "factors"[All Fields]) OR "risk factors"[All Fields]) OR ("risk assessment"[MeSH Terms] OR ("risk"[All Fields] AND "assessment"[All Fields]) OR "risk assessment"[All Fields]) OR ("epidemiologies"[All Fields] OR "epidemiology"[MeSH Subheading] OR "epidemiology"[All Fields] OR "epidemiology"[MeSH Terms] OR "epidemiology s"[All Fields]) OR ("mortality"[MeSH Terms] OR "mortality"[All Fields] OR "mortalities"[All Fields] OR "mortality"[MeSH Subheading])) AND ("coronary artery disease"[MeSH Terms] OR ("coronary"[All Fields] AND "artery"[All Fields] AND "disease"[All Fields]) OR "coronary artery disease"[All Fields] OR ("cardiovascular diseases"[MeSH Terms] OR ("cardiovascular"[All Fields] AND "diseases"[All Fields]) OR "cardiovascular diseases"[All Fields]) OR ("acute coronary syndrome"[MeSH Terms] OR ("acute"[All Fields] AND "coronary"[All Fields] AND "syndrome"[All Fields]) OR "acute coronary syndrome"[All Fields]) OR ("chronic disease"[MeSH Terms] OR ("chronic"[All Fields] AND "disease"[All Fields]) OR "chronic disease"[All Fields]) OR ("disease attributes"[MeSH Terms] OR ("disease"[All Fields] AND "attributes"[All Fields]) OR "disease attributes"[All Fields]) OR ("respiratory distress syndrome"[MeSH Terms] OR ("respiratory"[All Fields] AND "distress"[All Fields] AND "syndrome"[All Fields]) OR "respiratory distress syndrome"[All Fields]) OR ("respiratory distress syndrome"[MeSH Terms] OR ("respiratory"[All Fields] AND "distress"[All Fields] AND "syndrome"[All Fields]) OR "respiratory distress syndrome"[All Fields] OR "ards"[All Fields]))) NOT ("animals"[MeSH Terms:noexp] OR "animal"[All Fields])) AND ((humans[Filter]) AND (english[Filter]) AND (2019:2022[pdat])) | 1,28,670 |
| 16 | ((SARS-CoV-2 OR COVID-19 OR Coronavirus Infections OR 2019-nCoV OR coronavirus disease 2019 OR Hospitalization) AND (Prevalence OR Risk Factors OR Risk Assessment OR Epidemiology OR Mortality)) AND (Coronary Artery Disease OR Cardiovascular Diseases OR Acute Coronary Syndrome OR Chronic Disease OR Disease Attributes OR Respiratory Distress Syndrome OR ARDS) NOT animal | Books and Documents, Humans, English, from 2019 - 2022 | ((("sars cov 2"[MeSH Terms] OR "sars cov 2"[All Fields] OR "sars cov 2"[All Fields] OR ("covid 19"[All Fields] OR "covid 19"[MeSH Terms] OR "covid 19 vaccines"[All Fields] OR "covid 19 vaccines"[MeSH Terms] OR "covid 19 serotherapy"[All Fields] OR "covid 19 serotherapy"[Supplementary Concept] OR "covid 19 nucleic acid testing"[All Fields] OR "covid 19 nucleic acid testing"[MeSH Terms] OR "covid 19 serological testing"[All Fields] OR "covid 19 serological testing"[MeSH Terms] OR "covid 19 testing"[All Fields] OR "covid 19 testing"[MeSH Terms] OR "sars cov 2"[All Fields] OR "sars cov 2"[MeSH Terms] OR "severe acute respiratory syndrome coronavirus 2"[All Fields] OR "ncov"[All Fields] OR "2019 ncov"[All Fields] OR (("coronavirus"[MeSH Terms] OR "coronavirus"[All Fields] OR "cov"[All Fields]) AND 2019/11/01:3000/12/31[Date - Publication])) OR ("coronavirus infections"[MeSH Terms] OR ("coronavirus"[All Fields] AND "infections"[All Fields]) OR "coronavirus infections"[All Fields]) OR ("sars cov 2"[MeSH Terms] OR "sars cov 2"[All Fields] OR "2019 ncov"[All Fields]) OR ("covid 19"[MeSH Terms] OR "covid 19"[All Fields] OR "coronavirus disease 2019"[All Fields]) OR ("hospital s"[All Fields] OR "hospitalisation"[All Fields] OR "hospitalization"[MeSH Terms] OR "hospitalization"[All Fields] OR "hospitalising"[All Fields] OR "hospitality"[All Fields] OR "hospitalisations"[All Fields] OR "hospitalised"[All Fields] OR "hospitalizations"[All Fields] OR "hospitalized"[All Fields] OR "hospitalize"[All Fields] OR "hospitalizing"[All Fields] OR "hospitals"[MeSH Terms] OR "hospitals"[All Fields] OR "hospital"[All Fields])) AND ("epidemiology"[MeSH Subheading] OR "epidemiology"[All Fields] OR "prevalence"[All Fields] OR "prevalence"[MeSH Terms] OR "prevalance"[All Fields] OR "prevalences"[All Fields] OR "prevalence s"[All Fields] OR "prevalent"[All Fields] OR "prevalently"[All Fields] OR "prevalents"[All Fields] OR ("risk factors"[MeSH Terms] OR ("risk"[All Fields] AND "factors"[All Fields]) OR "risk factors"[All Fields]) OR ("risk assessment"[MeSH Terms] OR ("risk"[All Fields] AND "assessment"[All Fields]) OR "risk assessment"[All Fields]) OR ("epidemiologies"[All Fields] OR "epidemiology"[MeSH Subheading] OR "epidemiology"[All Fields] OR "epidemiology"[MeSH Terms] OR "epidemiology s"[All Fields]) OR ("mortality"[MeSH Terms] OR "mortality"[All Fields] OR "mortalities"[All Fields] OR "mortality"[MeSH Subheading])) AND ("coronary artery disease"[MeSH Terms] OR ("coronary"[All Fields] AND "artery"[All Fields] AND "disease"[All Fields]) OR "coronary artery disease"[All Fields] OR ("cardiovascular diseases"[MeSH Terms] OR ("cardiovascular"[All Fields] AND "diseases"[All Fields]) OR "cardiovascular diseases"[All Fields]) OR ("acute coronary syndrome"[MeSH Terms] OR ("acute"[All Fields] AND "coronary"[All Fields] AND "syndrome"[All Fields]) OR "acute coronary syndrome"[All Fields]) OR ("chronic disease"[MeSH Terms] OR ("chronic"[All Fields] AND "disease"[All Fields]) OR "chronic disease"[All Fields]) OR ("disease attributes"[MeSH Terms] OR ("disease"[All Fields] AND "attributes"[All Fields]) OR "disease attributes"[All Fields]) OR ("respiratory distress syndrome"[MeSH Terms] OR ("respiratory"[All Fields] AND "distress"[All Fields] AND "syndrome"[All Fields]) OR "respiratory distress syndrome"[All Fields]) OR ("respiratory distress syndrome"[MeSH Terms] OR ("respiratory"[All Fields] AND "distress"[All Fields] AND "syndrome"[All Fields]) OR "respiratory distress syndrome"[All Fields] OR "ards"[All Fields]))) NOT ("animals"[MeSH Terms:noexp] OR "animal"[All Fields])) AND ((booksdocs[Filter]) AND (humans[Filter]) AND (english[Filter]) AND (2019:2022[pdat])) | 24 |
| 17 | ((SARS-CoV-2 OR COVID-19 OR Coronavirus Infections OR 2019-nCoV OR coronavirus disease 2019 OR Hospitalization) AND (Prevalence OR Risk Factors OR Risk Assessment OR Epidemiology OR Mortality)) AND (Coronary Artery Disease OR Cardiovascular Diseases OR Acute Coronary Syndrome OR Chronic Disease OR Disease Attributes OR Respiratory Distress Syndrome OR ARDS) NOT animal | Books and Documents, Clinical Trial, Humans, English, from 2019 - 2022 | ((("sars cov 2"[MeSH Terms] OR "sars cov 2"[All Fields] OR "sars cov 2"[All Fields] OR ("covid 19"[All Fields] OR "covid 19"[MeSH Terms] OR "covid 19 vaccines"[All Fields] OR "covid 19 vaccines"[MeSH Terms] OR "covid 19 serotherapy"[All Fields] OR "covid 19 serotherapy"[Supplementary Concept] OR "covid 19 nucleic acid testing"[All Fields] OR "covid 19 nucleic acid testing"[MeSH Terms] OR "covid 19 serological testing"[All Fields] OR "covid 19 serological testing"[MeSH Terms] OR "covid 19 testing"[All Fields] OR "covid 19 testing"[MeSH Terms] OR "sars cov 2"[All Fields] OR "sars cov 2"[MeSH Terms] OR "severe acute respiratory syndrome coronavirus 2"[All Fields] OR "ncov"[All Fields] OR "2019 ncov"[All Fields] OR (("coronavirus"[MeSH Terms] OR "coronavirus"[All Fields] OR "cov"[All Fields]) AND 2019/11/01:3000/12/31[Date - Publication])) OR ("coronavirus infections"[MeSH Terms] OR ("coronavirus"[All Fields] AND "infections"[All Fields]) OR "coronavirus infections"[All Fields]) OR ("sars cov 2"[MeSH Terms] OR "sars cov 2"[All Fields] OR "2019 ncov"[All Fields]) OR ("covid 19"[MeSH Terms] OR "covid 19"[All Fields] OR "coronavirus disease 2019"[All Fields]) OR ("hospital s"[All Fields] OR "hospitalisation"[All Fields] OR "hospitalization"[MeSH Terms] OR "hospitalization"[All Fields] OR "hospitalising"[All Fields] OR "hospitality"[All Fields] OR "hospitalisations"[All Fields] OR "hospitalised"[All Fields] OR "hospitalizations"[All Fields] OR "hospitalized"[All Fields] OR "hospitalize"[All Fields] OR "hospitalizing"[All Fields] OR "hospitals"[MeSH Terms] OR "hospitals"[All Fields] OR "hospital"[All Fields])) AND ("epidemiology"[MeSH Subheading] OR "epidemiology"[All Fields] OR "prevalence"[All Fields] OR "prevalence"[MeSH Terms] OR "prevalance"[All Fields] OR "prevalences"[All Fields] OR "prevalence s"[All Fields] OR "prevalent"[All Fields] OR "prevalently"[All Fields] OR "prevalents"[All Fields] OR ("risk factors"[MeSH Terms] OR ("risk"[All Fields] AND "factors"[All Fields]) OR "risk factors"[All Fields]) OR ("risk assessment"[MeSH Terms] OR ("risk"[All Fields] AND "assessment"[All Fields]) OR "risk assessment"[All Fields]) OR ("epidemiologies"[All Fields] OR "epidemiology"[MeSH Subheading] OR "epidemiology"[All Fields] OR "epidemiology"[MeSH Terms] OR "epidemiology s"[All Fields]) OR ("mortality"[MeSH Terms] OR "mortality"[All Fields] OR "mortalities"[All Fields] OR "mortality"[MeSH Subheading])) AND ("coronary artery disease"[MeSH Terms] OR ("coronary"[All Fields] AND "artery"[All Fields] AND "disease"[All Fields]) OR "coronary artery disease"[All Fields] OR ("cardiovascular diseases"[MeSH Terms] OR ("cardiovascular"[All Fields] AND "diseases"[All Fields]) OR "cardiovascular diseases"[All Fields]) OR ("acute coronary syndrome"[MeSH Terms] OR ("acute"[All Fields] AND "coronary"[All Fields] AND "syndrome"[All Fields]) OR "acute coronary syndrome"[All Fields]) OR ("chronic disease"[MeSH Terms] OR ("chronic"[All Fields] AND "disease"[All Fields]) OR "chronic disease"[All Fields]) OR ("disease attributes"[MeSH Terms] OR ("disease"[All Fields] AND "attributes"[All Fields]) OR "disease attributes"[All Fields]) OR ("respiratory distress syndrome"[MeSH Terms] OR ("respiratory"[All Fields] AND "distress"[All Fields] AND "syndrome"[All Fields]) OR "respiratory distress syndrome"[All Fields]) OR ("respiratory distress syndrome"[MeSH Terms] OR ("respiratory"[All Fields] AND "distress"[All Fields] AND "syndrome"[All Fields]) OR "respiratory distress syndrome"[All Fields] OR "ards"[All Fields]))) NOT ("animals"[MeSH Terms:noexp] OR "animal"[All Fields])) AND ((booksdocs[Filter] OR clinicaltrial[Filter]) AND (humans[Filter]) AND (english[Filter]) AND (2019:2022[pdat])) | 6,409 |
| 18 | ((SARS-CoV-2 OR COVID-19 OR Coronavirus Infections OR 2019-nCoV OR coronavirus disease 2019 OR Hospitalization) AND (Prevalence OR Risk Factors OR Risk Assessment OR Epidemiology OR Mortality)) AND (Coronary Artery Disease OR Cardiovascular Diseases OR Acute Coronary Syndrome OR Chronic Disease OR Disease Attributes OR Respiratory Distress Syndrome OR ARDS) NOT animal | Clinical Trial, Humans, English, from 2019 - 2022 | ((("sars cov 2"[MeSH Terms] OR "sars cov 2"[All Fields] OR "sars cov 2"[All Fields] OR ("covid 19"[All Fields] OR "covid 19"[MeSH Terms] OR "covid 19 vaccines"[All Fields] OR "covid 19 vaccines"[MeSH Terms] OR "covid 19 serotherapy"[All Fields] OR "covid 19 serotherapy"[Supplementary Concept] OR "covid 19 nucleic acid testing"[All Fields] OR "covid 19 nucleic acid testing"[MeSH Terms] OR "covid 19 serological testing"[All Fields] OR "covid 19 serological testing"[MeSH Terms] OR "covid 19 testing"[All Fields] OR "covid 19 testing"[MeSH Terms] OR "sars cov 2"[All Fields] OR "sars cov 2"[MeSH Terms] OR "severe acute respiratory syndrome coronavirus 2"[All Fields] OR "ncov"[All Fields] OR "2019 ncov"[All Fields] OR (("coronavirus"[MeSH Terms] OR "coronavirus"[All Fields] OR "cov"[All Fields]) AND 2019/11/01:3000/12/31[Date - Publication])) OR ("coronavirus infections"[MeSH Terms] OR ("coronavirus"[All Fields] AND "infections"[All Fields]) OR "coronavirus infections"[All Fields]) OR ("sars cov 2"[MeSH Terms] OR "sars cov 2"[All Fields] OR "2019 ncov"[All Fields]) OR ("covid 19"[MeSH Terms] OR "covid 19"[All Fields] OR "coronavirus disease 2019"[All Fields]) OR ("hospital s"[All Fields] OR "hospitalisation"[All Fields] OR "hospitalization"[MeSH Terms] OR "hospitalization"[All Fields] OR "hospitalising"[All Fields] OR "hospitality"[All Fields] OR "hospitalisations"[All Fields] OR "hospitalised"[All Fields] OR "hospitalizations"[All Fields] OR "hospitalized"[All Fields] OR "hospitalize"[All Fields] OR "hospitalizing"[All Fields] OR "hospitals"[MeSH Terms] OR "hospitals"[All Fields] OR "hospital"[All Fields])) AND ("epidemiology"[MeSH Subheading] OR "epidemiology"[All Fields] OR "prevalence"[All Fields] OR "prevalence"[MeSH Terms] OR "prevalance"[All Fields] OR "prevalences"[All Fields] OR "prevalence s"[All Fields] OR "prevalent"[All Fields] OR "prevalently"[All Fields] OR "prevalents"[All Fields] OR ("risk factors"[MeSH Terms] OR ("risk"[All Fields] AND "factors"[All Fields]) OR "risk factors"[All Fields]) OR ("risk assessment"[MeSH Terms] OR ("risk"[All Fields] AND "assessment"[All Fields]) OR "risk assessment"[All Fields]) OR ("epidemiologies"[All Fields] OR "epidemiology"[MeSH Subheading] OR "epidemiology"[All Fields] OR "epidemiology"[MeSH Terms] OR "epidemiology s"[All Fields]) OR ("mortality"[MeSH Terms] OR "mortality"[All Fields] OR "mortalities"[All Fields] OR "mortality"[MeSH Subheading])) AND ("coronary artery disease"[MeSH Terms] OR ("coronary"[All Fields] AND "artery"[All Fields] AND "disease"[All Fields]) OR "coronary artery disease"[All Fields] OR ("cardiovascular diseases"[MeSH Terms] OR ("cardiovascular"[All Fields] AND "diseases"[All Fields]) OR "cardiovascular diseases"[All Fields]) OR ("acute coronary syndrome"[MeSH Terms] OR ("acute"[All Fields] AND "coronary"[All Fields] AND "syndrome"[All Fields]) OR "acute coronary syndrome"[All Fields]) OR ("chronic disease"[MeSH Terms] OR ("chronic"[All Fields] AND "disease"[All Fields]) OR "chronic disease"[All Fields]) OR ("disease attributes"[MeSH Terms] OR ("disease"[All Fields] AND "attributes"[All Fields]) OR "disease attributes"[All Fields]) OR ("respiratory distress syndrome"[MeSH Terms] OR ("respiratory"[All Fields] AND "distress"[All Fields] AND "syndrome"[All Fields]) OR "respiratory distress syndrome"[All Fields]) OR ("respiratory distress syndrome"[MeSH Terms] OR ("respiratory"[All Fields] AND "distress"[All Fields] AND "syndrome"[All Fields]) OR "respiratory distress syndrome"[All Fields] OR "ards"[All Fields]))) NOT ("animals"[MeSH Terms:noexp] OR "animal"[All Fields])) AND ((clinicaltrial[Filter]) AND (humans[Filter]) AND (english[Filter]) AND (2019:2022[pdat])) | 6,400 |
| 19 | ((SARS-CoV-2 OR COVID-19 OR Coronavirus Infections OR 2019-nCoV OR coronavirus disease 2019 OR Hospitalization) AND (Prevalence OR Risk Factors OR Risk Assessment OR Epidemiology OR Mortality)) AND (Coronary Artery Disease OR Cardiovascular Diseases OR Acute Coronary Syndrome OR Chronic Disease OR Disease Attributes OR Respiratory Distress Syndrome OR ARDS) NOT animal | Clinical Trial, Randomized Controlled Trial, Humans, English, from 2019 - 2022 | ((("sars cov 2"[MeSH Terms] OR "sars cov 2"[All Fields] OR "sars cov 2"[All Fields] OR ("covid 19"[All Fields] OR "covid 19"[MeSH Terms] OR "covid 19 vaccines"[All Fields] OR "covid 19 vaccines"[MeSH Terms] OR "covid 19 serotherapy"[All Fields] OR "covid 19 serotherapy"[Supplementary Concept] OR "covid 19 nucleic acid testing"[All Fields] OR "covid 19 nucleic acid testing"[MeSH Terms] OR "covid 19 serological testing"[All Fields] OR "covid 19 serological testing"[MeSH Terms] OR "covid 19 testing"[All Fields] OR "covid 19 testing"[MeSH Terms] OR "sars cov 2"[All Fields] OR "sars cov 2"[MeSH Terms] OR "severe acute respiratory syndrome coronavirus 2"[All Fields] OR "ncov"[All Fields] OR "2019 ncov"[All Fields] OR (("coronavirus"[MeSH Terms] OR "coronavirus"[All Fields] OR "cov"[All Fields]) AND 2019/11/01:3000/12/31[Date - Publication])) OR ("coronavirus infections"[MeSH Terms] OR ("coronavirus"[All Fields] AND "infections"[All Fields]) OR "coronavirus infections"[All Fields]) OR ("sars cov 2"[MeSH Terms] OR "sars cov 2"[All Fields] OR "2019 ncov"[All Fields]) OR ("covid 19"[MeSH Terms] OR "covid 19"[All Fields] OR "coronavirus disease 2019"[All Fields]) OR ("hospital s"[All Fields] OR "hospitalisation"[All Fields] OR "hospitalization"[MeSH Terms] OR "hospitalization"[All Fields] OR "hospitalising"[All Fields] OR "hospitality"[All Fields] OR "hospitalisations"[All Fields] OR "hospitalised"[All Fields] OR "hospitalizations"[All Fields] OR "hospitalized"[All Fields] OR "hospitalize"[All Fields] OR "hospitalizing"[All Fields] OR "hospitals"[MeSH Terms] OR "hospitals"[All Fields] OR "hospital"[All Fields])) AND ("epidemiology"[MeSH Subheading] OR "epidemiology"[All Fields] OR "prevalence"[All Fields] OR "prevalence"[MeSH Terms] OR "prevalance"[All Fields] OR "prevalences"[All Fields] OR "prevalence s"[All Fields] OR "prevalent"[All Fields] OR "prevalently"[All Fields] OR "prevalents"[All Fields] OR ("risk factors"[MeSH Terms] OR ("risk"[All Fields] AND "factors"[All Fields]) OR "risk factors"[All Fields]) OR ("risk assessment"[MeSH Terms] OR ("risk"[All Fields] AND "assessment"[All Fields]) OR "risk assessment"[All Fields]) OR ("epidemiologies"[All Fields] OR "epidemiology"[MeSH Subheading] OR "epidemiology"[All Fields] OR "epidemiology"[MeSH Terms] OR "epidemiology s"[All Fields]) OR ("mortality"[MeSH Terms] OR "mortality"[All Fields] OR "mortalities"[All Fields] OR "mortality"[MeSH Subheading])) AND ("coronary artery disease"[MeSH Terms] OR ("coronary"[All Fields] AND "artery"[All Fields] AND "disease"[All Fields]) OR "coronary artery disease"[All Fields] OR ("cardiovascular diseases"[MeSH Terms] OR ("cardiovascular"[All Fields] AND "diseases"[All Fields]) OR "cardiovascular diseases"[All Fields]) OR ("acute coronary syndrome"[MeSH Terms] OR ("acute"[All Fields] AND "coronary"[All Fields] AND "syndrome"[All Fields]) OR "acute coronary syndrome"[All Fields]) OR ("chronic disease"[MeSH Terms] OR ("chronic"[All Fields] AND "disease"[All Fields]) OR "chronic disease"[All Fields]) OR ("disease attributes"[MeSH Terms] OR ("disease"[All Fields] AND "attributes"[All Fields]) OR "disease attributes"[All Fields]) OR ("respiratory distress syndrome"[MeSH Terms] OR ("respiratory"[All Fields] AND "distress"[All Fields] AND "syndrome"[All Fields]) OR "respiratory distress syndrome"[All Fields]) OR ("respiratory distress syndrome"[MeSH Terms] OR ("respiratory"[All Fields] AND "distress"[All Fields] AND "syndrome"[All Fields]) OR "respiratory distress syndrome"[All Fields] OR "ards"[All Fields]))) NOT ("animals"[MeSH Terms:noexp] OR "animal"[All Fields])) AND ((clinicaltrial[Filter] OR randomizedcontrolledtrial[Filter]) AND (humans[Filter]) AND (english[Filter]) AND (2019:2022[pdat])) | 6,400 |
| 20 | ((SARS-CoV-2 OR COVID-19 OR Coronavirus Infections OR 2019-nCoV OR coronavirus disease 2019 OR Hospitalization) AND (Prevalence OR Risk Factors OR Risk Assessment OR Epidemiology OR Mortality)) AND (Coronary Artery Disease OR Cardiovascular Diseases OR Acute Coronary Syndrome OR Chronic Disease OR Disease Attributes OR Respiratory Distress Syndrome OR ARDS) NOT animal | Clinical Study, Clinical Trial, Randomized Controlled Trial, Humans, English, from 2019 - 2022 | ((("sars cov 2"[MeSH Terms] OR "sars cov 2"[All Fields] OR "sars cov 2"[All Fields] OR ("covid 19"[All Fields] OR "covid 19"[MeSH Terms] OR "covid 19 vaccines"[All Fields] OR "covid 19 vaccines"[MeSH Terms] OR "covid 19 serotherapy"[All Fields] OR "covid 19 serotherapy"[Supplementary Concept] OR "covid 19 nucleic acid testing"[All Fields] OR "covid 19 nucleic acid testing"[MeSH Terms] OR "covid 19 serological testing"[All Fields] OR "covid 19 serological testing"[MeSH Terms] OR "covid 19 testing"[All Fields] OR "covid 19 testing"[MeSH Terms] OR "sars cov 2"[All Fields] OR "sars cov 2"[MeSH Terms] OR "severe acute respiratory syndrome coronavirus 2"[All Fields] OR "ncov"[All Fields] OR "2019 ncov"[All Fields] OR (("coronavirus"[MeSH Terms] OR "coronavirus"[All Fields] OR "cov"[All Fields]) AND 2019/11/01:3000/12/31[Date - Publication])) OR ("coronavirus infections"[MeSH Terms] OR ("coronavirus"[All Fields] AND "infections"[All Fields]) OR "coronavirus infections"[All Fields]) OR ("sars cov 2"[MeSH Terms] OR "sars cov 2"[All Fields] OR "2019 ncov"[All Fields]) OR ("covid 19"[MeSH Terms] OR "covid 19"[All Fields] OR "coronavirus disease 2019"[All Fields]) OR ("hospital s"[All Fields] OR "hospitalisation"[All Fields] OR "hospitalization"[MeSH Terms] OR "hospitalization"[All Fields] OR "hospitalising"[All Fields] OR "hospitality"[All Fields] OR "hospitalisations"[All Fields] OR "hospitalised"[All Fields] OR "hospitalizations"[All Fields] OR "hospitalized"[All Fields] OR "hospitalize"[All Fields] OR "hospitalizing"[All Fields] OR "hospitals"[MeSH Terms] OR "hospitals"[All Fields] OR "hospital"[All Fields])) AND ("epidemiology"[MeSH Subheading] OR "epidemiology"[All Fields] OR "prevalence"[All Fields] OR "prevalence"[MeSH Terms] OR "prevalance"[All Fields] OR "prevalences"[All Fields] OR "prevalence s"[All Fields] OR "prevalent"[All Fields] OR "prevalently"[All Fields] OR "prevalents"[All Fields] OR ("risk factors"[MeSH Terms] OR ("risk"[All Fields] AND "factors"[All Fields]) OR "risk factors"[All Fields]) OR ("risk assessment"[MeSH Terms] OR ("risk"[All Fields] AND "assessment"[All Fields]) OR "risk assessment"[All Fields]) OR ("epidemiologies"[All Fields] OR "epidemiology"[MeSH Subheading] OR "epidemiology"[All Fields] OR "epidemiology"[MeSH Terms] OR "epidemiology s"[All Fields]) OR ("mortality"[MeSH Terms] OR "mortality"[All Fields] OR "mortalities"[All Fields] OR "mortality"[MeSH Subheading])) AND ("coronary artery disease"[MeSH Terms] OR ("coronary"[All Fields] AND "artery"[All Fields] AND "disease"[All Fields]) OR "coronary artery disease"[All Fields] OR ("cardiovascular diseases"[MeSH Terms] OR ("cardiovascular"[All Fields] AND "diseases"[All Fields]) OR "cardiovascular diseases"[All Fields]) OR ("acute coronary syndrome"[MeSH Terms] OR ("acute"[All Fields] AND "coronary"[All Fields] AND "syndrome"[All Fields]) OR "acute coronary syndrome"[All Fields]) OR ("chronic disease"[MeSH Terms] OR ("chronic"[All Fields] AND "disease"[All Fields]) OR "chronic disease"[All Fields]) OR ("disease attributes"[MeSH Terms] OR ("disease"[All Fields] AND "attributes"[All Fields]) OR "disease attributes"[All Fields]) OR ("respiratory distress syndrome"[MeSH Terms] OR ("respiratory"[All Fields] AND "distress"[All Fields] AND "syndrome"[All Fields]) OR "respiratory distress syndrome"[All Fields]) OR ("respiratory distress syndrome"[MeSH Terms] OR ("respiratory"[All Fields] AND "distress"[All Fields] AND "syndrome"[All Fields]) OR "respiratory distress syndrome"[All Fields] OR "ards"[All Fields]))) NOT ("animals"[MeSH Terms:noexp] OR "animal"[All Fields])) AND ((clinicalstudy[Filter] OR clinicaltrial[Filter] OR randomizedcontrolledtrial[Filter]) AND (humans[Filter]) AND (english[Filter]) AND (2019:2022[pdat])) | 17,815 |
| 21 | ((SARS-CoV-2 OR COVID-19 OR Coronavirus Infections OR 2019-nCoV OR coronavirus disease 2019 OR Hospitalization) AND (Prevalence OR Risk Factors OR Risk Assessment OR Epidemiology OR Mortality)) AND (Coronary Artery Disease OR Cardiovascular Diseases OR Acute Coronary Syndrome OR Chronic Disease OR Disease Attributes OR Respiratory Distress Syndrome OR ARDS) NOT animal | Clinical Study, Clinical Trial, Clinical Trial, Phase I, Randomized Controlled Trial, Humans, English, from 2019 - 2022 | ((("sars cov 2"[MeSH Terms] OR "sars cov 2"[All Fields] OR "sars cov 2"[All Fields] OR ("covid 19"[All Fields] OR "covid 19"[MeSH Terms] OR "covid 19 vaccines"[All Fields] OR "covid 19 vaccines"[MeSH Terms] OR "covid 19 serotherapy"[All Fields] OR "covid 19 serotherapy"[Supplementary Concept] OR "covid 19 nucleic acid testing"[All Fields] OR "covid 19 nucleic acid testing"[MeSH Terms] OR "covid 19 serological testing"[All Fields] OR "covid 19 serological testing"[MeSH Terms] OR "covid 19 testing"[All Fields] OR "covid 19 testing"[MeSH Terms] OR "sars cov 2"[All Fields] OR "sars cov 2"[MeSH Terms] OR "severe acute respiratory syndrome coronavirus 2"[All Fields] OR "ncov"[All Fields] OR "2019 ncov"[All Fields] OR (("coronavirus"[MeSH Terms] OR "coronavirus"[All Fields] OR "cov"[All Fields]) AND 2019/11/01:3000/12/31[Date - Publication])) OR ("coronavirus infections"[MeSH Terms] OR ("coronavirus"[All Fields] AND "infections"[All Fields]) OR "coronavirus infections"[All Fields]) OR ("sars cov 2"[MeSH Terms] OR "sars cov 2"[All Fields] OR "2019 ncov"[All Fields]) OR ("covid 19"[MeSH Terms] OR "covid 19"[All Fields] OR "coronavirus disease 2019"[All Fields]) OR ("hospital s"[All Fields] OR "hospitalisation"[All Fields] OR "hospitalization"[MeSH Terms] OR "hospitalization"[All Fields] OR "hospitalising"[All Fields] OR "hospitality"[All Fields] OR "hospitalisations"[All Fields] OR "hospitalised"[All Fields] OR "hospitalizations"[All Fields] OR "hospitalized"[All Fields] OR "hospitalize"[All Fields] OR "hospitalizing"[All Fields] OR "hospitals"[MeSH Terms] OR "hospitals"[All Fields] OR "hospital"[All Fields])) AND ("epidemiology"[MeSH Subheading] OR "epidemiology"[All Fields] OR "prevalence"[All Fields] OR "prevalence"[MeSH Terms] OR "prevalance"[All Fields] OR "prevalences"[All Fields] OR "prevalence s"[All Fields] OR "prevalent"[All Fields] OR "prevalently"[All Fields] OR "prevalents"[All Fields] OR ("risk factors"[MeSH Terms] OR ("risk"[All Fields] AND "factors"[All Fields]) OR "risk factors"[All Fields]) OR ("risk assessment"[MeSH Terms] OR ("risk"[All Fields] AND "assessment"[All Fields]) OR "risk assessment"[All Fields]) OR ("epidemiologies"[All Fields] OR "epidemiology"[MeSH Subheading] OR "epidemiology"[All Fields] OR "epidemiology"[MeSH Terms] OR "epidemiology s"[All Fields]) OR ("mortality"[MeSH Terms] OR "mortality"[All Fields] OR "mortalities"[All Fields] OR "mortality"[MeSH Subheading])) AND ("coronary artery disease"[MeSH Terms] OR ("coronary"[All Fields] AND "artery"[All Fields] AND "disease"[All Fields]) OR "coronary artery disease"[All Fields] OR ("cardiovascular diseases"[MeSH Terms] OR ("cardiovascular"[All Fields] AND "diseases"[All Fields]) OR "cardiovascular diseases"[All Fields]) OR ("acute coronary syndrome"[MeSH Terms] OR ("acute"[All Fields] AND "coronary"[All Fields] AND "syndrome"[All Fields]) OR "acute coronary syndrome"[All Fields]) OR ("chronic disease"[MeSH Terms] OR ("chronic"[All Fields] AND "disease"[All Fields]) OR "chronic disease"[All Fields]) OR ("disease attributes"[MeSH Terms] OR ("disease"[All Fields] AND "attributes"[All Fields]) OR "disease attributes"[All Fields]) OR ("respiratory distress syndrome"[MeSH Terms] OR ("respiratory"[All Fields] AND "distress"[All Fields] AND "syndrome"[All Fields]) OR "respiratory distress syndrome"[All Fields]) OR ("respiratory distress syndrome"[MeSH Terms] OR ("respiratory"[All Fields] AND "distress"[All Fields] AND "syndrome"[All Fields]) OR "respiratory distress syndrome"[All Fields] OR "ards"[All Fields]))) NOT ("animals"[MeSH Terms:noexp] OR "animal"[All Fields])) AND ((clinicalstudy[Filter] OR clinicaltrial[Filter] OR clinicaltrialphasei[Filter] OR randomizedcontrolledtrial[Filter]) AND (humans[Filter]) AND (english[Filter]) AND (2019:2022[pdat])) | 17,815 |
| 22 | ((SARS-CoV-2 OR COVID-19 OR Coronavirus Infections OR 2019-nCoV OR coronavirus disease 2019 OR Hospitalization) AND (Prevalence OR Risk Factors OR Risk Assessment OR Epidemiology OR Mortality)) AND (Coronary Artery Disease OR Cardiovascular Diseases OR Acute Coronary Syndrome OR Chronic Disease OR Disease Attributes OR Respiratory Distress Syndrome OR ARDS) NOT animal | Clinical Study, Clinical Trial, Clinical Trial, Phase I, Clinical Trial, Phase II, Randomized Controlled Trial, Humans, English, from 2019 - 2022 | ((("sars cov 2"[MeSH Terms] OR "sars cov 2"[All Fields] OR "sars cov 2"[All Fields] OR ("covid 19"[All Fields] OR "covid 19"[MeSH Terms] OR "covid 19 vaccines"[All Fields] OR "covid 19 vaccines"[MeSH Terms] OR "covid 19 serotherapy"[All Fields] OR "covid 19 serotherapy"[Supplementary Concept] OR "covid 19 nucleic acid testing"[All Fields] OR "covid 19 nucleic acid testing"[MeSH Terms] OR "covid 19 serological testing"[All Fields] OR "covid 19 serological testing"[MeSH Terms] OR "covid 19 testing"[All Fields] OR "covid 19 testing"[MeSH Terms] OR "sars cov 2"[All Fields] OR "sars cov 2"[MeSH Terms] OR "severe acute respiratory syndrome coronavirus 2"[All Fields] OR "ncov"[All Fields] OR "2019 ncov"[All Fields] OR (("coronavirus"[MeSH Terms] OR "coronavirus"[All Fields] OR "cov"[All Fields]) AND 2019/11/01:3000/12/31[Date - Publication])) OR ("coronavirus infections"[MeSH Terms] OR ("coronavirus"[All Fields] AND "infections"[All Fields]) OR "coronavirus infections"[All Fields]) OR ("sars cov 2"[MeSH Terms] OR "sars cov 2"[All Fields] OR "2019 ncov"[All Fields]) OR ("covid 19"[MeSH Terms] OR "covid 19"[All Fields] OR "coronavirus disease 2019"[All Fields]) OR ("hospital s"[All Fields] OR "hospitalisation"[All Fields] OR "hospitalization"[MeSH Terms] OR "hospitalization"[All Fields] OR "hospitalising"[All Fields] OR "hospitality"[All Fields] OR "hospitalisations"[All Fields] OR "hospitalised"[All Fields] OR "hospitalizations"[All Fields] OR "hospitalized"[All Fields] OR "hospitalize"[All Fields] OR "hospitalizing"[All Fields] OR "hospitals"[MeSH Terms] OR "hospitals"[All Fields] OR "hospital"[All Fields])) AND ("epidemiology"[MeSH Subheading] OR "epidemiology"[All Fields] OR "prevalence"[All Fields] OR "prevalence"[MeSH Terms] OR "prevalance"[All Fields] OR "prevalences"[All Fields] OR "prevalence s"[All Fields] OR "prevalent"[All Fields] OR "prevalently"[All Fields] OR "prevalents"[All Fields] OR ("risk factors"[MeSH Terms] OR ("risk"[All Fields] AND "factors"[All Fields]) OR "risk factors"[All Fields]) OR ("risk assessment"[MeSH Terms] OR ("risk"[All Fields] AND "assessment"[All Fields]) OR "risk assessment"[All Fields]) OR ("epidemiologies"[All Fields] OR "epidemiology"[MeSH Subheading] OR "epidemiology"[All Fields] OR "epidemiology"[MeSH Terms] OR "epidemiology s"[All Fields]) OR ("mortality"[MeSH Terms] OR "mortality"[All Fields] OR "mortalities"[All Fields] OR "mortality"[MeSH Subheading])) AND ("coronary artery disease"[MeSH Terms] OR ("coronary"[All Fields] AND "artery"[All Fields] AND "disease"[All Fields]) OR "coronary artery disease"[All Fields] OR ("cardiovascular diseases"[MeSH Terms] OR ("cardiovascular"[All Fields] AND "diseases"[All Fields]) OR "cardiovascular diseases"[All Fields]) OR ("acute coronary syndrome"[MeSH Terms] OR ("acute"[All Fields] AND "coronary"[All Fields] AND "syndrome"[All Fields]) OR "acute coronary syndrome"[All Fields]) OR ("chronic disease"[MeSH Terms] OR ("chronic"[All Fields] AND "disease"[All Fields]) OR "chronic disease"[All Fields]) OR ("disease attributes"[MeSH Terms] OR ("disease"[All Fields] AND "attributes"[All Fields]) OR "disease attributes"[All Fields]) OR ("respiratory distress syndrome"[MeSH Terms] OR ("respiratory"[All Fields] AND "distress"[All Fields] AND "syndrome"[All Fields]) OR "respiratory distress syndrome"[All Fields]) OR ("respiratory distress syndrome"[MeSH Terms] OR ("respiratory"[All Fields] AND "distress"[All Fields] AND "syndrome"[All Fields]) OR "respiratory distress syndrome"[All Fields] OR "ards"[All Fields]))) NOT ("animals"[MeSH Terms:noexp] OR "animal"[All Fields])) AND ((clinicalstudy[Filter] OR clinicaltrial[Filter] OR clinicaltrialphasei[Filter] OR clinicaltrialphaseii[Filter] OR randomizedcontrolledtrial[Filter]) AND (humans[Filter]) AND (english[Filter]) AND (2019:2022[pdat])) | 17,815 |
| 23 | ((SARS-CoV-2 OR COVID-19 OR Coronavirus Infections OR 2019-nCoV OR coronavirus disease 2019 OR Hospitalization) AND (Prevalence OR Risk Factors OR Risk Assessment OR Epidemiology OR Mortality)) AND (Coronary Artery Disease OR Cardiovascular Diseases OR Acute Coronary Syndrome OR Chronic Disease OR Disease Attributes OR Respiratory Distress Syndrome OR ARDS) NOT animal | Clinical Study, Clinical Trial, Clinical Trial, Phase I, Clinical Trial, Phase II, Clinical Trial, Phase III, Randomized Controlled Trial, Humans, English, from 2019 - 2022 | ((("sars cov 2"[MeSH Terms] OR "sars cov 2"[All Fields] OR "sars cov 2"[All Fields] OR ("covid 19"[All Fields] OR "covid 19"[MeSH Terms] OR "covid 19 vaccines"[All Fields] OR "covid 19 vaccines"[MeSH Terms] OR "covid 19 serotherapy"[All Fields] OR "covid 19 serotherapy"[Supplementary Concept] OR "covid 19 nucleic acid testing"[All Fields] OR "covid 19 nucleic acid testing"[MeSH Terms] OR "covid 19 serological testing"[All Fields] OR "covid 19 serological testing"[MeSH Terms] OR "covid 19 testing"[All Fields] OR "covid 19 testing"[MeSH Terms] OR "sars cov 2"[All Fields] OR "sars cov 2"[MeSH Terms] OR "severe acute respiratory syndrome coronavirus 2"[All Fields] OR "ncov"[All Fields] OR "2019 ncov"[All Fields] OR (("coronavirus"[MeSH Terms] OR "coronavirus"[All Fields] OR "cov"[All Fields]) AND 2019/11/01:3000/12/31[Date - Publication])) OR ("coronavirus infections"[MeSH Terms] OR ("coronavirus"[All Fields] AND "infections"[All Fields]) OR "coronavirus infections"[All Fields]) OR ("sars cov 2"[MeSH Terms] OR "sars cov 2"[All Fields] OR "2019 ncov"[All Fields]) OR ("covid 19"[MeSH Terms] OR "covid 19"[All Fields] OR "coronavirus disease 2019"[All Fields]) OR ("hospital s"[All Fields] OR "hospitalisation"[All Fields] OR "hospitalization"[MeSH Terms] OR "hospitalization"[All Fields] OR "hospitalising"[All Fields] OR "hospitality"[All Fields] OR "hospitalisations"[All Fields] OR "hospitalised"[All Fields] OR "hospitalizations"[All Fields] OR "hospitalized"[All Fields] OR "hospitalize"[All Fields] OR "hospitalizing"[All Fields] OR "hospitals"[MeSH Terms] OR "hospitals"[All Fields] OR "hospital"[All Fields])) AND ("epidemiology"[MeSH Subheading] OR "epidemiology"[All Fields] OR "prevalence"[All Fields] OR "prevalence"[MeSH Terms] OR "prevalance"[All Fields] OR "prevalences"[All Fields] OR "prevalence s"[All Fields] OR "prevalent"[All Fields] OR "prevalently"[All Fields] OR "prevalents"[All Fields] OR ("risk factors"[MeSH Terms] OR ("risk"[All Fields] AND "factors"[All Fields]) OR "risk factors"[All Fields]) OR ("risk assessment"[MeSH Terms] OR ("risk"[All Fields] AND "assessment"[All Fields]) OR "risk assessment"[All Fields]) OR ("epidemiologies"[All Fields] OR "epidemiology"[MeSH Subheading] OR "epidemiology"[All Fields] OR "epidemiology"[MeSH Terms] OR "epidemiology s"[All Fields]) OR ("mortality"[MeSH Terms] OR "mortality"[All Fields] OR "mortalities"[All Fields] OR "mortality"[MeSH Subheading])) AND ("coronary artery disease"[MeSH Terms] OR ("coronary"[All Fields] AND "artery"[All Fields] AND "disease"[All Fields]) OR "coronary artery disease"[All Fields] OR ("cardiovascular diseases"[MeSH Terms] OR ("cardiovascular"[All Fields] AND "diseases"[All Fields]) OR "cardiovascular diseases"[All Fields]) OR ("acute coronary syndrome"[MeSH Terms] OR ("acute"[All Fields] AND "coronary"[All Fields] AND "syndrome"[All Fields]) OR "acute coronary syndrome"[All Fields]) OR ("chronic disease"[MeSH Terms] OR ("chronic"[All Fields] AND "disease"[All Fields]) OR "chronic disease"[All Fields]) OR ("disease attributes"[MeSH Terms] OR ("disease"[All Fields] AND "attributes"[All Fields]) OR "disease attributes"[All Fields]) OR ("respiratory distress syndrome"[MeSH Terms] OR ("respiratory"[All Fields] AND "distress"[All Fields] AND "syndrome"[All Fields]) OR "respiratory distress syndrome"[All Fields]) OR ("respiratory distress syndrome"[MeSH Terms] OR ("respiratory"[All Fields] AND "distress"[All Fields] AND "syndrome"[All Fields]) OR "respiratory distress syndrome"[All Fields] OR "ards"[All Fields]))) NOT ("animals"[MeSH Terms:noexp] OR "animal"[All Fields])) AND ((clinicalstudy[Filter] OR clinicaltrial[Filter] OR clinicaltrialphasei[Filter] OR clinicaltrialphaseii[Filter] OR clinicaltrialphaseiii[Filter] OR randomizedcontrolledtrial[Filter]) AND (humans[Filter]) AND (english[Filter]) AND (2019:2022[pdat])) | 17,815 |
| 24 | ((SARS-CoV-2 OR COVID-19 OR Coronavirus Infections OR 2019-nCoV OR coronavirus disease 2019 OR Hospitalization) AND (Prevalence OR Risk Factors OR Risk Assessment OR Epidemiology OR Mortality)) AND (Coronary Artery Disease OR Cardiovascular Diseases OR Acute Coronary Syndrome OR Chronic Disease OR Disease Attributes OR Respiratory Distress Syndrome OR ARDS) NOT animal | Clinical Study, Clinical Trial, Clinical Trial, Phase I, Clinical Trial, Phase II, Clinical Trial, Phase III, Clinical Trial, Phase IV, Randomized Controlled Trial, Humans, English, from 2019 - 2022 | ((("sars cov 2"[MeSH Terms] OR "sars cov 2"[All Fields] OR "sars cov 2"[All Fields] OR ("covid 19"[All Fields] OR "covid 19"[MeSH Terms] OR "covid 19 vaccines"[All Fields] OR "covid 19 vaccines"[MeSH Terms] OR "covid 19 serotherapy"[All Fields] OR "covid 19 serotherapy"[Supplementary Concept] OR "covid 19 nucleic acid testing"[All Fields] OR "covid 19 nucleic acid testing"[MeSH Terms] OR "covid 19 serological testing"[All Fields] OR "covid 19 serological testing"[MeSH Terms] OR "covid 19 testing"[All Fields] OR "covid 19 testing"[MeSH Terms] OR "sars cov 2"[All Fields] OR "sars cov 2"[MeSH Terms] OR "severe acute respiratory syndrome coronavirus 2"[All Fields] OR "ncov"[All Fields] OR "2019 ncov"[All Fields] OR (("coronavirus"[MeSH Terms] OR "coronavirus"[All Fields] OR "cov"[All Fields]) AND 2019/11/01:3000/12/31[Date - Publication])) OR ("coronavirus infections"[MeSH Terms] OR ("coronavirus"[All Fields] AND "infections"[All Fields]) OR "coronavirus infections"[All Fields]) OR ("sars cov 2"[MeSH Terms] OR "sars cov 2"[All Fields] OR "2019 ncov"[All Fields]) OR ("covid 19"[MeSH Terms] OR "covid 19"[All Fields] OR "coronavirus disease 2019"[All Fields]) OR ("hospital s"[All Fields] OR "hospitalisation"[All Fields] OR "hospitalization"[MeSH Terms] OR "hospitalization"[All Fields] OR "hospitalising"[All Fields] OR "hospitality"[All Fields] OR "hospitalisations"[All Fields] OR "hospitalised"[All Fields] OR "hospitalizations"[All Fields] OR "hospitalized"[All Fields] OR "hospitalize"[All Fields] OR "hospitalizing"[All Fields] OR "hospitals"[MeSH Terms] OR "hospitals"[All Fields] OR "hospital"[All Fields])) AND ("epidemiology"[MeSH Subheading] OR "epidemiology"[All Fields] OR "prevalence"[All Fields] OR "prevalence"[MeSH Terms] OR "prevalance"[All Fields] OR "prevalences"[All Fields] OR "prevalence s"[All Fields] OR "prevalent"[All Fields] OR "prevalently"[All Fields] OR "prevalents"[All Fields] OR ("risk factors"[MeSH Terms] OR ("risk"[All Fields] AND "factors"[All Fields]) OR "risk factors"[All Fields]) OR ("risk assessment"[MeSH Terms] OR ("risk"[All Fields] AND "assessment"[All Fields]) OR "risk assessment"[All Fields]) OR ("epidemiologies"[All Fields] OR "epidemiology"[MeSH Subheading] OR "epidemiology"[All Fields] OR "epidemiology"[MeSH Terms] OR "epidemiology s"[All Fields]) OR ("mortality"[MeSH Terms] OR "mortality"[All Fields] OR "mortalities"[All Fields] OR "mortality"[MeSH Subheading])) AND ("coronary artery disease"[MeSH Terms] OR ("coronary"[All Fields] AND "artery"[All Fields] AND "disease"[All Fields]) OR "coronary artery disease"[All Fields] OR ("cardiovascular diseases"[MeSH Terms] OR ("cardiovascular"[All Fields] AND "diseases"[All Fields]) OR "cardiovascular diseases"[All Fields]) OR ("acute coronary syndrome"[MeSH Terms] OR ("acute"[All Fields] AND "coronary"[All Fields] AND "syndrome"[All Fields]) OR "acute coronary syndrome"[All Fields]) OR ("chronic disease"[MeSH Terms] OR ("chronic"[All Fields] AND "disease"[All Fields]) OR "chronic disease"[All Fields]) OR ("disease attributes"[MeSH Terms] OR ("disease"[All Fields] AND "attributes"[All Fields]) OR "disease attributes"[All Fields]) OR ("respiratory distress syndrome"[MeSH Terms] OR ("respiratory"[All Fields] AND "distress"[All Fields] AND "syndrome"[All Fields]) OR "respiratory distress syndrome"[All Fields]) OR ("respiratory distress syndrome"[MeSH Terms] OR ("respiratory"[All Fields] AND "distress"[All Fields] AND "syndrome"[All Fields]) OR "respiratory distress syndrome"[All Fields] OR "ards"[All Fields]))) NOT ("animals"[MeSH Terms:noexp] OR "animal"[All Fields])) AND ((clinicalstudy[Filter] OR clinicaltrial[Filter] OR clinicaltrialphasei[Filter] OR clinicaltrialphaseii[Filter] OR clinicaltrialphaseiii[Filter] OR clinicaltrialphaseiv[Filter] OR randomizedcontrolledtrial[Filter]) AND (humans[Filter]) AND (english[Filter]) AND (2019:2022[pdat])) | 17,815 |
| 25 | ((SARS-CoV-2 OR COVID-19 OR Coronavirus Infections OR 2019-nCoV OR coronavirus disease 2019 OR Hospitalization) AND (Prevalence OR Risk Factors OR Risk Assessment OR Epidemiology OR Mortality)) AND (Coronary Artery Disease OR Cardiovascular Diseases OR Acute Coronary Syndrome OR Chronic Disease OR Disease Attributes OR Respiratory Distress Syndrome OR ARDS) NOT animal | Clinical Study, Clinical Trial, Clinical Trial, Phase I, Clinical Trial, Phase II, Clinical Trial, Phase III, Clinical Trial, Phase IV, Comparative Study, Randomized Controlled Trial, Humans, English, from 2019 - 2022 | ((("sars cov 2"[MeSH Terms] OR "sars cov 2"[All Fields] OR "sars cov 2"[All Fields] OR ("covid 19"[All Fields] OR "covid 19"[MeSH Terms] OR "covid 19 vaccines"[All Fields] OR "covid 19 vaccines"[MeSH Terms] OR "covid 19 serotherapy"[All Fields] OR "covid 19 serotherapy"[Supplementary Concept] OR "covid 19 nucleic acid testing"[All Fields] OR "covid 19 nucleic acid testing"[MeSH Terms] OR "covid 19 serological testing"[All Fields] OR "covid 19 serological testing"[MeSH Terms] OR "covid 19 testing"[All Fields] OR "covid 19 testing"[MeSH Terms] OR "sars cov 2"[All Fields] OR "sars cov 2"[MeSH Terms] OR "severe acute respiratory syndrome coronavirus 2"[All Fields] OR "ncov"[All Fields] OR "2019 ncov"[All Fields] OR (("coronavirus"[MeSH Terms] OR "coronavirus"[All Fields] OR "cov"[All Fields]) AND 2019/11/01:3000/12/31[Date - Publication])) OR ("coronavirus infections"[MeSH Terms] OR ("coronavirus"[All Fields] AND "infections"[All Fields]) OR "coronavirus infections"[All Fields]) OR ("sars cov 2"[MeSH Terms] OR "sars cov 2"[All Fields] OR "2019 ncov"[All Fields]) OR ("covid 19"[MeSH Terms] OR "covid 19"[All Fields] OR "coronavirus disease 2019"[All Fields]) OR ("hospital s"[All Fields] OR "hospitalisation"[All Fields] OR "hospitalization"[MeSH Terms] OR "hospitalization"[All Fields] OR "hospitalising"[All Fields] OR "hospitality"[All Fields] OR "hospitalisations"[All Fields] OR "hospitalised"[All Fields] OR "hospitalizations"[All Fields] OR "hospitalized"[All Fields] OR "hospitalize"[All Fields] OR "hospitalizing"[All Fields] OR "hospitals"[MeSH Terms] OR "hospitals"[All Fields] OR "hospital"[All Fields])) AND ("epidemiology"[MeSH Subheading] OR "epidemiology"[All Fields] OR "prevalence"[All Fields] OR "prevalence"[MeSH Terms] OR "prevalance"[All Fields] OR "prevalences"[All Fields] OR "prevalence s"[All Fields] OR "prevalent"[All Fields] OR "prevalently"[All Fields] OR "prevalents"[All Fields] OR ("risk factors"[MeSH Terms] OR ("risk"[All Fields] AND "factors"[All Fields]) OR "risk factors"[All Fields]) OR ("risk assessment"[MeSH Terms] OR ("risk"[All Fields] AND "assessment"[All Fields]) OR "risk assessment"[All Fields]) OR ("epidemiologies"[All Fields] OR "epidemiology"[MeSH Subheading] OR "epidemiology"[All Fields] OR "epidemiology"[MeSH Terms] OR "epidemiology s"[All Fields]) OR ("mortality"[MeSH Terms] OR "mortality"[All Fields] OR "mortalities"[All Fields] OR "mortality"[MeSH Subheading])) AND ("coronary artery disease"[MeSH Terms] OR ("coronary"[All Fields] AND "artery"[All Fields] AND "disease"[All Fields]) OR "coronary artery disease"[All Fields] OR ("cardiovascular diseases"[MeSH Terms] OR ("cardiovascular"[All Fields] AND "diseases"[All Fields]) OR "cardiovascular diseases"[All Fields]) OR ("acute coronary syndrome"[MeSH Terms] OR ("acute"[All Fields] AND "coronary"[All Fields] AND "syndrome"[All Fields]) OR "acute coronary syndrome"[All Fields]) OR ("chronic disease"[MeSH Terms] OR ("chronic"[All Fields] AND "disease"[All Fields]) OR "chronic disease"[All Fields]) OR ("disease attributes"[MeSH Terms] OR ("disease"[All Fields] AND "attributes"[All Fields]) OR "disease attributes"[All Fields]) OR ("respiratory distress syndrome"[MeSH Terms] OR ("respiratory"[All Fields] AND "distress"[All Fields] AND "syndrome"[All Fields]) OR "respiratory distress syndrome"[All Fields]) OR ("respiratory distress syndrome"[MeSH Terms] OR ("respiratory"[All Fields] AND "distress"[All Fields] AND "syndrome"[All Fields]) OR "respiratory distress syndrome"[All Fields] OR "ards"[All Fields]))) NOT ("animals"[MeSH Terms:noexp] OR "animal"[All Fields])) AND ((clinicalstudy[Filter] OR clinicaltrial[Filter] OR clinicaltrialphasei[Filter] OR clinicaltrialphaseii[Filter] OR clinicaltrialphaseiii[Filter] OR clinicaltrialphaseiv[Filter] OR comparativestudy[Filter] OR randomizedcontrolledtrial[Filter]) AND (humans[Filter]) AND (english[Filter]) AND (2019:2022[pdat])) | 22,944 |
| 26 | ((SARS-CoV-2 OR COVID-19 OR Coronavirus Infections OR 2019-nCoV OR coronavirus disease 2019 OR Hospitalization) AND (Prevalence OR Risk Factors OR Risk Assessment OR Epidemiology OR Mortality)) AND (Coronary Artery Disease OR Cardiovascular Diseases OR Acute Coronary Syndrome OR Chronic Disease OR Disease Attributes OR Respiratory Distress Syndrome OR ARDS) NOT animal | Clinical Study, Clinical Trial, Clinical Trial, Phase I, Clinical Trial, Phase II, Clinical Trial, Phase III, Clinical Trial, Phase IV, Comparative Study, Controlled Clinical Trial, Randomized Controlled Trial, Humans, English, from 2019 - 2022 | ((("sars cov 2"[MeSH Terms] OR "sars cov 2"[All Fields] OR "sars cov 2"[All Fields] OR ("covid 19"[All Fields] OR "covid 19"[MeSH Terms] OR "covid 19 vaccines"[All Fields] OR "covid 19 vaccines"[MeSH Terms] OR "covid 19 serotherapy"[All Fields] OR "covid 19 serotherapy"[Supplementary Concept] OR "covid 19 nucleic acid testing"[All Fields] OR "covid 19 nucleic acid testing"[MeSH Terms] OR "covid 19 serological testing"[All Fields] OR "covid 19 serological testing"[MeSH Terms] OR "covid 19 testing"[All Fields] OR "covid 19 testing"[MeSH Terms] OR "sars cov 2"[All Fields] OR "sars cov 2"[MeSH Terms] OR "severe acute respiratory syndrome coronavirus 2"[All Fields] OR "ncov"[All Fields] OR "2019 ncov"[All Fields] OR (("coronavirus"[MeSH Terms] OR "coronavirus"[All Fields] OR "cov"[All Fields]) AND 2019/11/01:3000/12/31[Date - Publication])) OR ("coronavirus infections"[MeSH Terms] OR ("coronavirus"[All Fields] AND "infections"[All Fields]) OR "coronavirus infections"[All Fields]) OR ("sars cov 2"[MeSH Terms] OR "sars cov 2"[All Fields] OR "2019 ncov"[All Fields]) OR ("covid 19"[MeSH Terms] OR "covid 19"[All Fields] OR "coronavirus disease 2019"[All Fields]) OR ("hospital s"[All Fields] OR "hospitalisation"[All Fields] OR "hospitalization"[MeSH Terms] OR "hospitalization"[All Fields] OR "hospitalising"[All Fields] OR "hospitality"[All Fields] OR "hospitalisations"[All Fields] OR "hospitalised"[All Fields] OR "hospitalizations"[All Fields] OR "hospitalized"[All Fields] OR "hospitalize"[All Fields] OR "hospitalizing"[All Fields] OR "hospitals"[MeSH Terms] OR "hospitals"[All Fields] OR "hospital"[All Fields])) AND ("epidemiology"[MeSH Subheading] OR "epidemiology"[All Fields] OR "prevalence"[All Fields] OR "prevalence"[MeSH Terms] OR "prevalance"[All Fields] OR "prevalences"[All Fields] OR "prevalence s"[All Fields] OR "prevalent"[All Fields] OR "prevalently"[All Fields] OR "prevalents"[All Fields] OR ("risk factors"[MeSH Terms] OR ("risk"[All Fields] AND "factors"[All Fields]) OR "risk factors"[All Fields]) OR ("risk assessment"[MeSH Terms] OR ("risk"[All Fields] AND "assessment"[All Fields]) OR "risk assessment"[All Fields]) OR ("epidemiologies"[All Fields] OR "epidemiology"[MeSH Subheading] OR "epidemiology"[All Fields] OR "epidemiology"[MeSH Terms] OR "epidemiology s"[All Fields]) OR ("mortality"[MeSH Terms] OR "mortality"[All Fields] OR "mortalities"[All Fields] OR "mortality"[MeSH Subheading])) AND ("coronary artery disease"[MeSH Terms] OR ("coronary"[All Fields] AND "artery"[All Fields] AND "disease"[All Fields]) OR "coronary artery disease"[All Fields] OR ("cardiovascular diseases"[MeSH Terms] OR ("cardiovascular"[All Fields] AND "diseases"[All Fields]) OR "cardiovascular diseases"[All Fields]) OR ("acute coronary syndrome"[MeSH Terms] OR ("acute"[All Fields] AND "coronary"[All Fields] AND "syndrome"[All Fields]) OR "acute coronary syndrome"[All Fields]) OR ("chronic disease"[MeSH Terms] OR ("chronic"[All Fields] AND "disease"[All Fields]) OR "chronic disease"[All Fields]) OR ("disease attributes"[MeSH Terms] OR ("disease"[All Fields] AND "attributes"[All Fields]) OR "disease attributes"[All Fields]) OR ("respiratory distress syndrome"[MeSH Terms] OR ("respiratory"[All Fields] AND "distress"[All Fields] AND "syndrome"[All Fields]) OR "respiratory distress syndrome"[All Fields]) OR ("respiratory distress syndrome"[MeSH Terms] OR ("respiratory"[All Fields] AND "distress"[All Fields] AND "syndrome"[All Fields]) OR "respiratory distress syndrome"[All Fields] OR "ards"[All Fields]))) NOT ("animals"[MeSH Terms:noexp] OR "animal"[All Fields])) AND ((clinicalstudy[Filter] OR clinicaltrial[Filter] OR clinicaltrialphasei[Filter] OR clinicaltrialphaseii[Filter] OR clinicaltrialphaseiii[Filter] OR clinicaltrialphaseiv[Filter] OR comparativestudy[Filter] OR controlledclinicaltrial[Filter] OR randomizedcontrolledtrial[Filter]) AND (humans[Filter]) AND (english[Filter]) AND (2019:2022[pdat])) | 22,944 |
| 27 | ((SARS-CoV-2 OR COVID-19 OR Coronavirus Infections OR 2019-nCoV OR coronavirus disease 2019 OR Hospitalization) AND (Prevalence OR Risk Factors OR Risk Assessment OR Epidemiology OR Mortality)) AND (Coronary Artery Disease OR Cardiovascular Diseases OR Acute Coronary Syndrome OR Chronic Disease OR Disease Attributes OR Respiratory Distress Syndrome OR ARDS) NOT animal | Clinical Study, Clinical Trial, Clinical Trial, Phase I, Clinical Trial, Phase II, Clinical Trial, Phase III, Clinical Trial, Phase IV, Comparative Study, Controlled Clinical Trial, Corrected and Republished Article, Randomized Controlled Trial, Humans, English, from 2019 - 2022 | ((("sars cov 2"[MeSH Terms] OR "sars cov 2"[All Fields] OR "sars cov 2"[All Fields] OR ("covid 19"[All Fields] OR "covid 19"[MeSH Terms] OR "covid 19 vaccines"[All Fields] OR "covid 19 vaccines"[MeSH Terms] OR "covid 19 serotherapy"[All Fields] OR "covid 19 serotherapy"[Supplementary Concept] OR "covid 19 nucleic acid testing"[All Fields] OR "covid 19 nucleic acid testing"[MeSH Terms] OR "covid 19 serological testing"[All Fields] OR "covid 19 serological testing"[MeSH Terms] OR "covid 19 testing"[All Fields] OR "covid 19 testing"[MeSH Terms] OR "sars cov 2"[All Fields] OR "sars cov 2"[MeSH Terms] OR "severe acute respiratory syndrome coronavirus 2"[All Fields] OR "ncov"[All Fields] OR "2019 ncov"[All Fields] OR (("coronavirus"[MeSH Terms] OR "coronavirus"[All Fields] OR "cov"[All Fields]) AND 2019/11/01:3000/12/31[Date - Publication])) OR ("coronavirus infections"[MeSH Terms] OR ("coronavirus"[All Fields] AND "infections"[All Fields]) OR "coronavirus infections"[All Fields]) OR ("sars cov 2"[MeSH Terms] OR "sars cov 2"[All Fields] OR "2019 ncov"[All Fields]) OR ("covid 19"[MeSH Terms] OR "covid 19"[All Fields] OR "coronavirus disease 2019"[All Fields]) OR ("hospital s"[All Fields] OR "hospitalisation"[All Fields] OR "hospitalization"[MeSH Terms] OR "hospitalization"[All Fields] OR "hospitalising"[All Fields] OR "hospitality"[All Fields] OR "hospitalisations"[All Fields] OR "hospitalised"[All Fields] OR "hospitalizations"[All Fields] OR "hospitalized"[All Fields] OR "hospitalize"[All Fields] OR "hospitalizing"[All Fields] OR "hospitals"[MeSH Terms] OR "hospitals"[All Fields] OR "hospital"[All Fields])) AND ("epidemiology"[MeSH Subheading] OR "epidemiology"[All Fields] OR "prevalence"[All Fields] OR "prevalence"[MeSH Terms] OR "prevalance"[All Fields] OR "prevalences"[All Fields] OR "prevalence s"[All Fields] OR "prevalent"[All Fields] OR "prevalently"[All Fields] OR "prevalents"[All Fields] OR ("risk factors"[MeSH Terms] OR ("risk"[All Fields] AND "factors"[All Fields]) OR "risk factors"[All Fields]) OR ("risk assessment"[MeSH Terms] OR ("risk"[All Fields] AND "assessment"[All Fields]) OR "risk assessment"[All Fields]) OR ("epidemiologies"[All Fields] OR "epidemiology"[MeSH Subheading] OR "epidemiology"[All Fields] OR "epidemiology"[MeSH Terms] OR "epidemiology s"[All Fields]) OR ("mortality"[MeSH Terms] OR "mortality"[All Fields] OR "mortalities"[All Fields] OR "mortality"[MeSH Subheading])) AND ("coronary artery disease"[MeSH Terms] OR ("coronary"[All Fields] AND "artery"[All Fields] AND "disease"[All Fields]) OR "coronary artery disease"[All Fields] OR ("cardiovascular diseases"[MeSH Terms] OR ("cardiovascular"[All Fields] AND "diseases"[All Fields]) OR "cardiovascular diseases"[All Fields]) OR ("acute coronary syndrome"[MeSH Terms] OR ("acute"[All Fields] AND "coronary"[All Fields] AND "syndrome"[All Fields]) OR "acute coronary syndrome"[All Fields]) OR ("chronic disease"[MeSH Terms] OR ("chronic"[All Fields] AND "disease"[All Fields]) OR "chronic disease"[All Fields]) OR ("disease attributes"[MeSH Terms] OR ("disease"[All Fields] AND "attributes"[All Fields]) OR "disease attributes"[All Fields]) OR ("respiratory distress syndrome"[MeSH Terms] OR ("respiratory"[All Fields] AND "distress"[All Fields] AND "syndrome"[All Fields]) OR "respiratory distress syndrome"[All Fields]) OR ("respiratory distress syndrome"[MeSH Terms] OR ("respiratory"[All Fields] AND "distress"[All Fields] AND "syndrome"[All Fields]) OR "respiratory distress syndrome"[All Fields] OR "ards"[All Fields]))) NOT ("animals"[MeSH Terms:noexp] OR "animal"[All Fields])) AND ((clinicalstudy[Filter] OR clinicaltrial[Filter] OR clinicaltrialphasei[Filter] OR clinicaltrialphaseii[Filter] OR clinicaltrialphaseiii[Filter] OR clinicaltrialphaseiv[Filter] OR comparativestudy[Filter] OR controlledclinicaltrial[Filter] OR correctedandrepublishedarticle[Filter] OR randomizedcontrolledtrial[Filter]) AND (humans[Filter]) AND (english[Filter]) AND (2019:2022[pdat])) | 22,944 |
| 28 | ((SARS-CoV-2 OR COVID-19 OR Coronavirus Infections OR 2019-nCoV OR coronavirus disease 2019 OR Hospitalization) AND (Prevalence OR Risk Factors OR Risk Assessment OR Epidemiology OR Mortality)) AND (Coronary Artery Disease OR Cardiovascular Diseases OR Acute Coronary Syndrome OR Chronic Disease OR Disease Attributes OR Respiratory Distress Syndrome OR ARDS) NOT animal | Clinical Study, Clinical Trial, Clinical Trial, Phase I, Clinical Trial, Phase II, Clinical Trial, Phase III, Clinical Trial, Phase IV, Comparative Study, Controlled Clinical Trial, Corrected and Republished Article, Dataset, Randomized Controlled Trial, Humans, English, from 2019 - 2022 | ((("sars cov 2"[MeSH Terms] OR "sars cov 2"[All Fields] OR "sars cov 2"[All Fields] OR ("covid 19"[All Fields] OR "covid 19"[MeSH Terms] OR "covid 19 vaccines"[All Fields] OR "covid 19 vaccines"[MeSH Terms] OR "covid 19 serotherapy"[All Fields] OR "covid 19 serotherapy"[Supplementary Concept] OR "covid 19 nucleic acid testing"[All Fields] OR "covid 19 nucleic acid testing"[MeSH Terms] OR "covid 19 serological testing"[All Fields] OR "covid 19 serological testing"[MeSH Terms] OR "covid 19 testing"[All Fields] OR "covid 19 testing"[MeSH Terms] OR "sars cov 2"[All Fields] OR "sars cov 2"[MeSH Terms] OR "severe acute respiratory syndrome coronavirus 2"[All Fields] OR "ncov"[All Fields] OR "2019 ncov"[All Fields] OR (("coronavirus"[MeSH Terms] OR "coronavirus"[All Fields] OR "cov"[All Fields]) AND 2019/11/01:3000/12/31[Date - Publication])) OR ("coronavirus infections"[MeSH Terms] OR ("coronavirus"[All Fields] AND "infections"[All Fields]) OR "coronavirus infections"[All Fields]) OR ("sars cov 2"[MeSH Terms] OR "sars cov 2"[All Fields] OR "2019 ncov"[All Fields]) OR ("covid 19"[MeSH Terms] OR "covid 19"[All Fields] OR "coronavirus disease 2019"[All Fields]) OR ("hospital s"[All Fields] OR "hospitalisation"[All Fields] OR "hospitalization"[MeSH Terms] OR "hospitalization"[All Fields] OR "hospitalising"[All Fields] OR "hospitality"[All Fields] OR "hospitalisations"[All Fields] OR "hospitalised"[All Fields] OR "hospitalizations"[All Fields] OR "hospitalized"[All Fields] OR "hospitalize"[All Fields] OR "hospitalizing"[All Fields] OR "hospitals"[MeSH Terms] OR "hospitals"[All Fields] OR "hospital"[All Fields])) AND ("epidemiology"[MeSH Subheading] OR "epidemiology"[All Fields] OR "prevalence"[All Fields] OR "prevalence"[MeSH Terms] OR "prevalance"[All Fields] OR "prevalences"[All Fields] OR "prevalence s"[All Fields] OR "prevalent"[All Fields] OR "prevalently"[All Fields] OR "prevalents"[All Fields] OR ("risk factors"[MeSH Terms] OR ("risk"[All Fields] AND "factors"[All Fields]) OR "risk factors"[All Fields]) OR ("risk assessment"[MeSH Terms] OR ("risk"[All Fields] AND "assessment"[All Fields]) OR "risk assessment"[All Fields]) OR ("epidemiologies"[All Fields] OR "epidemiology"[MeSH Subheading] OR "epidemiology"[All Fields] OR "epidemiology"[MeSH Terms] OR "epidemiology s"[All Fields]) OR ("mortality"[MeSH Terms] OR "mortality"[All Fields] OR "mortalities"[All Fields] OR "mortality"[MeSH Subheading])) AND ("coronary artery disease"[MeSH Terms] OR ("coronary"[All Fields] AND "artery"[All Fields] AND "disease"[All Fields]) OR "coronary artery disease"[All Fields] OR ("cardiovascular diseases"[MeSH Terms] OR ("cardiovascular"[All Fields] AND "diseases"[All Fields]) OR "cardiovascular diseases"[All Fields]) OR ("acute coronary syndrome"[MeSH Terms] OR ("acute"[All Fields] AND "coronary"[All Fields] AND "syndrome"[All Fields]) OR "acute coronary syndrome"[All Fields]) OR ("chronic disease"[MeSH Terms] OR ("chronic"[All Fields] AND "disease"[All Fields]) OR "chronic disease"[All Fields]) OR ("disease attributes"[MeSH Terms] OR ("disease"[All Fields] AND "attributes"[All Fields]) OR "disease attributes"[All Fields]) OR ("respiratory distress syndrome"[MeSH Terms] OR ("respiratory"[All Fields] AND "distress"[All Fields] AND "syndrome"[All Fields]) OR "respiratory distress syndrome"[All Fields]) OR ("respiratory distress syndrome"[MeSH Terms] OR ("respiratory"[All Fields] AND "distress"[All Fields] AND "syndrome"[All Fields]) OR "respiratory distress syndrome"[All Fields] OR "ards"[All Fields]))) NOT ("animals"[MeSH Terms:noexp] OR "animal"[All Fields])) AND ((clinicalstudy[Filter] OR clinicaltrial[Filter] OR clinicaltrialphasei[Filter] OR clinicaltrialphaseii[Filter] OR clinicaltrialphaseiii[Filter] OR clinicaltrialphaseiv[Filter] OR comparativestudy[Filter] OR controlledclinicaltrial[Filter] OR correctedandrepublishedarticle[Filter] OR dataset[Filter] OR randomizedcontrolledtrial[Filter]) AND (humans[Filter]) AND (english[Filter]) AND (2019:2022[pdat])) | 22,946 |
| 29 | ((SARS-CoV-2 OR COVID-19 OR Coronavirus Infections OR 2019-nCoV OR coronavirus disease 2019 OR Hospitalization) AND (Prevalence OR Risk Factors OR Risk Assessment OR Epidemiology OR Mortality)) AND (Coronary Artery Disease OR Cardiovascular Diseases OR Acute Coronary Syndrome OR Chronic Disease OR Disease Attributes OR Respiratory Distress Syndrome OR ARDS) NOT animal | Clinical Study, Clinical Trial, Clinical Trial, Phase I, Clinical Trial, Phase II, Clinical Trial, Phase III, Clinical Trial, Phase IV, Comparative Study, Controlled Clinical Trial, Corrected and Republished Article, Dataset, Electronic Supplementary Materials, Randomized Controlled Trial, Humans, English, from 2019 - 2022 | ((("sars cov 2"[MeSH Terms] OR "sars cov 2"[All Fields] OR "sars cov 2"[All Fields] OR ("covid 19"[All Fields] OR "covid 19"[MeSH Terms] OR "covid 19 vaccines"[All Fields] OR "covid 19 vaccines"[MeSH Terms] OR "covid 19 serotherapy"[All Fields] OR "covid 19 serotherapy"[Supplementary Concept] OR "covid 19 nucleic acid testing"[All Fields] OR "covid 19 nucleic acid testing"[MeSH Terms] OR "covid 19 serological testing"[All Fields] OR "covid 19 serological testing"[MeSH Terms] OR "covid 19 testing"[All Fields] OR "covid 19 testing"[MeSH Terms] OR "sars cov 2"[All Fields] OR "sars cov 2"[MeSH Terms] OR "severe acute respiratory syndrome coronavirus 2"[All Fields] OR "ncov"[All Fields] OR "2019 ncov"[All Fields] OR (("coronavirus"[MeSH Terms] OR "coronavirus"[All Fields] OR "cov"[All Fields]) AND 2019/11/01:3000/12/31[Date - Publication])) OR ("coronavirus infections"[MeSH Terms] OR ("coronavirus"[All Fields] AND "infections"[All Fields]) OR "coronavirus infections"[All Fields]) OR ("sars cov 2"[MeSH Terms] OR "sars cov 2"[All Fields] OR "2019 ncov"[All Fields]) OR ("covid 19"[MeSH Terms] OR "covid 19"[All Fields] OR "coronavirus disease 2019"[All Fields]) OR ("hospital s"[All Fields] OR "hospitalisation"[All Fields] OR "hospitalization"[MeSH Terms] OR "hospitalization"[All Fields] OR "hospitalising"[All Fields] OR "hospitality"[All Fields] OR "hospitalisations"[All Fields] OR "hospitalised"[All Fields] OR "hospitalizations"[All Fields] OR "hospitalized"[All Fields] OR "hospitalize"[All Fields] OR "hospitalizing"[All Fields] OR "hospitals"[MeSH Terms] OR "hospitals"[All Fields] OR "hospital"[All Fields])) AND ("epidemiology"[MeSH Subheading] OR "epidemiology"[All Fields] OR "prevalence"[All Fields] OR "prevalence"[MeSH Terms] OR "prevalance"[All Fields] OR "prevalences"[All Fields] OR "prevalence s"[All Fields] OR "prevalent"[All Fields] OR "prevalently"[All Fields] OR "prevalents"[All Fields] OR ("risk factors"[MeSH Terms] OR ("risk"[All Fields] AND "factors"[All Fields]) OR "risk factors"[All Fields]) OR ("risk assessment"[MeSH Terms] OR ("risk"[All Fields] AND "assessment"[All Fields]) OR "risk assessment"[All Fields]) OR ("epidemiologies"[All Fields] OR "epidemiology"[MeSH Subheading] OR "epidemiology"[All Fields] OR "epidemiology"[MeSH Terms] OR "epidemiology s"[All Fields]) OR ("mortality"[MeSH Terms] OR "mortality"[All Fields] OR "mortalities"[All Fields] OR "mortality"[MeSH Subheading])) AND ("coronary artery disease"[MeSH Terms] OR ("coronary"[All Fields] AND "artery"[All Fields] AND "disease"[All Fields]) OR "coronary artery disease"[All Fields] OR ("cardiovascular diseases"[MeSH Terms] OR ("cardiovascular"[All Fields] AND "diseases"[All Fields]) OR "cardiovascular diseases"[All Fields]) OR ("acute coronary syndrome"[MeSH Terms] OR ("acute"[All Fields] AND "coronary"[All Fields] AND "syndrome"[All Fields]) OR "acute coronary syndrome"[All Fields]) OR ("chronic disease"[MeSH Terms] OR ("chronic"[All Fields] AND "disease"[All Fields]) OR "chronic disease"[All Fields]) OR ("disease attributes"[MeSH Terms] OR ("disease"[All Fields] AND "attributes"[All Fields]) OR "disease attributes"[All Fields]) OR ("respiratory distress syndrome"[MeSH Terms] OR ("respiratory"[All Fields] AND "distress"[All Fields] AND "syndrome"[All Fields]) OR "respiratory distress syndrome"[All Fields]) OR ("respiratory distress syndrome"[MeSH Terms] OR ("respiratory"[All Fields] AND "distress"[All Fields] AND "syndrome"[All Fields]) OR "respiratory distress syndrome"[All Fields] OR "ards"[All Fields]))) NOT ("animals"[MeSH Terms:noexp] OR "animal"[All Fields])) AND ((clinicalstudy[Filter] OR clinicaltrial[Filter] OR clinicaltrialphasei[Filter] OR clinicaltrialphaseii[Filter] OR clinicaltrialphaseiii[Filter] OR clinicaltrialphaseiv[Filter] OR comparativestudy[Filter] OR controlledclinicaltrial[Filter] OR correctedandrepublishedarticle[Filter] OR dataset[Filter] OR electronicsupplementarymaterials[Filter] OR randomizedcontrolledtrial[Filter]) AND (humans[Filter]) AND (english[Filter]) AND (2019:2022[pdat])) | 23,378 |
| 30 | ((SARS-CoV-2 OR COVID-19 OR Coronavirus Infections OR 2019-nCoV OR coronavirus disease 2019 OR Hospitalization) AND (Prevalence OR Risk Factors OR Risk Assessment OR Epidemiology OR Mortality)) AND (Coronary Artery Disease OR Cardiovascular Diseases OR Acute Coronary Syndrome OR Chronic Disease OR Disease Attributes OR Respiratory Distress Syndrome OR ARDS) NOT animal | Clinical Study, Clinical Trial, Clinical Trial, Phase I, Clinical Trial, Phase II, Clinical Trial, Phase III, Clinical Trial, Phase IV, Comparative Study, Controlled Clinical Trial, Corrected and Republished Article, Dataset, Electronic Supplementary Materials, English Abstract, Randomized Controlled Trial, Humans, English, from 2019 - 2022 | ((("sars cov 2"[MeSH Terms] OR "sars cov 2"[All Fields] OR "sars cov 2"[All Fields] OR ("covid 19"[All Fields] OR "covid 19"[MeSH Terms] OR "covid 19 vaccines"[All Fields] OR "covid 19 vaccines"[MeSH Terms] OR "covid 19 serotherapy"[All Fields] OR "covid 19 serotherapy"[Supplementary Concept] OR "covid 19 nucleic acid testing"[All Fields] OR "covid 19 nucleic acid testing"[MeSH Terms] OR "covid 19 serological testing"[All Fields] OR "covid 19 serological testing"[MeSH Terms] OR "covid 19 testing"[All Fields] OR "covid 19 testing"[MeSH Terms] OR "sars cov 2"[All Fields] OR "sars cov 2"[MeSH Terms] OR "severe acute respiratory syndrome coronavirus 2"[All Fields] OR "ncov"[All Fields] OR "2019 ncov"[All Fields] OR (("coronavirus"[MeSH Terms] OR "coronavirus"[All Fields] OR "cov"[All Fields]) AND 2019/11/01:3000/12/31[Date - Publication])) OR ("coronavirus infections"[MeSH Terms] OR ("coronavirus"[All Fields] AND "infections"[All Fields]) OR "coronavirus infections"[All Fields]) OR ("sars cov 2"[MeSH Terms] OR "sars cov 2"[All Fields] OR "2019 ncov"[All Fields]) OR ("covid 19"[MeSH Terms] OR "covid 19"[All Fields] OR "coronavirus disease 2019"[All Fields]) OR ("hospital s"[All Fields] OR "hospitalisation"[All Fields] OR "hospitalization"[MeSH Terms] OR "hospitalization"[All Fields] OR "hospitalising"[All Fields] OR "hospitality"[All Fields] OR "hospitalisations"[All Fields] OR "hospitalised"[All Fields] OR "hospitalizations"[All Fields] OR "hospitalized"[All Fields] OR "hospitalize"[All Fields] OR "hospitalizing"[All Fields] OR "hospitals"[MeSH Terms] OR "hospitals"[All Fields] OR "hospital"[All Fields])) AND ("epidemiology"[MeSH Subheading] OR "epidemiology"[All Fields] OR "prevalence"[All Fields] OR "prevalence"[MeSH Terms] OR "prevalance"[All Fields] OR "prevalences"[All Fields] OR "prevalence s"[All Fields] OR "prevalent"[All Fields] OR "prevalently"[All Fields] OR "prevalents"[All Fields] OR ("risk factors"[MeSH Terms] OR ("risk"[All Fields] AND "factors"[All Fields]) OR "risk factors"[All Fields]) OR ("risk assessment"[MeSH Terms] OR ("risk"[All Fields] AND "assessment"[All Fields]) OR "risk assessment"[All Fields]) OR ("epidemiologies"[All Fields] OR "epidemiology"[MeSH Subheading] OR "epidemiology"[All Fields] OR "epidemiology"[MeSH Terms] OR "epidemiology s"[All Fields]) OR ("mortality"[MeSH Terms] OR "mortality"[All Fields] OR "mortalities"[All Fields] OR "mortality"[MeSH Subheading])) AND ("coronary artery disease"[MeSH Terms] OR ("coronary"[All Fields] AND "artery"[All Fields] AND "disease"[All Fields]) OR "coronary artery disease"[All Fields] OR ("cardiovascular diseases"[MeSH Terms] OR ("cardiovascular"[All Fields] AND "diseases"[All Fields]) OR "cardiovascular diseases"[All Fields]) OR ("acute coronary syndrome"[MeSH Terms] OR ("acute"[All Fields] AND "coronary"[All Fields] AND "syndrome"[All Fields]) OR "acute coronary syndrome"[All Fields]) OR ("chronic disease"[MeSH Terms] OR ("chronic"[All Fields] AND "disease"[All Fields]) OR "chronic disease"[All Fields]) OR ("disease attributes"[MeSH Terms] OR ("disease"[All Fields] AND "attributes"[All Fields]) OR "disease attributes"[All Fields]) OR ("respiratory distress syndrome"[MeSH Terms] OR ("respiratory"[All Fields] AND "distress"[All Fields] AND "syndrome"[All Fields]) OR "respiratory distress syndrome"[All Fields]) OR ("respiratory distress syndrome"[MeSH Terms] OR ("respiratory"[All Fields] AND "distress"[All Fields] AND "syndrome"[All Fields]) OR "respiratory distress syndrome"[All Fields] OR "ards"[All Fields]))) NOT ("animals"[MeSH Terms:noexp] OR "animal"[All Fields])) AND ((clinicalstudy[Filter] OR clinicaltrial[Filter] OR clinicaltrialphasei[Filter] OR clinicaltrialphaseii[Filter] OR clinicaltrialphaseiii[Filter] OR clinicaltrialphaseiv[Filter] OR comparativestudy[Filter] OR controlledclinicaltrial[Filter] OR correctedandrepublishedarticle[Filter] OR dataset[Filter] OR electronicsupplementarymaterials[Filter] OR englishabstract[Filter] OR randomizedcontrolledtrial[Filter]) AND (humans[Filter]) AND (english[Filter]) AND (2019:2022[pdat])) | 23,378 |
| 31 | ((SARS-CoV-2 OR COVID-19 OR Coronavirus Infections OR 2019-nCoV OR coronavirus disease 2019 OR Hospitalization) AND (Prevalence OR Risk Factors OR Risk Assessment OR Epidemiology OR Mortality)) AND (Coronary Artery Disease OR Cardiovascular Diseases OR Acute Coronary Syndrome OR Chronic Disease OR Disease Attributes OR Respiratory Distress Syndrome OR ARDS) NOT animal | Clinical Study, Clinical Trial, Clinical Trial, Phase I, Clinical Trial, Phase II, Clinical Trial, Phase III, Clinical Trial, Phase IV, Comparative Study, Controlled Clinical Trial, Corrected and Republished Article, Dataset, Electronic Supplementary Materials, English Abstract, Practice Guideline, Randomized Controlled Trial, Humans, English, from 2019 - 2022 | ((("sars cov 2"[MeSH Terms] OR "sars cov 2"[All Fields] OR "sars cov 2"[All Fields] OR ("covid 19"[All Fields] OR "covid 19"[MeSH Terms] OR "covid 19 vaccines"[All Fields] OR "covid 19 vaccines"[MeSH Terms] OR "covid 19 serotherapy"[All Fields] OR "covid 19 serotherapy"[Supplementary Concept] OR "covid 19 nucleic acid testing"[All Fields] OR "covid 19 nucleic acid testing"[MeSH Terms] OR "covid 19 serological testing"[All Fields] OR "covid 19 serological testing"[MeSH Terms] OR "covid 19 testing"[All Fields] OR "covid 19 testing"[MeSH Terms] OR "sars cov 2"[All Fields] OR "sars cov 2"[MeSH Terms] OR "severe acute respiratory syndrome coronavirus 2"[All Fields] OR "ncov"[All Fields] OR "2019 ncov"[All Fields] OR (("coronavirus"[MeSH Terms] OR "coronavirus"[All Fields] OR "cov"[All Fields]) AND 2019/11/01:3000/12/31[Date - Publication])) OR ("coronavirus infections"[MeSH Terms] OR ("coronavirus"[All Fields] AND "infections"[All Fields]) OR "coronavirus infections"[All Fields]) OR ("sars cov 2"[MeSH Terms] OR "sars cov 2"[All Fields] OR "2019 ncov"[All Fields]) OR ("covid 19"[MeSH Terms] OR "covid 19"[All Fields] OR "coronavirus disease 2019"[All Fields]) OR ("hospital s"[All Fields] OR "hospitalisation"[All Fields] OR "hospitalization"[MeSH Terms] OR "hospitalization"[All Fields] OR "hospitalising"[All Fields] OR "hospitality"[All Fields] OR "hospitalisations"[All Fields] OR "hospitalised"[All Fields] OR "hospitalizations"[All Fields] OR "hospitalized"[All Fields] OR "hospitalize"[All Fields] OR "hospitalizing"[All Fields] OR "hospitals"[MeSH Terms] OR "hospitals"[All Fields] OR "hospital"[All Fields])) AND ("epidemiology"[MeSH Subheading] OR "epidemiology"[All Fields] OR "prevalence"[All Fields] OR "prevalence"[MeSH Terms] OR "prevalance"[All Fields] OR "prevalences"[All Fields] OR "prevalence s"[All Fields] OR "prevalent"[All Fields] OR "prevalently"[All Fields] OR "prevalents"[All Fields] OR ("risk factors"[MeSH Terms] OR ("risk"[All Fields] AND "factors"[All Fields]) OR "risk factors"[All Fields]) OR ("risk assessment"[MeSH Terms] OR ("risk"[All Fields] AND "assessment"[All Fields]) OR "risk assessment"[All Fields]) OR ("epidemiologies"[All Fields] OR "epidemiology"[MeSH Subheading] OR "epidemiology"[All Fields] OR "epidemiology"[MeSH Terms] OR "epidemiology s"[All Fields]) OR ("mortality"[MeSH Terms] OR "mortality"[All Fields] OR "mortalities"[All Fields] OR "mortality"[MeSH Subheading])) AND ("coronary artery disease"[MeSH Terms] OR ("coronary"[All Fields] AND "artery"[All Fields] AND "disease"[All Fields]) OR "coronary artery disease"[All Fields] OR ("cardiovascular diseases"[MeSH Terms] OR ("cardiovascular"[All Fields] AND "diseases"[All Fields]) OR "cardiovascular diseases"[All Fields]) OR ("acute coronary syndrome"[MeSH Terms] OR ("acute"[All Fields] AND "coronary"[All Fields] AND "syndrome"[All Fields]) OR "acute coronary syndrome"[All Fields]) OR ("chronic disease"[MeSH Terms] OR ("chronic"[All Fields] AND "disease"[All Fields]) OR "chronic disease"[All Fields]) OR ("disease attributes"[MeSH Terms] OR ("disease"[All Fields] AND "attributes"[All Fields]) OR "disease attributes"[All Fields]) OR ("respiratory distress syndrome"[MeSH Terms] OR ("respiratory"[All Fields] AND "distress"[All Fields] AND "syndrome"[All Fields]) OR "respiratory distress syndrome"[All Fields]) OR ("respiratory distress syndrome"[MeSH Terms] OR ("respiratory"[All Fields] AND "distress"[All Fields] AND "syndrome"[All Fields]) OR "respiratory distress syndrome"[All Fields] OR "ards"[All Fields]))) NOT ("animals"[MeSH Terms:noexp] OR "animal"[All Fields])) AND ((clinicalstudy[Filter] OR clinicaltrial[Filter] OR clinicaltrialphasei[Filter] OR clinicaltrialphaseii[Filter] OR clinicaltrialphaseiii[Filter] OR clinicaltrialphaseiv[Filter] OR comparativestudy[Filter] OR controlledclinicaltrial[Filter] OR correctedandrepublishedarticle[Filter] OR dataset[Filter] OR electronicsupplementarymaterials[Filter] OR englishabstract[Filter] OR practiceguideline[Filter] OR randomizedcontrolledtrial[Filter]) AND (humans[Filter]) AND (english[Filter]) AND (2019:2022[pdat])) | 23,830 |
| 32 | ((SARS-CoV-2 OR COVID-19 OR Coronavirus Infections OR 2019-nCoV OR coronavirus disease 2019 OR Hospitalization) AND (Prevalence OR Risk Factors OR Risk Assessment OR Epidemiology OR Mortality)) AND (Coronary Artery Disease OR Cardiovascular Diseases OR Acute Coronary Syndrome OR Chronic Disease OR Disease Attributes OR Respiratory Distress Syndrome OR ARDS) NOT animal | Clinical Study, Clinical Trial, Clinical Trial, Phase I, Clinical Trial, Phase II, Clinical Trial, Phase III, Clinical Trial, Phase IV, Comparative Study, Controlled Clinical Trial, Corrected and Republished Article, Dataset, Electronic Supplementary Materials, English Abstract, Evaluation Study, Practice Guideline, Randomized Controlled Trial, Humans, English, from 2019 - 2022 | ((("sars cov 2"[MeSH Terms] OR "sars cov 2"[All Fields] OR "sars cov 2"[All Fields] OR ("covid 19"[All Fields] OR "covid 19"[MeSH Terms] OR "covid 19 vaccines"[All Fields] OR "covid 19 vaccines"[MeSH Terms] OR "covid 19 serotherapy"[All Fields] OR "covid 19 serotherapy"[Supplementary Concept] OR "covid 19 nucleic acid testing"[All Fields] OR "covid 19 nucleic acid testing"[MeSH Terms] OR "covid 19 serological testing"[All Fields] OR "covid 19 serological testing"[MeSH Terms] OR "covid 19 testing"[All Fields] OR "covid 19 testing"[MeSH Terms] OR "sars cov 2"[All Fields] OR "sars cov 2"[MeSH Terms] OR "severe acute respiratory syndrome coronavirus 2"[All Fields] OR "ncov"[All Fields] OR "2019 ncov"[All Fields] OR (("coronavirus"[MeSH Terms] OR "coronavirus"[All Fields] OR "cov"[All Fields]) AND 2019/11/01:3000/12/31[Date - Publication])) OR ("coronavirus infections"[MeSH Terms] OR ("coronavirus"[All Fields] AND "infections"[All Fields]) OR "coronavirus infections"[All Fields]) OR ("sars cov 2"[MeSH Terms] OR "sars cov 2"[All Fields] OR "2019 ncov"[All Fields]) OR ("covid 19"[MeSH Terms] OR "covid 19"[All Fields] OR "coronavirus disease 2019"[All Fields]) OR ("hospital s"[All Fields] OR "hospitalisation"[All Fields] OR "hospitalization"[MeSH Terms] OR "hospitalization"[All Fields] OR "hospitalising"[All Fields] OR "hospitality"[All Fields] OR "hospitalisations"[All Fields] OR "hospitalised"[All Fields] OR "hospitalizations"[All Fields] OR "hospitalized"[All Fields] OR "hospitalize"[All Fields] OR "hospitalizing"[All Fields] OR "hospitals"[MeSH Terms] OR "hospitals"[All Fields] OR "hospital"[All Fields])) AND ("epidemiology"[MeSH Subheading] OR "epidemiology"[All Fields] OR "prevalence"[All Fields] OR "prevalence"[MeSH Terms] OR "prevalance"[All Fields] OR "prevalences"[All Fields] OR "prevalence s"[All Fields] OR "prevalent"[All Fields] OR "prevalently"[All Fields] OR "prevalents"[All Fields] OR ("risk factors"[MeSH Terms] OR ("risk"[All Fields] AND "factors"[All Fields]) OR "risk factors"[All Fields]) OR ("risk assessment"[MeSH Terms] OR ("risk"[All Fields] AND "assessment"[All Fields]) OR "risk assessment"[All Fields]) OR ("epidemiologies"[All Fields] OR "epidemiology"[MeSH Subheading] OR "epidemiology"[All Fields] OR "epidemiology"[MeSH Terms] OR "epidemiology s"[All Fields]) OR ("mortality"[MeSH Terms] OR "mortality"[All Fields] OR "mortalities"[All Fields] OR "mortality"[MeSH Subheading])) AND ("coronary artery disease"[MeSH Terms] OR ("coronary"[All Fields] AND "artery"[All Fields] AND "disease"[All Fields]) OR "coronary artery disease"[All Fields] OR ("cardiovascular diseases"[MeSH Terms] OR ("cardiovascular"[All Fields] AND "diseases"[All Fields]) OR "cardiovascular diseases"[All Fields]) OR ("acute coronary syndrome"[MeSH Terms] OR ("acute"[All Fields] AND "coronary"[All Fields] AND "syndrome"[All Fields]) OR "acute coronary syndrome"[All Fields]) OR ("chronic disease"[MeSH Terms] OR ("chronic"[All Fields] AND "disease"[All Fields]) OR "chronic disease"[All Fields]) OR ("disease attributes"[MeSH Terms] OR ("disease"[All Fields] AND "attributes"[All Fields]) OR "disease attributes"[All Fields]) OR ("respiratory distress syndrome"[MeSH Terms] OR ("respiratory"[All Fields] AND "distress"[All Fields] AND "syndrome"[All Fields]) OR "respiratory distress syndrome"[All Fields]) OR ("respiratory distress syndrome"[MeSH Terms] OR ("respiratory"[All Fields] AND "distress"[All Fields] AND "syndrome"[All Fields]) OR "respiratory distress syndrome"[All Fields] OR "ards"[All Fields]))) NOT ("animals"[MeSH Terms:noexp] OR "animal"[All Fields])) AND ((clinicalstudy[Filter] OR clinicaltrial[Filter] OR clinicaltrialphasei[Filter] OR clinicaltrialphaseii[Filter] OR clinicaltrialphaseiii[Filter] OR clinicaltrialphaseiv[Filter] OR comparativestudy[Filter] OR controlledclinicaltrial[Filter] OR correctedandrepublishedarticle[Filter] OR dataset[Filter] OR electronicsupplementarymaterials[Filter] OR englishabstract[Filter] OR evaluationstudy[Filter] OR practiceguideline[Filter] OR randomizedcontrolledtrial[Filter]) AND (humans[Filter]) AND (english[Filter]) AND (2019:2022[pdat])) | 24,416 |
| 33 | ((SARS-CoV-2 OR COVID-19 OR Coronavirus Infections OR 2019-nCoV OR coronavirus disease 2019 OR Hospitalization) AND (Prevalence OR Risk Factors OR Risk Assessment OR Epidemiology OR Mortality)) AND (Coronary Artery Disease OR Cardiovascular Diseases OR Acute Coronary Syndrome OR Chronic Disease OR Disease Attributes OR Respiratory Distress Syndrome OR ARDS) NOT animal | Clinical Study, Clinical Trial, Clinical Trial, Phase I, Clinical Trial, Phase II, Clinical Trial, Phase III, Clinical Trial, Phase IV, Comparative Study, Controlled Clinical Trial, Corrected and Republished Article, Dataset, Electronic Supplementary Materials, English Abstract, Evaluation Study, Government Publication, Practice Guideline, Randomized Controlled Trial, Humans, English, from 2019 - 2022 | ((("sars cov 2"[MeSH Terms] OR "sars cov 2"[All Fields] OR "sars cov 2"[All Fields] OR ("covid 19"[All Fields] OR "covid 19"[MeSH Terms] OR "covid 19 vaccines"[All Fields] OR "covid 19 vaccines"[MeSH Terms] OR "covid 19 serotherapy"[All Fields] OR "covid 19 serotherapy"[Supplementary Concept] OR "covid 19 nucleic acid testing"[All Fields] OR "covid 19 nucleic acid testing"[MeSH Terms] OR "covid 19 serological testing"[All Fields] OR "covid 19 serological testing"[MeSH Terms] OR "covid 19 testing"[All Fields] OR "covid 19 testing"[MeSH Terms] OR "sars cov 2"[All Fields] OR "sars cov 2"[MeSH Terms] OR "severe acute respiratory syndrome coronavirus 2"[All Fields] OR "ncov"[All Fields] OR "2019 ncov"[All Fields] OR (("coronavirus"[MeSH Terms] OR "coronavirus"[All Fields] OR "cov"[All Fields]) AND 2019/11/01:3000/12/31[Date - Publication])) OR ("coronavirus infections"[MeSH Terms] OR ("coronavirus"[All Fields] AND "infections"[All Fields]) OR "coronavirus infections"[All Fields]) OR ("sars cov 2"[MeSH Terms] OR "sars cov 2"[All Fields] OR "2019 ncov"[All Fields]) OR ("covid 19"[MeSH Terms] OR "covid 19"[All Fields] OR "coronavirus disease 2019"[All Fields]) OR ("hospital s"[All Fields] OR "hospitalisation"[All Fields] OR "hospitalization"[MeSH Terms] OR "hospitalization"[All Fields] OR "hospitalising"[All Fields] OR "hospitality"[All Fields] OR "hospitalisations"[All Fields] OR "hospitalised"[All Fields] OR "hospitalizations"[All Fields] OR "hospitalized"[All Fields] OR "hospitalize"[All Fields] OR "hospitalizing"[All Fields] OR "hospitals"[MeSH Terms] OR "hospitals"[All Fields] OR "hospital"[All Fields])) AND ("epidemiology"[MeSH Subheading] OR "epidemiology"[All Fields] OR "prevalence"[All Fields] OR "prevalence"[MeSH Terms] OR "prevalance"[All Fields] OR "prevalences"[All Fields] OR "prevalence s"[All Fields] OR "prevalent"[All Fields] OR "prevalently"[All Fields] OR "prevalents"[All Fields] OR ("risk factors"[MeSH Terms] OR ("risk"[All Fields] AND "factors"[All Fields]) OR "risk factors"[All Fields]) OR ("risk assessment"[MeSH Terms] OR ("risk"[All Fields] AND "assessment"[All Fields]) OR "risk assessment"[All Fields]) OR ("epidemiologies"[All Fields] OR "epidemiology"[MeSH Subheading] OR "epidemiology"[All Fields] OR "epidemiology"[MeSH Terms] OR "epidemiology s"[All Fields]) OR ("mortality"[MeSH Terms] OR "mortality"[All Fields] OR "mortalities"[All Fields] OR "mortality"[MeSH Subheading])) AND ("coronary artery disease"[MeSH Terms] OR ("coronary"[All Fields] AND "artery"[All Fields] AND "disease"[All Fields]) OR "coronary artery disease"[All Fields] OR ("cardiovascular diseases"[MeSH Terms] OR ("cardiovascular"[All Fields] AND "diseases"[All Fields]) OR "cardiovascular diseases"[All Fields]) OR ("acute coronary syndrome"[MeSH Terms] OR ("acute"[All Fields] AND "coronary"[All Fields] AND "syndrome"[All Fields]) OR "acute coronary syndrome"[All Fields]) OR ("chronic disease"[MeSH Terms] OR ("chronic"[All Fields] AND "disease"[All Fields]) OR "chronic disease"[All Fields]) OR ("disease attributes"[MeSH Terms] OR ("disease"[All Fields] AND "attributes"[All Fields]) OR "disease attributes"[All Fields]) OR ("respiratory distress syndrome"[MeSH Terms] OR ("respiratory"[All Fields] AND "distress"[All Fields] AND "syndrome"[All Fields]) OR "respiratory distress syndrome"[All Fields]) OR ("respiratory distress syndrome"[MeSH Terms] OR ("respiratory"[All Fields] AND "distress"[All Fields] AND "syndrome"[All Fields]) OR "respiratory distress syndrome"[All Fields] OR "ards"[All Fields]))) NOT ("animals"[MeSH Terms:noexp] OR "animal"[All Fields])) AND ((clinicalstudy[Filter] OR clinicaltrial[Filter] OR clinicaltrialphasei[Filter] OR clinicaltrialphaseii[Filter] OR clinicaltrialphaseiii[Filter] OR clinicaltrialphaseiv[Filter] OR comparativestudy[Filter] OR controlledclinicaltrial[Filter] OR correctedandrepublishedarticle[Filter] OR dataset[Filter] OR electronicsupplementarymaterials[Filter] OR englishabstract[Filter] OR evaluationstudy[Filter] OR governmentpublication[Filter] OR practiceguideline[Filter] OR randomizedcontrolledtrial[Filter]) AND (humans[Filter]) AND (english[Filter]) AND (2019:2022[pdat])) | 24,416 |
| 34 | ((SARS-CoV-2 OR COVID-19 OR Coronavirus Infections OR 2019-nCoV OR coronavirus disease 2019 OR Hospitalization) AND (Prevalence OR Risk Factors OR Risk Assessment OR Epidemiology OR Mortality)) AND (Coronary Artery Disease OR Cardiovascular Diseases OR Acute Coronary Syndrome OR Chronic Disease OR Disease Attributes OR Respiratory Distress Syndrome OR ARDS) NOT animal | Clinical Study, Clinical Trial, Clinical Trial, Phase I, Clinical Trial, Phase II, Clinical Trial, Phase III, Clinical Trial, Phase IV, Comparative Study, Controlled Clinical Trial, Corrected and Republished Article, Dataset, Electronic Supplementary Materials, English Abstract, Evaluation Study, Government Publication, Guideline, Practice Guideline, Randomized Controlled Trial, Humans, English, from 2019 - 2022 | ((("sars cov 2"[MeSH Terms] OR "sars cov 2"[All Fields] OR "sars cov 2"[All Fields] OR ("covid 19"[All Fields] OR "covid 19"[MeSH Terms] OR "covid 19 vaccines"[All Fields] OR "covid 19 vaccines"[MeSH Terms] OR "covid 19 serotherapy"[All Fields] OR "covid 19 serotherapy"[Supplementary Concept] OR "covid 19 nucleic acid testing"[All Fields] OR "covid 19 nucleic acid testing"[MeSH Terms] OR "covid 19 serological testing"[All Fields] OR "covid 19 serological testing"[MeSH Terms] OR "covid 19 testing"[All Fields] OR "covid 19 testing"[MeSH Terms] OR "sars cov 2"[All Fields] OR "sars cov 2"[MeSH Terms] OR "severe acute respiratory syndrome coronavirus 2"[All Fields] OR "ncov"[All Fields] OR "2019 ncov"[All Fields] OR (("coronavirus"[MeSH Terms] OR "coronavirus"[All Fields] OR "cov"[All Fields]) AND 2019/11/01:3000/12/31[Date - Publication])) OR ("coronavirus infections"[MeSH Terms] OR ("coronavirus"[All Fields] AND "infections"[All Fields]) OR "coronavirus infections"[All Fields]) OR ("sars cov 2"[MeSH Terms] OR "sars cov 2"[All Fields] OR "2019 ncov"[All Fields]) OR ("covid 19"[MeSH Terms] OR "covid 19"[All Fields] OR "coronavirus disease 2019"[All Fields]) OR ("hospital s"[All Fields] OR "hospitalisation"[All Fields] OR "hospitalization"[MeSH Terms] OR "hospitalization"[All Fields] OR "hospitalising"[All Fields] OR "hospitality"[All Fields] OR "hospitalisations"[All Fields] OR "hospitalised"[All Fields] OR "hospitalizations"[All Fields] OR "hospitalized"[All Fields] OR "hospitalize"[All Fields] OR "hospitalizing"[All Fields] OR "hospitals"[MeSH Terms] OR "hospitals"[All Fields] OR "hospital"[All Fields])) AND ("epidemiology"[MeSH Subheading] OR "epidemiology"[All Fields] OR "prevalence"[All Fields] OR "prevalence"[MeSH Terms] OR "prevalance"[All Fields] OR "prevalences"[All Fields] OR "prevalence s"[All Fields] OR "prevalent"[All Fields] OR "prevalently"[All Fields] OR "prevalents"[All Fields] OR ("risk factors"[MeSH Terms] OR ("risk"[All Fields] AND "factors"[All Fields]) OR "risk factors"[All Fields]) OR ("risk assessment"[MeSH Terms] OR ("risk"[All Fields] AND "assessment"[All Fields]) OR "risk assessment"[All Fields]) OR ("epidemiologies"[All Fields] OR "epidemiology"[MeSH Subheading] OR "epidemiology"[All Fields] OR "epidemiology"[MeSH Terms] OR "epidemiology s"[All Fields]) OR ("mortality"[MeSH Terms] OR "mortality"[All Fields] OR "mortalities"[All Fields] OR "mortality"[MeSH Subheading])) AND ("coronary artery disease"[MeSH Terms] OR ("coronary"[All Fields] AND "artery"[All Fields] AND "disease"[All Fields]) OR "coronary artery disease"[All Fields] OR ("cardiovascular diseases"[MeSH Terms] OR ("cardiovascular"[All Fields] AND "diseases"[All Fields]) OR "cardiovascular diseases"[All Fields]) OR ("acute coronary syndrome"[MeSH Terms] OR ("acute"[All Fields] AND "coronary"[All Fields] AND "syndrome"[All Fields]) OR "acute coronary syndrome"[All Fields]) OR ("chronic disease"[MeSH Terms] OR ("chronic"[All Fields] AND "disease"[All Fields]) OR "chronic disease"[All Fields]) OR ("disease attributes"[MeSH Terms] OR ("disease"[All Fields] AND "attributes"[All Fields]) OR "disease attributes"[All Fields]) OR ("respiratory distress syndrome"[MeSH Terms] OR ("respiratory"[All Fields] AND "distress"[All Fields] AND "syndrome"[All Fields]) OR "respiratory distress syndrome"[All Fields]) OR ("respiratory distress syndrome"[MeSH Terms] OR ("respiratory"[All Fields] AND "distress"[All Fields] AND "syndrome"[All Fields]) OR "respiratory distress syndrome"[All Fields] OR "ards"[All Fields]))) NOT ("animals"[MeSH Terms:noexp] OR "animal"[All Fields])) AND ((clinicalstudy[Filter] OR clinicaltrial[Filter] OR clinicaltrialphasei[Filter] OR clinicaltrialphaseii[Filter] OR clinicaltrialphaseiii[Filter] OR clinicaltrialphaseiv[Filter] OR comparativestudy[Filter] OR controlledclinicaltrial[Filter] OR correctedandrepublishedarticle[Filter] OR dataset[Filter] OR electronicsupplementarymaterials[Filter] OR englishabstract[Filter] OR evaluationstudy[Filter] OR governmentpublication[Filter] OR guideline[Filter] OR practiceguideline[Filter] OR randomizedcontrolledtrial[Filter]) AND (humans[Filter]) AND (english[Filter]) AND (2019:2022[pdat])) | 24,429 |
| 35 | ((SARS-CoV-2 OR COVID-19 OR Coronavirus Infections OR 2019-nCoV OR coronavirus disease 2019 OR Hospitalization) AND (Prevalence OR Risk Factors OR Risk Assessment OR Epidemiology OR Mortality)) AND (Coronary Artery Disease OR Cardiovascular Diseases OR Acute Coronary Syndrome OR Chronic Disease OR Disease Attributes OR Respiratory Distress Syndrome OR ARDS) NOT animal | Clinical Study, Clinical Trial, Clinical Trial, Phase I, Clinical Trial, Phase II, Clinical Trial, Phase III, Clinical Trial, Phase IV, Comparative Study, Controlled Clinical Trial, Corrected and Republished Article, Dataset, Electronic Supplementary Materials, English Abstract, Evaluation Study, Government Publication, Guideline, Introductory Journal Article, Practice Guideline, Randomized Controlled Trial, Humans, English, from 2019 - 2022 | ((("sars cov 2"[MeSH Terms] OR "sars cov 2"[All Fields] OR "sars cov 2"[All Fields] OR ("covid 19"[All Fields] OR "covid 19"[MeSH Terms] OR "covid 19 vaccines"[All Fields] OR "covid 19 vaccines"[MeSH Terms] OR "covid 19 serotherapy"[All Fields] OR "covid 19 serotherapy"[Supplementary Concept] OR "covid 19 nucleic acid testing"[All Fields] OR "covid 19 nucleic acid testing"[MeSH Terms] OR "covid 19 serological testing"[All Fields] OR "covid 19 serological testing"[MeSH Terms] OR "covid 19 testing"[All Fields] OR "covid 19 testing"[MeSH Terms] OR "sars cov 2"[All Fields] OR "sars cov 2"[MeSH Terms] OR "severe acute respiratory syndrome coronavirus 2"[All Fields] OR "ncov"[All Fields] OR "2019 ncov"[All Fields] OR (("coronavirus"[MeSH Terms] OR "coronavirus"[All Fields] OR "cov"[All Fields]) AND 2019/11/01:3000/12/31[Date - Publication])) OR ("coronavirus infections"[MeSH Terms] OR ("coronavirus"[All Fields] AND "infections"[All Fields]) OR "coronavirus infections"[All Fields]) OR ("sars cov 2"[MeSH Terms] OR "sars cov 2"[All Fields] OR "2019 ncov"[All Fields]) OR ("covid 19"[MeSH Terms] OR "covid 19"[All Fields] OR "coronavirus disease 2019"[All Fields]) OR ("hospital s"[All Fields] OR "hospitalisation"[All Fields] OR "hospitalization"[MeSH Terms] OR "hospitalization"[All Fields] OR "hospitalising"[All Fields] OR "hospitality"[All Fields] OR "hospitalisations"[All Fields] OR "hospitalised"[All Fields] OR "hospitalizations"[All Fields] OR "hospitalized"[All Fields] OR "hospitalize"[All Fields] OR "hospitalizing"[All Fields] OR "hospitals"[MeSH Terms] OR "hospitals"[All Fields] OR "hospital"[All Fields])) AND ("epidemiology"[MeSH Subheading] OR "epidemiology"[All Fields] OR "prevalence"[All Fields] OR "prevalence"[MeSH Terms] OR "prevalance"[All Fields] OR "prevalences"[All Fields] OR "prevalence s"[All Fields] OR "prevalent"[All Fields] OR "prevalently"[All Fields] OR "prevalents"[All Fields] OR ("risk factors"[MeSH Terms] OR ("risk"[All Fields] AND "factors"[All Fields]) OR "risk factors"[All Fields]) OR ("risk assessment"[MeSH Terms] OR ("risk"[All Fields] AND "assessment"[All Fields]) OR "risk assessment"[All Fields]) OR ("epidemiologies"[All Fields] OR "epidemiology"[MeSH Subheading] OR "epidemiology"[All Fields] OR "epidemiology"[MeSH Terms] OR "epidemiology s"[All Fields]) OR ("mortality"[MeSH Terms] OR "mortality"[All Fields] OR "mortalities"[All Fields] OR "mortality"[MeSH Subheading])) AND ("coronary artery disease"[MeSH Terms] OR ("coronary"[All Fields] AND "artery"[All Fields] AND "disease"[All Fields]) OR "coronary artery disease"[All Fields] OR ("cardiovascular diseases"[MeSH Terms] OR ("cardiovascular"[All Fields] AND "diseases"[All Fields]) OR "cardiovascular diseases"[All Fields]) OR ("acute coronary syndrome"[MeSH Terms] OR ("acute"[All Fields] AND "coronary"[All Fields] AND "syndrome"[All Fields]) OR "acute coronary syndrome"[All Fields]) OR ("chronic disease"[MeSH Terms] OR ("chronic"[All Fields] AND "disease"[All Fields]) OR "chronic disease"[All Fields]) OR ("disease attributes"[MeSH Terms] OR ("disease"[All Fields] AND "attributes"[All Fields]) OR "disease attributes"[All Fields]) OR ("respiratory distress syndrome"[MeSH Terms] OR ("respiratory"[All Fields] AND "distress"[All Fields] AND "syndrome"[All Fields]) OR "respiratory distress syndrome"[All Fields]) OR ("respiratory distress syndrome"[MeSH Terms] OR ("respiratory"[All Fields] AND "distress"[All Fields] AND "syndrome"[All Fields]) OR "respiratory distress syndrome"[All Fields] OR "ards"[All Fields]))) NOT ("animals"[MeSH Terms:noexp] OR "animal"[All Fields])) AND ((clinicalstudy[Filter] OR clinicaltrial[Filter] OR clinicaltrialphasei[Filter] OR clinicaltrialphaseii[Filter] OR clinicaltrialphaseiii[Filter] OR clinicaltrialphaseiv[Filter] OR comparativestudy[Filter] OR controlledclinicaltrial[Filter] OR correctedandrepublishedarticle[Filter] OR dataset[Filter] OR electronicsupplementarymaterials[Filter] OR englishabstract[Filter] OR evaluationstudy[Filter] OR governmentpublication[Filter] OR guideline[Filter] OR introductoryjournalarticle[Filter] OR practiceguideline[Filter] OR randomizedcontrolledtrial[Filter]) AND (humans[Filter]) AND (english[Filter]) AND (2019:2022[pdat])) | 24,536 |
| 36 | ((SARS-CoV-2 OR COVID-19 OR Coronavirus Infections OR 2019-nCoV OR coronavirus disease 2019 OR Hospitalization) AND (Prevalence OR Risk Factors OR Risk Assessment OR Epidemiology OR Mortality)) AND (Coronary Artery Disease OR Cardiovascular Diseases OR Acute Coronary Syndrome OR Chronic Disease OR Disease Attributes OR Respiratory Distress Syndrome OR ARDS) NOT animal | Clinical Study, Clinical Trial, Clinical Trial, Phase I, Clinical Trial, Phase II, Clinical Trial, Phase III, Clinical Trial, Phase IV, Comparative Study, Controlled Clinical Trial, Corrected and Republished Article, Dataset, Electronic Supplementary Materials, English Abstract, Evaluation Study, Government Publication, Guideline, Introductory Journal Article, Multicenter Study, Practice Guideline, Randomized Controlled Trial, Humans, English, from 2019 - 2022 | ((("sars cov 2"[MeSH Terms] OR "sars cov 2"[All Fields] OR "sars cov 2"[All Fields] OR ("covid 19"[All Fields] OR "covid 19"[MeSH Terms] OR "covid 19 vaccines"[All Fields] OR "covid 19 vaccines"[MeSH Terms] OR "covid 19 serotherapy"[All Fields] OR "covid 19 serotherapy"[Supplementary Concept] OR "covid 19 nucleic acid testing"[All Fields] OR "covid 19 nucleic acid testing"[MeSH Terms] OR "covid 19 serological testing"[All Fields] OR "covid 19 serological testing"[MeSH Terms] OR "covid 19 testing"[All Fields] OR "covid 19 testing"[MeSH Terms] OR "sars cov 2"[All Fields] OR "sars cov 2"[MeSH Terms] OR "severe acute respiratory syndrome coronavirus 2"[All Fields] OR "ncov"[All Fields] OR "2019 ncov"[All Fields] OR (("coronavirus"[MeSH Terms] OR "coronavirus"[All Fields] OR "cov"[All Fields]) AND 2019/11/01:3000/12/31[Date - Publication])) OR ("coronavirus infections"[MeSH Terms] OR ("coronavirus"[All Fields] AND "infections"[All Fields]) OR "coronavirus infections"[All Fields]) OR ("sars cov 2"[MeSH Terms] OR "sars cov 2"[All Fields] OR "2019 ncov"[All Fields]) OR ("covid 19"[MeSH Terms] OR "covid 19"[All Fields] OR "coronavirus disease 2019"[All Fields]) OR ("hospital s"[All Fields] OR "hospitalisation"[All Fields] OR "hospitalization"[MeSH Terms] OR "hospitalization"[All Fields] OR "hospitalising"[All Fields] OR "hospitality"[All Fields] OR "hospitalisations"[All Fields] OR "hospitalised"[All Fields] OR "hospitalizations"[All Fields] OR "hospitalized"[All Fields] OR "hospitalize"[All Fields] OR "hospitalizing"[All Fields] OR "hospitals"[MeSH Terms] OR "hospitals"[All Fields] OR "hospital"[All Fields])) AND ("epidemiology"[MeSH Subheading] OR "epidemiology"[All Fields] OR "prevalence"[All Fields] OR "prevalence"[MeSH Terms] OR "prevalance"[All Fields] OR "prevalences"[All Fields] OR "prevalence s"[All Fields] OR "prevalent"[All Fields] OR "prevalently"[All Fields] OR "prevalents"[All Fields] OR ("risk factors"[MeSH Terms] OR ("risk"[All Fields] AND "factors"[All Fields]) OR "risk factors"[All Fields]) OR ("risk assessment"[MeSH Terms] OR ("risk"[All Fields] AND "assessment"[All Fields]) OR "risk assessment"[All Fields]) OR ("epidemiologies"[All Fields] OR "epidemiology"[MeSH Subheading] OR "epidemiology"[All Fields] OR "epidemiology"[MeSH Terms] OR "epidemiology s"[All Fields]) OR ("mortality"[MeSH Terms] OR "mortality"[All Fields] OR "mortalities"[All Fields] OR "mortality"[MeSH Subheading])) AND ("coronary artery disease"[MeSH Terms] OR ("coronary"[All Fields] AND "artery"[All Fields] AND "disease"[All Fields]) OR "coronary artery disease"[All Fields] OR ("cardiovascular diseases"[MeSH Terms] OR ("cardiovascular"[All Fields] AND "diseases"[All Fields]) OR "cardiovascular diseases"[All Fields]) OR ("acute coronary syndrome"[MeSH Terms] OR ("acute"[All Fields] AND "coronary"[All Fields] AND "syndrome"[All Fields]) OR "acute coronary syndrome"[All Fields]) OR ("chronic disease"[MeSH Terms] OR ("chronic"[All Fields] AND "disease"[All Fields]) OR "chronic disease"[All Fields]) OR ("disease attributes"[MeSH Terms] OR ("disease"[All Fields] AND "attributes"[All Fields]) OR "disease attributes"[All Fields]) OR ("respiratory distress syndrome"[MeSH Terms] OR ("respiratory"[All Fields] AND "distress"[All Fields] AND "syndrome"[All Fields]) OR "respiratory distress syndrome"[All Fields]) OR ("respiratory distress syndrome"[MeSH Terms] OR ("respiratory"[All Fields] AND "distress"[All Fields] AND "syndrome"[All Fields]) OR "respiratory distress syndrome"[All Fields] OR "ards"[All Fields]))) NOT ("animals"[MeSH Terms:noexp] OR "animal"[All Fields])) AND ((clinicalstudy[Filter] OR clinicaltrial[Filter] OR clinicaltrialphasei[Filter] OR clinicaltrialphaseii[Filter] OR clinicaltrialphaseiii[Filter] OR clinicaltrialphaseiv[Filter] OR comparativestudy[Filter] OR controlledclinicaltrial[Filter] OR correctedandrepublishedarticle[Filter] OR dataset[Filter] OR electronicsupplementarymaterials[Filter] OR englishabstract[Filter] OR evaluationstudy[Filter] OR governmentpublication[Filter] OR guideline[Filter] OR introductoryjournalarticle[Filter] OR multicenterstudy[Filter] OR practiceguideline[Filter] OR randomizedcontrolledtrial[Filter]) AND (humans[Filter]) AND (english[Filter]) AND (2019:2022[pdat])) | 31,038 |
| 37 | ((SARS-CoV-2 OR COVID-19 OR Coronavirus Infections OR 2019-nCoV OR coronavirus disease 2019 OR Hospitalization) AND (Prevalence OR Risk Factors OR Risk Assessment OR Epidemiology OR Mortality)) AND (Coronary Artery Disease OR Cardiovascular Diseases OR Acute Coronary Syndrome OR Chronic Disease OR Disease Attributes OR Respiratory Distress Syndrome OR ARDS) NOT animal | Clinical Study, Clinical Trial, Clinical Trial, Phase I, Clinical Trial, Phase II, Clinical Trial, Phase III, Clinical Trial, Phase IV, Comparative Study, Controlled Clinical Trial, Corrected and Republished Article, Dataset, Electronic Supplementary Materials, English Abstract, Evaluation Study, Government Publication, Guideline, Introductory Journal Article, Multicenter Study, Practice Guideline, Randomized Controlled Trial, Technical Report, Humans, English, from 2019 - 2022 | ((("sars cov 2"[MeSH Terms] OR "sars cov 2"[All Fields] OR "sars cov 2"[All Fields] OR ("covid 19"[All Fields] OR "covid 19"[MeSH Terms] OR "covid 19 vaccines"[All Fields] OR "covid 19 vaccines"[MeSH Terms] OR "covid 19 serotherapy"[All Fields] OR "covid 19 serotherapy"[Supplementary Concept] OR "covid 19 nucleic acid testing"[All Fields] OR "covid 19 nucleic acid testing"[MeSH Terms] OR "covid 19 serological testing"[All Fields] OR "covid 19 serological testing"[MeSH Terms] OR "covid 19 testing"[All Fields] OR "covid 19 testing"[MeSH Terms] OR "sars cov 2"[All Fields] OR "sars cov 2"[MeSH Terms] OR "severe acute respiratory syndrome coronavirus 2"[All Fields] OR "ncov"[All Fields] OR "2019 ncov"[All Fields] OR (("coronavirus"[MeSH Terms] OR "coronavirus"[All Fields] OR "cov"[All Fields]) AND 2019/11/01:3000/12/31[Date - Publication])) OR ("coronavirus infections"[MeSH Terms] OR ("coronavirus"[All Fields] AND "infections"[All Fields]) OR "coronavirus infections"[All Fields]) OR ("sars cov 2"[MeSH Terms] OR "sars cov 2"[All Fields] OR "2019 ncov"[All Fields]) OR ("covid 19"[MeSH Terms] OR "covid 19"[All Fields] OR "coronavirus disease 2019"[All Fields]) OR ("hospital s"[All Fields] OR "hospitalisation"[All Fields] OR "hospitalization"[MeSH Terms] OR "hospitalization"[All Fields] OR "hospitalising"[All Fields] OR "hospitality"[All Fields] OR "hospitalisations"[All Fields] OR "hospitalised"[All Fields] OR "hospitalizations"[All Fields] OR "hospitalized"[All Fields] OR "hospitalize"[All Fields] OR "hospitalizing"[All Fields] OR "hospitals"[MeSH Terms] OR "hospitals"[All Fields] OR "hospital"[All Fields])) AND ("epidemiology"[MeSH Subheading] OR "epidemiology"[All Fields] OR "prevalence"[All Fields] OR "prevalence"[MeSH Terms] OR "prevalance"[All Fields] OR "prevalences"[All Fields] OR "prevalence s"[All Fields] OR "prevalent"[All Fields] OR "prevalently"[All Fields] OR "prevalents"[All Fields] OR ("risk factors"[MeSH Terms] OR ("risk"[All Fields] AND "factors"[All Fields]) OR "risk factors"[All Fields]) OR ("risk assessment"[MeSH Terms] OR ("risk"[All Fields] AND "assessment"[All Fields]) OR "risk assessment"[All Fields]) OR ("epidemiologies"[All Fields] OR "epidemiology"[MeSH Subheading] OR "epidemiology"[All Fields] OR "epidemiology"[MeSH Terms] OR "epidemiology s"[All Fields]) OR ("mortality"[MeSH Terms] OR "mortality"[All Fields] OR "mortalities"[All Fields] OR "mortality"[MeSH Subheading])) AND ("coronary artery disease"[MeSH Terms] OR ("coronary"[All Fields] AND "artery"[All Fields] AND "disease"[All Fields]) OR "coronary artery disease"[All Fields] OR ("cardiovascular diseases"[MeSH Terms] OR ("cardiovascular"[All Fields] AND "diseases"[All Fields]) OR "cardiovascular diseases"[All Fields]) OR ("acute coronary syndrome"[MeSH Terms] OR ("acute"[All Fields] AND "coronary"[All Fields] AND "syndrome"[All Fields]) OR "acute coronary syndrome"[All Fields]) OR ("chronic disease"[MeSH Terms] OR ("chronic"[All Fields] AND "disease"[All Fields]) OR "chronic disease"[All Fields]) OR ("disease attributes"[MeSH Terms] OR ("disease"[All Fields] AND "attributes"[All Fields]) OR "disease attributes"[All Fields]) OR ("respiratory distress syndrome"[MeSH Terms] OR ("respiratory"[All Fields] AND "distress"[All Fields] AND "syndrome"[All Fields]) OR "respiratory distress syndrome"[All Fields]) OR ("respiratory distress syndrome"[MeSH Terms] OR ("respiratory"[All Fields] AND "distress"[All Fields] AND "syndrome"[All Fields]) OR "respiratory distress syndrome"[All Fields] OR "ards"[All Fields]))) NOT ("animals"[MeSH Terms:noexp] OR "animal"[All Fields])) AND ((clinicalstudy[Filter] OR clinicaltrial[Filter] OR clinicaltrialphasei[Filter] OR clinicaltrialphaseii[Filter] OR clinicaltrialphaseiii[Filter] OR clinicaltrialphaseiv[Filter] OR comparativestudy[Filter] OR controlledclinicaltrial[Filter] OR correctedandrepublishedarticle[Filter] OR dataset[Filter] OR electronicsupplementarymaterials[Filter] OR englishabstract[Filter] OR evaluationstudy[Filter] OR governmentpublication[Filter] OR guideline[Filter] OR introductoryjournalarticle[Filter] OR multicenterstudy[Filter] OR practiceguideline[Filter] OR randomizedcontrolledtrial[Filter] OR technicalreport[Filter]) AND (humans[Filter]) AND (english[Filter]) AND (2019:2022[pdat])) | 31,040 |
| 38 | ((SARS-CoV-2 OR COVID-19 OR Coronavirus Infections OR 2019-nCoV OR coronavirus disease 2019 OR Hospitalization) AND (Prevalence OR Risk Factors OR Risk Assessment OR Epidemiology OR Mortality)) AND (Coronary Artery Disease OR Cardiovascular Diseases OR Acute Coronary Syndrome OR Chronic Disease OR Disease Attributes OR Respiratory Distress Syndrome OR ARDS) NOT animal | Clinical Study, Clinical Trial, Clinical Trial, Phase I, Clinical Trial, Phase II, Clinical Trial, Phase III, Clinical Trial, Phase IV, Comparative Study, Controlled Clinical Trial, Corrected and Republished Article, Dataset, Electronic Supplementary Materials, English Abstract, Evaluation Study, Government Publication, Guideline, Introductory Journal Article, Multicenter Study, Observational Study, Practice Guideline, Randomized Controlled Trial, Technical Report, Humans, English, from 2019 - 2022 | ((("sars cov 2"[MeSH Terms] OR "sars cov 2"[All Fields] OR "sars cov 2"[All Fields] OR ("covid 19"[All Fields] OR "covid 19"[MeSH Terms] OR "covid 19 vaccines"[All Fields] OR "covid 19 vaccines"[MeSH Terms] OR "covid 19 serotherapy"[All Fields] OR "covid 19 serotherapy"[Supplementary Concept] OR "covid 19 nucleic acid testing"[All Fields] OR "covid 19 nucleic acid testing"[MeSH Terms] OR "covid 19 serological testing"[All Fields] OR "covid 19 serological testing"[MeSH Terms] OR "covid 19 testing"[All Fields] OR "covid 19 testing"[MeSH Terms] OR "sars cov 2"[All Fields] OR "sars cov 2"[MeSH Terms] OR "severe acute respiratory syndrome coronavirus 2"[All Fields] OR "ncov"[All Fields] OR "2019 ncov"[All Fields] OR (("coronavirus"[MeSH Terms] OR "coronavirus"[All Fields] OR "cov"[All Fields]) AND 2019/11/01:3000/12/31[Date - Publication])) OR ("coronavirus infections"[MeSH Terms] OR ("coronavirus"[All Fields] AND "infections"[All Fields]) OR "coronavirus infections"[All Fields]) OR ("sars cov 2"[MeSH Terms] OR "sars cov 2"[All Fields] OR "2019 ncov"[All Fields]) OR ("covid 19"[MeSH Terms] OR "covid 19"[All Fields] OR "coronavirus disease 2019"[All Fields]) OR ("hospital s"[All Fields] OR "hospitalisation"[All Fields] OR "hospitalization"[MeSH Terms] OR "hospitalization"[All Fields] OR "hospitalising"[All Fields] OR "hospitality"[All Fields] OR "hospitalisations"[All Fields] OR "hospitalised"[All Fields] OR "hospitalizations"[All Fields] OR "hospitalized"[All Fields] OR "hospitalize"[All Fields] OR "hospitalizing"[All Fields] OR "hospitals"[MeSH Terms] OR "hospitals"[All Fields] OR "hospital"[All Fields])) AND ("epidemiology"[MeSH Subheading] OR "epidemiology"[All Fields] OR "prevalence"[All Fields] OR "prevalence"[MeSH Terms] OR "prevalance"[All Fields] OR "prevalences"[All Fields] OR "prevalence s"[All Fields] OR "prevalent"[All Fields] OR "prevalently"[All Fields] OR "prevalents"[All Fields] OR ("risk factors"[MeSH Terms] OR ("risk"[All Fields] AND "factors"[All Fields]) OR "risk factors"[All Fields]) OR ("risk assessment"[MeSH Terms] OR ("risk"[All Fields] AND "assessment"[All Fields]) OR "risk assessment"[All Fields]) OR ("epidemiologies"[All Fields] OR "epidemiology"[MeSH Subheading] OR "epidemiology"[All Fields] OR "epidemiology"[MeSH Terms] OR "epidemiology s"[All Fields]) OR ("mortality"[MeSH Terms] OR "mortality"[All Fields] OR "mortalities"[All Fields] OR "mortality"[MeSH Subheading])) AND ("coronary artery disease"[MeSH Terms] OR ("coronary"[All Fields] AND "artery"[All Fields] AND "disease"[All Fields]) OR "coronary artery disease"[All Fields] OR ("cardiovascular diseases"[MeSH Terms] OR ("cardiovascular"[All Fields] AND "diseases"[All Fields]) OR "cardiovascular diseases"[All Fields]) OR ("acute coronary syndrome"[MeSH Terms] OR ("acute"[All Fields] AND "coronary"[All Fields] AND "syndrome"[All Fields]) OR "acute coronary syndrome"[All Fields]) OR ("chronic disease"[MeSH Terms] OR ("chronic"[All Fields] AND "disease"[All Fields]) OR "chronic disease"[All Fields]) OR ("disease attributes"[MeSH Terms] OR ("disease"[All Fields] AND "attributes"[All Fields]) OR "disease attributes"[All Fields]) OR ("respiratory distress syndrome"[MeSH Terms] OR ("respiratory"[All Fields] AND "distress"[All Fields] AND "syndrome"[All Fields]) OR "respiratory distress syndrome"[All Fields]) OR ("respiratory distress syndrome"[MeSH Terms] OR ("respiratory"[All Fields] AND "distress"[All Fields] AND "syndrome"[All Fields]) OR "respiratory distress syndrome"[All Fields] OR "ards"[All Fields]))) NOT ("animals"[MeSH Terms:noexp] OR "animal"[All Fields])) AND ((clinicalstudy[Filter] OR clinicaltrial[Filter] OR clinicaltrialphasei[Filter] OR clinicaltrialphaseii[Filter] OR clinicaltrialphaseiii[Filter] OR clinicaltrialphaseiv[Filter] OR comparativestudy[Filter] OR controlledclinicaltrial[Filter] OR correctedandrepublishedarticle[Filter] OR dataset[Filter] OR electronicsupplementarymaterials[Filter] OR englishabstract[Filter] OR evaluationstudy[Filter] OR governmentpublication[Filter] OR guideline[Filter] OR introductoryjournalarticle[Filter] OR multicenterstudy[Filter] OR observationalstudy[Filter] OR practiceguideline[Filter] OR randomizedcontrolledtrial[Filter] OR technicalreport[Filter]) AND (humans[Filter]) AND (english[Filter]) AND (2019:2022[pdat])) | 31,040 |
| 39 | ((SARS-CoV-2 OR COVID-19 OR Coronavirus Infections OR 2019-nCoV OR coronavirus disease 2019 OR Hospitalization) AND (Prevalence OR Risk Factors OR Risk Assessment OR Epidemiology OR Mortality)) AND (Coronary Artery Disease OR Cardiovascular Diseases OR Acute Coronary Syndrome OR Chronic Disease OR Disease Attributes OR Respiratory Distress Syndrome OR ARDS) NOT animal | Clinical Study, Clinical Trial, Clinical Trial, Phase I, Clinical Trial, Phase II, Clinical Trial, Phase III, Clinical Trial, Phase IV, Comparative Study, Controlled Clinical Trial, Corrected and Republished Article, Dataset, Electronic Supplementary Materials, English Abstract, Evaluation Study, Government Publication, Guideline, Introductory Journal Article, Multicenter Study, Observational Study, Practice Guideline, Randomized Controlled Trial, Technical Report, Validation Study, Humans, English, from 2019 - 2022 | ((("sars cov 2"[MeSH Terms] OR "sars cov 2"[All Fields] OR "sars cov 2"[All Fields] OR ("covid 19"[All Fields] OR "covid 19"[MeSH Terms] OR "covid 19 vaccines"[All Fields] OR "covid 19 vaccines"[MeSH Terms] OR "covid 19 serotherapy"[All Fields] OR "covid 19 serotherapy"[Supplementary Concept] OR "covid 19 nucleic acid testing"[All Fields] OR "covid 19 nucleic acid testing"[MeSH Terms] OR "covid 19 serological testing"[All Fields] OR "covid 19 serological testing"[MeSH Terms] OR "covid 19 testing"[All Fields] OR "covid 19 testing"[MeSH Terms] OR "sars cov 2"[All Fields] OR "sars cov 2"[MeSH Terms] OR "severe acute respiratory syndrome coronavirus 2"[All Fields] OR "ncov"[All Fields] OR "2019 ncov"[All Fields] OR (("coronavirus"[MeSH Terms] OR "coronavirus"[All Fields] OR "cov"[All Fields]) AND 2019/11/01:3000/12/31[Date - Publication])) OR ("coronavirus infections"[MeSH Terms] OR ("coronavirus"[All Fields] AND "infections"[All Fields]) OR "coronavirus infections"[All Fields]) OR ("sars cov 2"[MeSH Terms] OR "sars cov 2"[All Fields] OR "2019 ncov"[All Fields]) OR ("covid 19"[MeSH Terms] OR "covid 19"[All Fields] OR "coronavirus disease 2019"[All Fields]) OR ("hospital s"[All Fields] OR "hospitalisation"[All Fields] OR "hospitalization"[MeSH Terms] OR "hospitalization"[All Fields] OR "hospitalising"[All Fields] OR "hospitality"[All Fields] OR "hospitalisations"[All Fields] OR "hospitalised"[All Fields] OR "hospitalizations"[All Fields] OR "hospitalized"[All Fields] OR "hospitalize"[All Fields] OR "hospitalizing"[All Fields] OR "hospitals"[MeSH Terms] OR "hospitals"[All Fields] OR "hospital"[All Fields])) AND ("epidemiology"[MeSH Subheading] OR "epidemiology"[All Fields] OR "prevalence"[All Fields] OR "prevalence"[MeSH Terms] OR "prevalance"[All Fields] OR "prevalences"[All Fields] OR "prevalence s"[All Fields] OR "prevalent"[All Fields] OR "prevalently"[All Fields] OR "prevalents"[All Fields] OR ("risk factors"[MeSH Terms] OR ("risk"[All Fields] AND "factors"[All Fields]) OR "risk factors"[All Fields]) OR ("risk assessment"[MeSH Terms] OR ("risk"[All Fields] AND "assessment"[All Fields]) OR "risk assessment"[All Fields]) OR ("epidemiologies"[All Fields] OR "epidemiology"[MeSH Subheading] OR "epidemiology"[All Fields] OR "epidemiology"[MeSH Terms] OR "epidemiology s"[All Fields]) OR ("mortality"[MeSH Terms] OR "mortality"[All Fields] OR "mortalities"[All Fields] OR "mortality"[MeSH Subheading])) AND ("coronary artery disease"[MeSH Terms] OR ("coronary"[All Fields] AND "artery"[All Fields] AND "disease"[All Fields]) OR "coronary artery disease"[All Fields] OR ("cardiovascular diseases"[MeSH Terms] OR ("cardiovascular"[All Fields] AND "diseases"[All Fields]) OR "cardiovascular diseases"[All Fields]) OR ("acute coronary syndrome"[MeSH Terms] OR ("acute"[All Fields] AND "coronary"[All Fields] AND "syndrome"[All Fields]) OR "acute coronary syndrome"[All Fields]) OR ("chronic disease"[MeSH Terms] OR ("chronic"[All Fields] AND "disease"[All Fields]) OR "chronic disease"[All Fields]) OR ("disease attributes"[MeSH Terms] OR ("disease"[All Fields] AND "attributes"[All Fields]) OR "disease attributes"[All Fields]) OR ("respiratory distress syndrome"[MeSH Terms] OR ("respiratory"[All Fields] AND "distress"[All Fields] AND "syndrome"[All Fields]) OR "respiratory distress syndrome"[All Fields]) OR ("respiratory distress syndrome"[MeSH Terms] OR ("respiratory"[All Fields] AND "distress"[All Fields] AND "syndrome"[All Fields]) OR "respiratory distress syndrome"[All Fields] OR "ards"[All Fields]))) NOT ("animals"[MeSH Terms:noexp] OR "animal"[All Fields])) AND ((clinicalstudy[Filter] OR clinicaltrial[Filter] OR clinicaltrialphasei[Filter] OR clinicaltrialphaseii[Filter] OR clinicaltrialphaseiii[Filter] OR clinicaltrialphaseiv[Filter] OR comparativestudy[Filter] OR controlledclinicaltrial[Filter] OR correctedandrepublishedarticle[Filter] OR dataset[Filter] OR electronicsupplementarymaterials[Filter] OR englishabstract[Filter] OR evaluationstudy[Filter] OR governmentpublication[Filter] OR guideline[Filter] OR introductoryjournalarticle[Filter] OR multicenterstudy[Filter] OR observationalstudy[Filter] OR practiceguideline[Filter] OR randomizedcontrolledtrial[Filter] OR technicalreport[Filter] OR validationstudy[Filter]) AND (humans[Filter]) AND (english[Filter]) AND (2019:2022[pdat])) | 31,516 |
| 40 | ((SARS-CoV-2 OR COVID-19 OR Coronavirus Infections OR 2019-nCoV OR coronavirus disease 2019 OR Hospitalization) AND (Prevalence OR Risk Factors OR Risk Assessment OR Epidemiology OR Mortality)) AND (Coronary Artery Disease OR Cardiovascular Diseases OR Acute Coronary Syndrome OR Chronic Disease OR Disease Attributes OR Respiratory Distress Syndrome OR ARDS) NOT animal | Classical Article, Clinical Study, Clinical Trial, Clinical Trial, Phase I, Clinical Trial, Phase II, Clinical Trial, Phase III, Clinical Trial, Phase IV, Comparative Study, Controlled Clinical Trial, Corrected and Republished Article, Dataset, Electronic Supplementary Materials, English Abstract, Evaluation Study, Government Publication, Guideline, Introductory Journal Article, Multicenter Study, Observational Study, Practice Guideline, Randomized Controlled Trial, Technical Report, Validation Study, Humans, English, from 2019 - 2022 | ((("sars cov 2"[MeSH Terms] OR "sars cov 2"[All Fields] OR "sars cov 2"[All Fields] OR ("covid 19"[All Fields] OR "covid 19"[MeSH Terms] OR "covid 19 vaccines"[All Fields] OR "covid 19 vaccines"[MeSH Terms] OR "covid 19 serotherapy"[All Fields] OR "covid 19 serotherapy"[Supplementary Concept] OR "covid 19 nucleic acid testing"[All Fields] OR "covid 19 nucleic acid testing"[MeSH Terms] OR "covid 19 serological testing"[All Fields] OR "covid 19 serological testing"[MeSH Terms] OR "covid 19 testing"[All Fields] OR "covid 19 testing"[MeSH Terms] OR "sars cov 2"[All Fields] OR "sars cov 2"[MeSH Terms] OR "severe acute respiratory syndrome coronavirus 2"[All Fields] OR "ncov"[All Fields] OR "2019 ncov"[All Fields] OR (("coronavirus"[MeSH Terms] OR "coronavirus"[All Fields] OR "cov"[All Fields]) AND 2019/11/01:3000/12/31[Date - Publication])) OR ("coronavirus infections"[MeSH Terms] OR ("coronavirus"[All Fields] AND "infections"[All Fields]) OR "coronavirus infections"[All Fields]) OR ("sars cov 2"[MeSH Terms] OR "sars cov 2"[All Fields] OR "2019 ncov"[All Fields]) OR ("covid 19"[MeSH Terms] OR "covid 19"[All Fields] OR "coronavirus disease 2019"[All Fields]) OR ("hospital s"[All Fields] OR "hospitalisation"[All Fields] OR "hospitalization"[MeSH Terms] OR "hospitalization"[All Fields] OR "hospitalising"[All Fields] OR "hospitality"[All Fields] OR "hospitalisations"[All Fields] OR "hospitalised"[All Fields] OR "hospitalizations"[All Fields] OR "hospitalized"[All Fields] OR "hospitalize"[All Fields] OR "hospitalizing"[All Fields] OR "hospitals"[MeSH Terms] OR "hospitals"[All Fields] OR "hospital"[All Fields])) AND ("epidemiology"[MeSH Subheading] OR "epidemiology"[All Fields] OR "prevalence"[All Fields] OR "prevalence"[MeSH Terms] OR "prevalance"[All Fields] OR "prevalences"[All Fields] OR "prevalence s"[All Fields] OR "prevalent"[All Fields] OR "prevalently"[All Fields] OR "prevalents"[All Fields] OR ("risk factors"[MeSH Terms] OR ("risk"[All Fields] AND "factors"[All Fields]) OR "risk factors"[All Fields]) OR ("risk assessment"[MeSH Terms] OR ("risk"[All Fields] AND "assessment"[All Fields]) OR "risk assessment"[All Fields]) OR ("epidemiologies"[All Fields] OR "epidemiology"[MeSH Subheading] OR "epidemiology"[All Fields] OR "epidemiology"[MeSH Terms] OR "epidemiology s"[All Fields]) OR ("mortality"[MeSH Terms] OR "mortality"[All Fields] OR "mortalities"[All Fields] OR "mortality"[MeSH Subheading])) AND ("coronary artery disease"[MeSH Terms] OR ("coronary"[All Fields] AND "artery"[All Fields] AND "disease"[All Fields]) OR "coronary artery disease"[All Fields] OR ("cardiovascular diseases"[MeSH Terms] OR ("cardiovascular"[All Fields] AND "diseases"[All Fields]) OR "cardiovascular diseases"[All Fields]) OR ("acute coronary syndrome"[MeSH Terms] OR ("acute"[All Fields] AND "coronary"[All Fields] AND "syndrome"[All Fields]) OR "acute coronary syndrome"[All Fields]) OR ("chronic disease"[MeSH Terms] OR ("chronic"[All Fields] AND "disease"[All Fields]) OR "chronic disease"[All Fields]) OR ("disease attributes"[MeSH Terms] OR ("disease"[All Fields] AND "attributes"[All Fields]) OR "disease attributes"[All Fields]) OR ("respiratory distress syndrome"[MeSH Terms] OR ("respiratory"[All Fields] AND "distress"[All Fields] AND "syndrome"[All Fields]) OR "respiratory distress syndrome"[All Fields]) OR ("respiratory distress syndrome"[MeSH Terms] OR ("respiratory"[All Fields] AND "distress"[All Fields] AND "syndrome"[All Fields]) OR "respiratory distress syndrome"[All Fields] OR "ards"[All Fields]))) NOT ("animals"[MeSH Terms:noexp] OR "animal"[All Fields])) AND ((classicalarticle[Filter] OR clinicalstudy[Filter] OR clinicaltrial[Filter] OR clinicaltrialphasei[Filter] OR clinicaltrialphaseii[Filter] OR clinicaltrialphaseiii[Filter] OR clinicaltrialphaseiv[Filter] OR comparativestudy[Filter] OR controlledclinicaltrial[Filter] OR correctedandrepublishedarticle[Filter] OR dataset[Filter] OR electronicsupplementarymaterials[Filter] OR englishabstract[Filter] OR evaluationstudy[Filter] OR governmentpublication[Filter] OR guideline[Filter] OR introductoryjournalarticle[Filter] OR multicenterstudy[Filter] OR observationalstudy[Filter] OR practiceguideline[Filter] OR randomizedcontrolledtrial[Filter] OR technicalreport[Filter] OR validationstudy[Filter]) AND (humans[Filter]) AND (english[Filter]) AND (2019:2022[pdat])) | 31,517 |
| 41 | ((SARS-CoV-2 OR COVID-19 OR Coronavirus Infections OR 2019-nCoV OR coronavirus disease 2019 OR Hospitalization) AND (Prevalence OR Risk Factors OR Risk Assessment OR Epidemiology OR Mortality)) AND (Coronary Artery Disease OR Cardiovascular Diseases OR Acute Coronary Syndrome OR Chronic Disease OR Disease Attributes OR Respiratory Distress Syndrome OR ARDS) NOT animal Filters: Classical Article, Clinical Study, Clinical Trial, Clinical Trial, Phase I, Clinical Trial, Phase II, Clinical Trial, Phase III, Clinical Trial, Phase IV, Comparative Study, Controlled Clinical Trial, Corrected and Republished Article, Dataset, Electronic Supplementary Materials, English Abstract, Evaluation Study, Government Publication, Guideline, Introductory Journal Article, Multicenter Study, Observational Study, Practice Guideline, Randomized Controlled Trial, Technical Report, Validation Study, Humans, English, AND 2019/11/01:2022/03/20 [Date - Publication] | Classical Article, Clinical Study, Clinical Trial, Clinical Trial, Phase I, Clinical Trial, Phase II, Clinical Trial, Phase III, Clinical Trial, Phase IV, Comparative Study, Controlled Clinical Trial, Corrected and Republished Article, Dataset, Electronic Supplementary Materials, English Abstract, Evaluation Study, Government Publication, Guideline, Introductory Journal Article, Multicenter Study, Observational Study, Practice Guideline, Randomized Controlled Trial, Technical Report, Validation Study, Humans, English | (((("sars cov 2"[MeSH Terms] OR "sars cov 2"[All Fields] OR "sars cov 2"[All Fields] OR ("covid 19"[All Fields] OR "covid 19"[MeSH Terms] OR "covid 19 vaccines"[All Fields] OR "covid 19 vaccines"[MeSH Terms] OR "covid 19 serotherapy"[All Fields] OR "covid 19 serotherapy"[Supplementary Concept] OR "covid 19 nucleic acid testing"[All Fields] OR "covid 19 nucleic acid testing"[MeSH Terms] OR "covid 19 serological testing"[All Fields] OR "covid 19 serological testing"[MeSH Terms] OR "covid 19 testing"[All Fields] OR "covid 19 testing"[MeSH Terms] OR "sars cov 2"[All Fields] OR "sars cov 2"[MeSH Terms] OR "severe acute respiratory syndrome coronavirus 2"[All Fields] OR "ncov"[All Fields] OR "2019 ncov"[All Fields] OR (("coronavirus"[MeSH Terms] OR "coronavirus"[All Fields] OR "cov"[All Fields]) AND 2019/11/01:3000/12/31[Date - Publication])) OR ("coronavirus infections"[MeSH Terms] OR ("coronavirus"[All Fields] AND "infections"[All Fields]) OR "coronavirus infections"[All Fields]) OR ("sars cov 2"[MeSH Terms] OR "sars cov 2"[All Fields] OR "2019 ncov"[All Fields]) OR ("covid 19"[MeSH Terms] OR "covid 19"[All Fields] OR "coronavirus disease 2019"[All Fields]) OR ("hospital s"[All Fields] OR "hospitalisation"[All Fields] OR "hospitalization"[MeSH Terms] OR "hospitalization"[All Fields] OR "hospitalising"[All Fields] OR "hospitality"[All Fields] OR "hospitalisations"[All Fields] OR "hospitalised"[All Fields] OR "hospitalizations"[All Fields] OR "hospitalized"[All Fields] OR "hospitalize"[All Fields] OR "hospitalizing"[All Fields] OR "hospitals"[MeSH Terms] OR "hospitals"[All Fields] OR "hospital"[All Fields])) AND ("epidemiology"[MeSH Subheading] OR "epidemiology"[All Fields] OR "prevalence"[All Fields] OR "prevalence"[MeSH Terms] OR "prevalance"[All Fields] OR "prevalences"[All Fields] OR "prevalence s"[All Fields] OR "prevalent"[All Fields] OR "prevalently"[All Fields] OR "prevalents"[All Fields] OR ("risk factors"[MeSH Terms] OR ("risk"[All Fields] AND "factors"[All Fields]) OR "risk factors"[All Fields]) OR ("risk assessment"[MeSH Terms] OR ("risk"[All Fields] AND "assessment"[All Fields]) OR "risk assessment"[All Fields]) OR ("epidemiologies"[All Fields] OR "epidemiology"[MeSH Subheading] OR "epidemiology"[All Fields] OR "epidemiology"[MeSH Terms] OR "epidemiology s"[All Fields]) OR ("mortality"[MeSH Terms] OR "mortality"[All Fields] OR "mortalities"[All Fields] OR "mortality"[MeSH Subheading])) AND ("coronary artery disease"[MeSH Terms] OR ("coronary"[All Fields] AND "artery"[All Fields] AND "disease"[All Fields]) OR "coronary artery disease"[All Fields] OR ("cardiovascular diseases"[MeSH Terms] OR ("cardiovascular"[All Fields] AND "diseases"[All Fields]) OR "cardiovascular diseases"[All Fields]) OR ("acute coronary syndrome"[MeSH Terms] OR ("acute"[All Fields] AND "coronary"[All Fields] AND "syndrome"[All Fields]) OR "acute coronary syndrome"[All Fields]) OR ("chronic disease"[MeSH Terms] OR ("chronic"[All Fields] AND "disease"[All Fields]) OR "chronic disease"[All Fields]) OR ("disease attributes"[MeSH Terms] OR ("disease"[All Fields] AND "attributes"[All Fields]) OR "disease attributes"[All Fields]) OR ("respiratory distress syndrome"[MeSH Terms] OR ("respiratory"[All Fields] AND "distress"[All Fields] AND "syndrome"[All Fields]) OR "respiratory distress syndrome"[All Fields]) OR ("respiratory distress syndrome"[MeSH Terms] OR ("respiratory"[All Fields] AND "distress"[All Fields] AND "syndrome"[All Fields]) OR "respiratory distress syndrome"[All Fields] OR "ards"[All Fields]))) NOT (("animals"[MeSH Terms:noexp] OR "animal"[All Fields]) AND ("filter"[All Fields] OR "filter s"[All Fields] OR "filtered"[All Fields] OR "filtering"[All Fields] OR "filterings"[All Fields] OR "filters"[All Fields]) AND ("classical article"[Publication Type] OR "classical article"[All Fields]) AND ("clinical study"[Publication Type] OR "clinical studies as topic"[MeSH Terms] OR "clinical study"[All Fields]) AND ("clinical trial"[Publication Type] OR "clinical trials as topic"[MeSH Terms] OR "clinical trial"[All Fields]) AND ("clinical trials, phase i as topic"[MeSH Terms] OR "phase i as topic clinical trials"[All Fields] OR "clinical trial phase i"[All Fields]) AND ("clinical trials, phase ii as topic"[MeSH Terms] OR ("clinical"[All Fields] AND "trials"[All Fields] AND "phase"[All Fields] AND "ii"[All Fields] AND "topic"[All Fields]) OR "phase ii as topic clinical trials"[All Fields] OR ("clinical"[All Fields] AND "trial"[All Fields] AND "phase"[All Fields] AND "ii"[All Fields]) OR "clinical trial phase ii"[All Fields]) AND ("clinical trials, phase iii as topic"[MeSH Terms] OR ("clinical"[All Fields] AND "trials"[All Fields] AND "phase"[All Fields] AND "iii"[All Fields] AND "topic"[All Fields]) OR "phase iii as topic clinical trials"[All Fields] OR ("clinical"[All Fields] AND "trial"[All Fields] AND "phase"[All Fields] AND "iii"[All Fields]) OR "clinical trial phase iii"[All Fields]) AND ("clinical trials, phase iv as topic"[MeSH Terms] OR ("clinical"[All Fields] AND "trials"[All Fields] AND "phase"[All Fields] AND "iv"[All Fields] AND "topic"[All Fields]) OR "phase iv as topic clinical trials"[All Fields] OR ("clinical"[All Fields] AND "trial"[All Fields] AND "phase"[All Fields] AND "iv"[All Fields]) OR "clinical trial phase iv"[All Fields]) AND ("comparative study"[Publication Type] OR "comparative study"[All Fields]) AND ("controlled clinical trial"[Publication Type] OR "controlled clinical trials as topic"[MeSH Terms] OR "controlled clinical trial"[All Fields]) AND ("corrected and republished article"[Publication Type] OR "corrected and republished article"[All Fields]) AND ("dataset"[Publication Type] OR "datasets as topic"[MeSH Terms] OR "dataset"[All Fields]) AND ("electronic supplementary materials"[Publication Type] OR "electronic supplementary materials"[All Fields]) AND ("english abstract"[Publication Type] OR "english abstract"[All Fields]) AND ("evaluation study"[Publication Type] OR "evaluation studies as topic"[MeSH Terms] OR "evaluation study"[All Fields]) AND ("government publication"[Publication Type] OR "government publications as topic"[MeSH Terms] OR "government publication"[All Fields]) AND ("guideline"[Publication Type] OR "guidelines as topic"[MeSH Terms] OR "guideline"[All Fields]) AND ("introductory journal article"[Publication Type] OR "introductory journal article"[All Fields]) AND ("multicenter study"[Publication Type] OR "multicenter studies as topic"[MeSH Terms] OR "multicenter study"[All Fields] OR "multicentre study"[All Fields]) AND ("observational study"[Publication Type] OR "observational studies as topic"[MeSH Terms] OR "observational study"[All Fields]) AND ("practice guideline"[Publication Type] OR "practice guidelines as topic"[MeSH Terms] OR "practice guideline"[All Fields]) AND ("randomized controlled trial"[Publication Type] OR "randomized controlled trials as topic"[MeSH Terms] OR "randomized controlled trial"[All Fields] OR "randomised controlled trial"[All Fields]) AND ("technical report"[Publication Type] OR "technical report"[All Fields]) AND ("validation study"[Publication Type] OR "validation studies as topic"[MeSH Terms] OR "validation study"[All Fields]) AND ("human s"[All Fields] OR "humans"[MeSH Terms] OR "humans"[All Fields] OR "human"[All Fields]) AND "English"[All Fields])) AND 2019/11/01:2022/03/20[Date - Publication]) AND ((classicalarticle[Filter] OR clinicalstudy[Filter] OR clinicaltrial[Filter] OR clinicaltrialphasei[Filter] OR clinicaltrialphaseii[Filter] OR clinicaltrialphaseiii[Filter] OR clinicaltrialphaseiv[Filter] OR comparativestudy[Filter] OR controlledclinicaltrial[Filter] OR correctedandrepublishedarticle[Filter] OR dataset[Filter] OR electronicsupplementarymaterials[Filter] OR englishabstract[Filter] OR evaluationstudy[Filter] OR governmentpublication[Filter] OR guideline[Filter] OR introductoryjournalarticle[Filter] OR multicenterstudy[Filter] OR observationalstudy[Filter] OR practiceguideline[Filter] OR randomizedcontrolledtrial[Filter] OR technicalreport[Filter] OR validationstudy[Filter]) AND (humans[Filter]) AND (english[Filter])) | 23,085 |
| 42 | covid[Title/Abstract] | Classical Article, Clinical Study, Clinical Trial, Clinical Trial, Phase I, Clinical Trial, Phase II, Clinical Trial, Phase III, Clinical Trial, Phase IV, Comparative Study, Controlled Clinical Trial, Corrected and Republished Article, Dataset, Electronic Supplementary Materials, English Abstract, Evaluation Study, Government Publication, Guideline, Introductory Journal Article, Multicenter Study, Observational Study, Practice Guideline, Randomized Controlled Trial, Technical Report, Validation Study, Humans, English | ("covid"[Title/Abstract]) AND ((classicalarticle[Filter] OR clinicalstudy[Filter] OR clinicaltrial[Filter] OR clinicaltrialphasei[Filter] OR clinicaltrialphaseii[Filter] OR clinicaltrialphaseiii[Filter] OR clinicaltrialphaseiv[Filter] OR comparativestudy[Filter] OR controlledclinicaltrial[Filter] OR correctedandrepublishedarticle[Filter] OR dataset[Filter] OR electronicsupplementarymaterials[Filter] OR englishabstract[Filter] OR evaluationstudy[Filter] OR governmentpublication[Filter] OR guideline[Filter] OR introductoryjournalarticle[Filter] OR multicenterstudy[Filter] OR observationalstudy[Filter] OR practiceguideline[Filter] OR randomizedcontrolledtrial[Filter] OR technicalreport[Filter] OR validationstudy[Filter]) AND (humans[Filter]) AND (english[Filter])) | 11,250 |
| 44 | (("SARS-CoV-2" OR "COVID-19" OR "Coronavirus Infections" OR "2019-nCoV" OR "coronavirus disease 2019") AND ("Prevalence" OR "Risk Factors" OR "Risk Assessment" OR "Epidemiology" OR "Mortality")) AND ("Coronary Artery Disease"[TIAB] OR "Cardiovascular Diseases"[TIAB] OR "Acute Coronary Syndrome"[TIAB] OR "Respiratory Distress Syndrome" [TIAB] OR "ARDS" [TIAB]) AND 2019/11/01:2022/03/20 [Date - Publication] NOT (animal[Filter] OR rat OR rodent) | Classical Article, Clinical Study, Clinical Trial, Clinical Trial, Phase I, Clinical Trial, Phase II, Clinical Trial, Phase III, Clinical Trial, Phase IV, Comparative Study, Controlled Clinical Trial, Corrected and Republished Article, Dataset, Electronic Supplementary Materials, English Abstract, Evaluation Study, Government Publication, Guideline, Introductory Journal Article, Multicenter Study, Observational Study, Practice Guideline, Randomized Controlled Trial, Technical Report, Validation Study, Humans, English | ((("SARS-CoV-2"[All Fields] OR "COVID-19"[All Fields] OR "Coronavirus Infections"[All Fields] OR "2019-nCoV"[All Fields] OR "coronavirus disease 2019"[All Fields]) AND ("Prevalence"[All Fields] OR "Risk Factors"[All Fields] OR "Risk Assessment"[All Fields] OR "Epidemiology"[All Fields] OR "Mortality"[All Fields]) AND ("Coronary Artery Disease"[Title/Abstract] OR "Cardiovascular Diseases"[Title/Abstract] OR "Acute Coronary Syndrome"[Title/Abstract] OR "Respiratory Distress Syndrome"[Title/Abstract] OR "ARDS"[Title/Abstract]) AND 2019/11/01:2022/03/20[Date - Publication]) NOT ("animals"[MeSH Terms:noexp] OR ("rats"[MeSH Terms] OR "rats"[All Fields] OR "rat"[All Fields]) OR ("rodent s"[All Fields] OR "rodentia"[MeSH Terms] OR "rodentia"[All Fields] OR "rodent"[All Fields] OR "rodents"[All Fields]))) AND ((classicalarticle[Filter] OR clinicalstudy[Filter] OR clinicaltrial[Filter] OR clinicaltrialphasei[Filter] OR clinicaltrialphaseii[Filter] OR clinicaltrialphaseiii[Filter] OR clinicaltrialphaseiv[Filter] OR comparativestudy[Filter] OR controlledclinicaltrial[Filter] OR correctedandrepublishedarticle[Filter] OR dataset[Filter] OR electronicsupplementarymaterials[Filter] OR englishabstract[Filter] OR evaluationstudy[Filter] OR governmentpublication[Filter] OR guideline[Filter] OR introductoryjournalarticle[Filter] OR multicenterstudy[Filter] OR observationalstudy[Filter] OR practiceguideline[Filter] OR randomizedcontrolledtrial[Filter] OR technicalreport[Filter] OR validationstudy[Filter]) AND (humans[Filter]) AND (english[Filter])) | 565 |
| 45 | (("SARS-CoV-2" OR "COVID-19" OR "Coronavirus Infections" OR "2019-nCoV" OR "coronavirus disease 2019") AND ("Prevalence" OR "Risk Factors" OR "Risk Assessment" OR "Epidemiology" OR "Mortality")) AND ("Coronary Artery Disease"[TIAB] OR "Cardiovascular Diseases"[TIAB] OR "Acute Coronary Syndrome"[TIAB] OR "Respiratory Distress Syndrome" [TIAB]) AND 2019/11/01:2022/03/20 [Date - Publication] NOT (animal[Filter] OR rat OR rodent) | Classical Article, Clinical Study, Clinical Trial, Clinical Trial, Phase I, Clinical Trial, Phase II, Clinical Trial, Phase III, Clinical Trial, Phase IV, Comparative Study, Controlled Clinical Trial, Corrected and Republished Article, Dataset, Electronic Supplementary Materials, English Abstract, Evaluation Study, Government Publication, Guideline, Introductory Journal Article, Multicenter Study, Observational Study, Practice Guideline, Randomized Controlled Trial, Technical Report, Validation Study, Humans, English | ((("SARS-CoV-2"[All Fields] OR "COVID-19"[All Fields] OR "Coronavirus Infections"[All Fields] OR "2019-nCoV"[All Fields] OR "coronavirus disease 2019"[All Fields]) AND ("Prevalence"[All Fields] OR "Risk Factors"[All Fields] OR "Risk Assessment"[All Fields] OR "Epidemiology"[All Fields] OR "Mortality"[All Fields]) AND ("Coronary Artery Disease"[Title/Abstract] OR "Cardiovascular Diseases"[Title/Abstract] OR "Acute Coronary Syndrome"[Title/Abstract] OR "Respiratory Distress Syndrome"[Title/Abstract]) AND 2019/11/01:2022/03/20[Date - Publication]) NOT ("animals"[MeSH Terms:noexp] OR ("rats"[MeSH Terms] OR "rats"[All Fields] OR "rat"[All Fields]) OR ("rodent s"[All Fields] OR "rodentia"[MeSH Terms] OR "rodentia"[All Fields] OR "rodent"[All Fields] OR "rodents"[All Fields]))) AND ((classicalarticle[Filter] OR clinicalstudy[Filter] OR clinicaltrial[Filter] OR clinicaltrialphasei[Filter] OR clinicaltrialphaseii[Filter] OR clinicaltrialphaseiii[Filter] OR clinicaltrialphaseiv[Filter] OR comparativestudy[Filter] OR controlledclinicaltrial[Filter] OR correctedandrepublishedarticle[Filter] OR dataset[Filter] OR electronicsupplementarymaterials[Filter] OR englishabstract[Filter] OR evaluationstudy[Filter] OR governmentpublication[Filter] OR guideline[Filter] OR introductoryjournalarticle[Filter] OR multicenterstudy[Filter] OR observationalstudy[Filter] OR practiceguideline[Filter] OR randomizedcontrolledtrial[Filter] OR technicalreport[Filter] OR validationstudy[Filter]) AND (humans[Filter]) AND (english[Filter])) | 492 |
| 47 | (("SARS-CoV-2" OR "COVID-19" OR "Coronavirus Infections" OR "2019-nCoV" OR "coronavirus disease 2019" AND ((review[Filter] OR systematicreview[Filter]) AND (humans[Filter]) AND (english[Filter]) AND (2019:2022[pdat]))) AND (("Prevalence" OR "Risk Factors" OR "Risk Assessment" OR "Epidemiology" OR "Mortality") AND ((review[Filter] OR systematicreview[Filter]) AND (humans[Filter]) AND (english[Filter])))) AND (("Coronary Artery Disease"[TIAB] OR "Cardiovascular Diseases"[TIAB] OR "Acute Coronary Syndrome"[TIAB] AND ((review[Filter] OR systematicreview[Filter]) AND (humans[Filter]) AND (english[Filter])))) | Classical Article, Clinical Study, Clinical Trial, Clinical Trial, Phase I, Clinical Trial, Phase II, Clinical Trial, Phase III, Clinical Trial, Phase IV, Comparative Study, Controlled Clinical Trial, Corrected and Republished Article, Dataset, Electronic Supplementary Materials, English Abstract, Evaluation Study, Government Publication, Guideline, Introductory Journal Article, Multicenter Study, Observational Study, Practice Guideline, Randomized Controlled Trial, Technical Report, Validation Study, Humans, English | (("SARS-CoV-2"[All Fields] OR "COVID-19"[All Fields] OR "Coronavirus Infections"[All Fields] OR "2019-nCoV"[All Fields] OR "coronavirus disease 2019"[All Fields]) AND (("review"[Publication Type] OR "systematic review"[Filter]) AND "humans"[MeSH Terms] AND "english"[Language] AND 2019/01/01:2022/12/31[Date - Publication]) AND (("Prevalence"[All Fields] OR "Risk Factors"[All Fields] OR "Risk Assessment"[All Fields] OR "Epidemiology"[All Fields] OR "Mortality"[All Fields]) AND (("review"[Publication Type] OR "systematic review"[Filter]) AND "humans"[MeSH Terms] AND "english"[Language])) AND (("Coronary Artery Disease"[Title/Abstract] OR "Cardiovascular Diseases"[Title/Abstract] OR "Acute Coronary Syndrome"[Title/Abstract]) AND (("review"[Publication Type] OR "systematic review"[Filter]) AND "humans"[MeSH Terms] AND "english"[Language]))) AND ((classicalarticle[Filter] OR clinicalstudy[Filter] OR clinicaltrial[Filter] OR clinicaltrialphasei[Filter] OR clinicaltrialphaseii[Filter] OR clinicaltrialphaseiii[Filter] OR clinicaltrialphaseiv[Filter] OR comparativestudy[Filter] OR controlledclinicaltrial[Filter] OR correctedandrepublishedarticle[Filter] OR dataset[Filter] OR electronicsupplementarymaterials[Filter] OR englishabstract[Filter] OR evaluationstudy[Filter] OR governmentpublication[Filter] OR guideline[Filter] OR introductoryjournalarticle[Filter] OR multicenterstudy[Filter] OR observationalstudy[Filter] OR practiceguideline[Filter] OR randomizedcontrolledtrial[Filter] OR technicalreport[Filter] OR validationstudy[Filter]) AND (humans[Filter]) AND (english[Filter])) | 3 |
| 48 | (("SARS-CoV-2" OR "COVID-19" OR "Coronavirus Infections" OR "2019-nCoV" OR "coronavirus disease 2019" AND ((review[Filter] OR systematicreview[Filter]) AND (humans[Filter]) AND (english[Filter]) AND (2019:2022[pdat]))) AND (("Prevalence" OR "Risk Factors" OR "Risk Assessment" OR "Epidemiology" OR "Mortality") AND ((review[Filter] OR systematicreview[Filter]) AND (humans[Filter]) AND (english[Filter])))) AND (("Coronary Artery Disease"[TIAB] OR "Cardiovascular Diseases"[TIAB] OR "Acute Coronary Syndrome"[TIAB] AND ((review[Filter] OR systematicreview[Filter]) AND (humans[Filter]) AND (english[Filter])))) | Classical Article, Clinical Study, Clinical Trial, Clinical Trial, Phase I, Clinical Trial, Phase II, Clinical Trial, Phase III, Clinical Trial, Phase IV, Comparative Study, Controlled Clinical Trial, Corrected and Republished Article, Dataset, Electronic Supplementary Materials, English Abstract, Evaluation Study, Government Publication, Guideline, Introductory Journal Article, Multicenter Study, Observational Study, Practice Guideline, Randomized Controlled Trial, Technical Report, Humans, English | (("SARS-CoV-2"[All Fields] OR "COVID-19"[All Fields] OR "Coronavirus Infections"[All Fields] OR "2019-nCoV"[All Fields] OR "coronavirus disease 2019"[All Fields]) AND (("review"[Publication Type] OR "systematic review"[Filter]) AND "humans"[MeSH Terms] AND "english"[Language] AND 2019/01/01:2022/12/31[Date - Publication]) AND (("Prevalence"[All Fields] OR "Risk Factors"[All Fields] OR "Risk Assessment"[All Fields] OR "Epidemiology"[All Fields] OR "Mortality"[All Fields]) AND (("review"[Publication Type] OR "systematic review"[Filter]) AND "humans"[MeSH Terms] AND "english"[Language])) AND (("Coronary Artery Disease"[Title/Abstract] OR "Cardiovascular Diseases"[Title/Abstract] OR "Acute Coronary Syndrome"[Title/Abstract]) AND (("review"[Publication Type] OR "systematic review"[Filter]) AND "humans"[MeSH Terms] AND "english"[Language]))) AND ((classicalarticle[Filter] OR clinicalstudy[Filter] OR clinicaltrial[Filter] OR clinicaltrialphasei[Filter] OR clinicaltrialphaseii[Filter] OR clinicaltrialphaseiii[Filter] OR clinicaltrialphaseiv[Filter] OR comparativestudy[Filter] OR controlledclinicaltrial[Filter] OR correctedandrepublishedarticle[Filter] OR dataset[Filter] OR electronicsupplementarymaterials[Filter] OR englishabstract[Filter] OR evaluationstudy[Filter] OR governmentpublication[Filter] OR guideline[Filter] OR introductoryjournalarticle[Filter] OR multicenterstudy[Filter] OR observationalstudy[Filter] OR practiceguideline[Filter] OR randomizedcontrolledtrial[Filter] OR technicalreport[Filter]) AND (humans[Filter]) AND (english[Filter])) | 3 |
| 49 | (("SARS-CoV-2" OR "COVID-19" OR "Coronavirus Infections" OR "2019-nCoV" OR "coronavirus disease 2019" AND ((review[Filter] OR systematicreview[Filter]) AND (humans[Filter]) AND (english[Filter]) AND (2019:2022[pdat]))) AND (("Prevalence" OR "Risk Factors" OR "Risk Assessment" OR "Epidemiology" OR "Mortality") AND ((review[Filter] OR systematicreview[Filter]) AND (humans[Filter]) AND (english[Filter])))) AND (("Coronary Artery Disease"[TIAB] OR "Cardiovascular Diseases"[TIAB] OR "Acute Coronary Syndrome"[TIAB] AND ((review[Filter] OR systematicreview[Filter]) AND (humans[Filter]) AND (english[Filter])))) | Clinical Study, Clinical Trial, Clinical Trial, Phase I, Clinical Trial, Phase II, Clinical Trial, Phase III, Clinical Trial, Phase IV, Comparative Study, Controlled Clinical Trial, Corrected and Republished Article, Dataset, Electronic Supplementary Materials, English Abstract, Evaluation Study, Government Publication, Guideline, Introductory Journal Article, Multicenter Study, Observational Study, Practice Guideline, Randomized Controlled Trial, Technical Report, Humans, English | (("SARS-CoV-2"[All Fields] OR "COVID-19"[All Fields] OR "Coronavirus Infections"[All Fields] OR "2019-nCoV"[All Fields] OR "coronavirus disease 2019"[All Fields]) AND (("review"[Publication Type] OR "systematic review"[Filter]) AND "humans"[MeSH Terms] AND "english"[Language] AND 2019/01/01:2022/12/31[Date - Publication]) AND (("Prevalence"[All Fields] OR "Risk Factors"[All Fields] OR "Risk Assessment"[All Fields] OR "Epidemiology"[All Fields] OR "Mortality"[All Fields]) AND (("review"[Publication Type] OR "systematic review"[Filter]) AND "humans"[MeSH Terms] AND "english"[Language])) AND (("Coronary Artery Disease"[Title/Abstract] OR "Cardiovascular Diseases"[Title/Abstract] OR "Acute Coronary Syndrome"[Title/Abstract]) AND (("review"[Publication Type] OR "systematic review"[Filter]) AND "humans"[MeSH Terms] AND "english"[Language]))) AND ((clinicalstudy[Filter] OR clinicaltrial[Filter] OR clinicaltrialphasei[Filter] OR clinicaltrialphaseii[Filter] OR clinicaltrialphaseiii[Filter] OR clinicaltrialphaseiv[Filter] OR comparativestudy[Filter] OR controlledclinicaltrial[Filter] OR correctedandrepublishedarticle[Filter] OR dataset[Filter] OR electronicsupplementarymaterials[Filter] OR englishabstract[Filter] OR evaluationstudy[Filter] OR governmentpublication[Filter] OR guideline[Filter] OR introductoryjournalarticle[Filter] OR multicenterstudy[Filter] OR observationalstudy[Filter] OR practiceguideline[Filter] OR randomizedcontrolledtrial[Filter] OR technicalreport[Filter]) AND (humans[Filter]) AND (english[Filter])) | 3 |
| 50 | (("SARS-CoV-2" OR "COVID-19" OR "Coronavirus Infections" OR "2019-nCoV" OR "coronavirus disease 2019" AND ((review[Filter] OR systematicreview[Filter]) AND (humans[Filter]) AND (english[Filter]) AND (2019:2022[pdat]))) AND (("Prevalence" OR "Risk Factors" OR "Risk Assessment" OR "Epidemiology" OR "Mortality") AND ((review[Filter] OR systematicreview[Filter]) AND (humans[Filter]) AND (english[Filter])))) AND (("Coronary Artery Disease"[TIAB] OR "Cardiovascular Diseases"[TIAB] OR "Acute Coronary Syndrome"[TIAB] AND ((review[Filter] OR systematicreview[Filter]) AND (humans[Filter]) AND (english[Filter])))) |  | ("SARS-CoV-2"[All Fields] OR "COVID-19"[All Fields] OR "Coronavirus Infections"[All Fields] OR "2019-nCoV"[All Fields] OR "coronavirus disease 2019"[All Fields]) AND (("review"[Publication Type] OR "systematic review"[Filter]) AND "humans"[MeSH Terms] AND "english"[Language] AND 2019/01/01:2022/12/31[Date - Publication]) AND (("Prevalence"[All Fields] OR "Risk Factors"[All Fields] OR "Risk Assessment"[All Fields] OR "Epidemiology"[All Fields] OR "Mortality"[All Fields]) AND (("review"[Publication Type] OR "systematic review"[Filter]) AND "humans"[MeSH Terms] AND "english"[Language])) AND (("Coronary Artery Disease"[Title/Abstract] OR "Cardiovascular Diseases"[Title/Abstract] OR "Acute Coronary Syndrome"[Title/Abstract]) AND (("review"[Publication Type] OR "systematic review"[Filter]) AND "humans"[MeSH Terms] AND "english"[Language])) | 220 |
| 52 | (("SARS-CoV-2" OR "COVID-19" OR "Coronavirus Infections" OR "2019-nCoV" OR "coronavirus disease 2019" AND (humans[Filter]) AND (english[Filter]) AND (2019:2022[pdat]))) AND (("Prevalence" OR "Risk Factors" OR "Risk Assessment" OR "Epidemiology" OR "Mortality") AND (humans[Filter]) AND (english[Filter])) AND (("Coronary Artery Disease"[TIAB] OR "Cardiovascular Diseases"[TIAB] OR "Acute Coronary Syndrome"[TIAB] AND (humans[Filter]) AND (english[Filter]))) |  | ("SARS-CoV-2"[All Fields] OR "COVID-19"[All Fields] OR "Coronavirus Infections"[All Fields] OR "2019-nCoV"[All Fields] OR "coronavirus disease 2019"[All Fields]) AND "humans"[MeSH Terms] AND "english"[Language] AND 2019/01/01:2022/12/31[Date - Publication] AND (("Prevalence"[All Fields] OR "Risk Factors"[All Fields] OR "Risk Assessment"[All Fields] OR "Epidemiology"[All Fields] OR "Mortality"[All Fields]) AND "humans"[MeSH Terms] AND "english"[Language]) AND (("Coronary Artery Disease"[Title/Abstract] OR "Cardiovascular Diseases"[Title/Abstract] OR "Acute Coronary Syndrome"[Title/Abstract]) AND "humans"[MeSH Terms] AND "english"[Language]) | 771 |
| 53 | (("SARS-CoV-2" OR "COVID-19" OR "Coronavirus Infections" OR "2019-nCoV" OR "coronavirus disease 2019" AND (humans[Filter]) AND (english[Filter]) AND (2019:2022[pdat]))) |  | ("SARS-CoV-2"[All Fields] OR "COVID-19"[All Fields] OR "Coronavirus Infections"[All Fields] OR "2019-nCoV"[All Fields] OR "coronavirus disease 2019"[All Fields]) AND "humans"[MeSH Terms] AND "english"[Language] AND 2019/01/01:2022/12/31[Date - Publication] | 1,45,288 |
| 54 | (("Prevalence" OR "Risk Factors" OR "Risk Assessment" OR "Epidemiology" OR "Mortality") AND (humans[Filter]) AND (english[Filter])) |  | ("Prevalence"[All Fields] OR "Risk Factors"[All Fields] OR "Risk Assessment"[All Fields] OR "Epidemiology"[All Fields] OR "Mortality"[All Fields]) AND "humans"[MeSH Terms] AND "english"[Language] | 32,33,148 |
| 55 | (("Coronary Artery Disease"[TIAB] OR "Cardiovascular Diseases"[TIAB] OR "Acute Coronary Syndrome"[TIAB] AND (humans[Filter]) AND (english[Filter]))) |  | ("Coronary Artery Disease"[Title/Abstract] OR "Cardiovascular Diseases"[Title/Abstract] OR "Acute Coronary Syndrome"[Title/Abstract]) AND "humans"[MeSH Terms] AND "english"[Language] | 1,32,239 |
| 56 | (((("SARS-CoV-2" OR "COVID-19" OR "Coronavirus Infections" OR "2019-nCoV" OR "coronavirus disease 2019" AND (humans[Filter]) AND (english[Filter]) AND (2019:2022[pdat])))) AND ((("Prevalence" OR "Risk Factors" OR "Risk Assessment" OR "Epidemiology" OR "Mortality") AND (humans[Filter]) AND (english[Filter])))) AND ((("Coronary Artery Disease"[TIAB] OR "Cardiovascular Diseases"[TIAB] OR "Acute Coronary Syndrome"[TIAB] AND (humans[Filter]) AND (english[Filter])))) |  | ("SARS-CoV-2"[All Fields] OR "COVID-19"[All Fields] OR "Coronavirus Infections"[All Fields] OR "2019-nCoV"[All Fields] OR "coronavirus disease 2019"[All Fields]) AND "humans"[MeSH Terms] AND "english"[Language] AND 2019/01/01:2022/12/31[Date - Publication] AND (("Prevalence"[All Fields] OR "Risk Factors"[All Fields] OR "Risk Assessment"[All Fields] OR "Epidemiology"[All Fields] OR "Mortality"[All Fields]) AND "humans"[MeSH Terms] AND "english"[Language]) AND (("Coronary Artery Disease"[Title/Abstract] OR "Cardiovascular Diseases"[Title/Abstract] OR "Acute Coronary Syndrome"[Title/Abstract]) AND "humans"[MeSH Terms] AND "english"[Language]) | 771 |

## 2. Web of Science

Date of search = 21-03-2022

Final search result = 663

### Table S 3 Web of Science search results

| **Search number** | **Search query** | **Search Results** |
| --- | --- | --- |
| 1 | ALL=(("Coronary Artery Disease" OR "Cardiovascular Diseases" OR "Acute Coronary Syndrome")) | 240,460 |
| 2 | ALL=(Prevalence OR Risk Factors OR Risk Assessment OR Epidemiology OR Mortality ) | 3,674,415 |
| 3 | ALL=(SARS-CoV-2 OR COVID-19 OR Coronavirus Infections OR 2019-nCoV OR coronavirus disease 2019) | 272,658 |
| 4 | #1 AND #2 AND #3 and 2016 (Exclude – Publication Years) and Review Articles (Exclude – Document Types) and English (Languages) | 663 |

## 3. World Health Organization Covid-19 Global literature on coronavirus disease

Date of search = 20-03-2022

Final search result = 428

### Table S 4 WHO Covid-19 global literature on coronavirus disease search results

| **Search number** | **Search query** | **Search Results** |
| --- | --- | --- |
| 1 | (SARS-CoV-2) OR (COVID-19) OR (Coronavirus Infections) OR (2019 nCoV) OR (coronavirus disease 2019) | 524,393 |
| 2 | (Prevalence) OR (Risk Factors) OR (Risk Assessment) OR (Epidemiology) OR (Mortality) | 143,336 |
| 3 | (Coronary Artery Disease) OR (Cardiovascular Diseases) OR (Acute Coronary Syndrome) | 7,359 |
| 4 | #1 AND #2 AND #3 | 428 |

## 4. Cochrane Library- Trials

Date of search = 29-04-2022

Final search result = 145

Cochrane Library = 209 (Cochrane Reviews = 44; Cochrane Protocols = 14; **Trials = 145**; Special Collections = 4; Clinical Answers = 2)

### Table S 5 Cochrane Library search results

| **Search number** | **Search query** | **Search Results** |
| --- | --- | --- |
| 1 | SARS-CoV-2 | 442 |
| 2 | COVID-19 | 10643 |
| 3 | Coronavirus Infections | 1333 |
| 4 | 2019 nCoV | 240 |
| 5 | coronavirus disease 2019 | 4046 |
| 6 | {OR #1-#5} | 11065 |
| 7 | Prevalence | 44595 |
| 8 | Risk Factors | 85880 |
| 9 | Risk Assessment | 77815 |
| 10 | Epidemiology | 69535 |
| 11 | Mortality | 106953 |
| 12 | {OR #7-#11} | 278468 |
| 13 | Coronary Artery Disease | 25354 |
| 14 | Cardiovascular Diseases | 24491 |
| 15 | Acute Coronary Syndrome | 7633 |
| 16 | {OR #13-#15} | 52672 |
| 17 | {AND #6, #12, #16} with Cochrane Library publication date from Dec 2019 to present  (("SARS-CoV-2" OR "COVID-19" OR "Coronavirus Infections" OR "2019 nCoV" OR "coronavirus disease 2019") AND ("Prevalence" OR "Risk Factors" OR "Risk Assessment" OR "Epidemiology" OR "Mortality") AND ("Coronary Artery Disease" OR "Cardiovascular Diseases" OR "Acute Coronary Syndrome")) | 209 |

# B. Articles excluded from the review

### Table S 6 Reason for exclusion (after full-text screening) with numbers and percentages

| **Reason for exclusion** | **Numbers** | **Percentage (%)** |
| --- | --- | --- |
| Article does not define CVD (different population) | 18 | 17.48 |
| Different objective | 41 | 39.81 |
| Different outcome | 27 | 26.21 |
| Full text unavailable (including poster abstract) | 16 | 15.53 |
| Review | 1 | 0.97 |
| **Grand Total** | **103** | **100** |

### Table S 7 Details of excluded articles from the review after full-text screening

| **S No.** | **Author** | **Year** | **Title** | **Reason category** |
| --- | --- | --- | --- | --- |
| 1 | Castagna F et. al | 2021 | A History of Heart Failure Is an Independent Risk Factor for Death in Patients Admitted with Coronavirus 19 Disease | Different outcome |
| 2 | Rehman S et. al | 2021 | Association of Mortality-Related Risk Factors in Patients with COVID-19: A Retrospective Cohort Study | Article does not define CVD |
| 3 | Zali A et. al | 2020 | Baseline Characteristics and Associated Factors of Mortality in COVID-19 Patients; An Analysis of 16000 Cases in Tehran, Iran | Article does not define CVD |
| 4 | Phelps M et. al | 2021 | Cardiovascular comorbidities as predictors for severe COVID-19 infection or death | Inclusion criteria excludes CAD |
| 5 | Abrams MP et. al | 2020 | Clinical and cardiac characteristics of COVID-19 mortalities in a diverse New York City Cohort | Study not about CAD |
| 6 | Thomson, R. J. et. al | 2020 | Clinical characteristics and outcomes of critically ill patients with COVID-19 admitted to an intensive care unit in London: A prospective observational cohort study | Inclusion criteria excludes CAD |
| 7 | Xu H et. al | 2020 | Clinical characteristics and risk factors of cardiac involvement in COVID-19 | Inclusion criteria excludes CAD |
| 8 | Silverio A et. al | 2021 | Clinical conditions and echocardiographic parameters associated with mortality in COVID-19 | Different objective |
| 9 | Tavolinejad H et. al | 2021 | Clinical implications and indicators of mortality among patients hospitalized with concurrent COVID-19 and myocardial infarction | Different objective |
| 10 | Rivera-Caravaca JM et. al | 2021 | Clinical profile and prognosis in patients on oral anticoagulation before admission for COVID-19 | Different objective |
| 11 | Jakhmola S et. al | 2020 | Comorbidity Assessment is Essential during COVID-19 Treatment | Inclusion criteria excludes CAD |
| 12 | Kuzan, T. Y. et. al | 2021 | A comparison of clinical, laboratory and chest CT findings of laboratory-confirmed and clinically diagnosed COVID-19 patients at first admission | Inclusion criteria excludes CAD |
| 13 | Watanabe Y et. al | 2021 | Impact of the COVID-19 Pandemic on ST-elevation myocardial infarction from a single-center experience in Tokyo | Different outcome |
| 14 | Del Pinto R et. al | 2020 | Increased cardiovascular death rates in COVID-19 low prevalence area | Different outcome |
| 15 | Sousa GJB et. al | 2020 | Mortality and survival of COVID-19 | Different outcome |
| 16 | Montagnon R et. al | 2020 | Impact of the COVID-19 Pandemic on Emergency Department Use: Focus on Patients Requiring Urgent Revascularization. | Different objective |
| 17 | Cantador E et. al | 2020 | Incidence and consequences of systemic arterial thrombotic events in COVID-19 patients. | Different outcome |
| 18 | Gao X et. al | 2021 | A Bayesian framework for estimating the risk ratio of hospitalization for people with comorbidity infected by SARS-CoV-2 virus. | Different outcome |
| 19 | Wang Y et. al | 2021 | The peak levels of highly sensitive troponin I predicts in-hospital mortality in COVID-19 patients with cardiac injury: a retrospective study. | Different outcome |
| 20 | Asirvatham ES et. al | 2020 | Demystifying the varying case fatality rates (CFR) of COVID-19 in India: Lessons learned and future directions. | Different outcome |
| 21 | Gnavi R et. al | 2020 | Therapy With Agents Acting on the Renin-Angiotensin System and Risk of Severe Acute Respiratory Syndrome Coronavirus 2 Infection. | Different outcome |
| 22 | Sheikh S et. al | 2022 | Cases of acute coronary syndrome and presumed cardiac death prior to arrival at an urban tertiary care hospital in Pakistan during the COVID-19 pandemic. | Different outcome |
| 23 | McKay B et. al | 2021 | Predicting 30 - Day outcomes in emergency department patients discharged with COVID-19. | Different outcome |
| 24 | Holy EW et. al | 2020 | Impact of a nationwide COVID-19 lockdown on acute coronary syndrome referrals. | Different objective |
| 25 | Normando PG et. al | 2021 | Reduction in Hospitalization and Increase in Mortality Due to Cardiovascular Diseases during the COVID-19 Pandemic in Brazil. | Different objective |
| 26 | Sokolski M et. al | 2021 | Impact of Coronavirus Disease 2019 (COVID-19) Outbreak on Acute Admissions at the Emergency and Cardiology Departments Across Europe. | Different objective |
| 27 | Zimmermann GS et. al | 2020 | Coronary calcium scoring assessed on native screening chest CT imaging as predictor for outcome in COVID-19: An analysis of a hospitalized German cohort. | Different objective |
| 28 | Wu J et. al | 2021 | Place and causes of acute cardiovascular mortality during the COVID-19 pandemic. | Different objective |
| 29 | Rashid M et. al | 2021 | Racial differences in management and outcomes of acute myocardial infarction during COVID-19 pandemic. | Different objective |
| 30 | Brant LCC et. al | 2020 | Excess of cardiovascular deaths during the COVID-19 pandemic in Brazilian capital cities. | Different objective |
| 31 | Tarlovskaya EI et. al | 2021 | Analysis of influence of background therapy for comorbidities in the period before infection on the risk of the lethal COVID outcome. Data from the international ACTIV SARS-CoV-2 registry (Â«Analysis of chronic non-infectious diseases dynamics after COVID-19 infection in adult patients SARS-CoV-2Â»). | Full text unavailable |
| 32 | de Havenon A et. al | 2021 | Characteristics and Outcomes Among US Patients Hospitalized for Ischemic Stroke Before vs During the COVID-19 Pandemic. | Different objective |
| 33 | Lukiyanov, M. et. al | 2021 | Prehospital period in patients with COVID-19 and community acquired pneumonia: Age characteristics, cardiovascular comorbidity and pharmacotherapy during epidemic wave (the data of hospital registry) | Full text unavailable |
| 34 | Ye, Y. et. al | 2021 | Characteristics and outcomes in a realworld cohort of rheumatoid arthritis patients with covid-19 | Poster abstract |
| 35 | Karahan, S. et. al | 2020 | The relationship between myocardial injury and laboratory parameters and comorbid diseases in hospitalized COVID-19 patients | Full text unavailable |
| 36 | Icten, Z. et. al | 2020 | PIN178 Impact of Selected Comorbidities on Healthcare Resource Utilization Among Hospitalized Patients with COVID-19 in a US Population | Poster abstract |
| 37 | Gutierrez-Abejon, E et. al | 2021 | A Population-Based Registry Analysis on Hospitalized COVID-19 Patients with Previous Cardiovascular Disease: Clinical Profile, Treatment, and Predictors of Death | Different outcome |
| 38 | Gavrilov, D. et. al | 2021 | Application of a clinical decision support system to assess the severity of the new coronavirus infection COVID-19 | Poster abstract |
| 39 | Allo, I. J. T. et. al | 2021 | Association between hypertension and inhospital mortality in COVID-19 patients | Poster abstract |
| 40 | Weizman, O. et. al | 2021 | Cardiovascular Comorbidities and Covid-19 in Women | Full text unavailable |
| 41 | Russo V et. al | 2020 | Cardiovascular Comorbidities and Pharmacological Treatments of COVID-19 Patients Not Requiring Hospitalization. | Different outcome |
| 42 | Kunal S et. al | 2020 | Cardiovascular complications and its impact on outcomes in COVID-19. | Different outcome |
| 43 | Gujski M et. al | 2021 | Characteristics and Clinical Outcomes of 116,539 Patients Hospitalized with COVID-19-Poland, March-December 2020. | Different outcome |
| 44 | Bhandari S et. al | 2020 | Characteristics, Treatment Outcomes and Role of Hydroxychloroquine among 522 COVID-19 hospitalized patients in Jaipur City: An Epidemio-Clinical Study. | Letter |
| 45 | Mahavar S et. al | 2021 | Clinical and epidemiological profile of Indian COVID-19 patients from Jaipur: a descriptive study. | Different outcome |
| 46 | Matsushita K et. al | 2021 | Clinical features of patients with acute coronary syndrome during the COVID-19 pandemic. | Different objective |
| 47 | Michalski, B et. al | 2021 | Clinical profile and in hospital mortality of invasively managed patients with suspicion of acute coronary syndrome during the COVID-19 pandemic | Poster abstract |
| 48 | Bhandari S et. al | 2020 | Clinical Profile of Covid-19 Infected Patients Admitted in a Tertiary Care Hospital in North India. | Different outcome |
| 49 | Hafiz M et. al | 2020 | Clinical, Radiological Features and Outcome of COVID-19 patients in a Secondary Hospital in Jakarta, Indonesia. | Different outcome |
| 50 | Thiabaud A et. al | 2021 | Cohort profile: SARS-CoV-2/COVID-19 hospitalised patients in Switzerland. | Different outcome |
| 51 | Halvatsiotis P et. al | 2020 | Demographic and clinical features of critically ill patients with COVID-19 in Greece: The burden of diabetes and obesity. | Different outcome |
| 52 | Cabezón Villalba G et. al | 2021 | Impact of the presence of heart disease, cardiovascular medications and cardiac events on outcome in COVID-19. | Different outcome |
| 53 | Sharif N et. al | 2021 | Prevalence and impact of comorbidities on disease prognosis among patients with COVID-19 in Bangladesh: A nationwide study amid the second wave. | Different outcome |
| 54 | Luchian, ML et. al | 2021 | Prognostic Value of Coronary Artery Calcium Score in Hospitalized COVID-19 Patients | Inclusion criteria excludes CAD |
| 55 | Yaroslavskaya, E. et. al | 2021 | Psychological profile of COVID-19 pneumonia survivors three month after hospitalization | Poster abstract |
| 56 | Schiavone, M et. al | 2020 | Redefining the Prognostic Value of High-Sensitivity Troponin in COVID-19 Patients: The Importance of Concomitant Coronary Artery Disease | Different outcome |
| 57 | de Cortina Camarero C et. al | 2021 | SARS-CoV-2 infection: A predisposing factor for acute coronary syndrome. | Different objective |
| 58 | Gujski M et. al | 2022 | The Prevalence of Acute Respiratory Distress Syndrome (ARDS) and Outcomes in Hospitalized Patients with COVID-19-A Study Based on Data from the Polish National Hospital Register. | Different outcome |
| 59 | Siagian, APB et. al | 2021 | The Prevalnce of Cardiovascular Comorbidity in the Severe COVID-19 Patients and Its Correlation to Malignant Ventricular Arrhythmia, Neutrophile-Lymphocyte Ratio (NLR) and D-Dimer | Poster abstract |
| 60 | Nystad W et. al | 2020 | Underlying conditions in adults with COVID-19. | Different outcome |
| 61 | Papafaklis MI et. al | 2020 | "Missing" acute coronary syndrome hospitalizations during the COVID-19 era in Greece: Medical care avoidance combined with a true reduction in incidence? | Different objective |
| 62 | Showkathali R et. al | 2020 | Acute Coronary Syndrome admissions and outcome during COVID-19 Pandemic-Report from large tertiary centre in India. | Different objective |
| 63 | Sutherland N et. al | 2022 | Acute Coronary Syndrome in the COVID-19 Pandemic: Reduced Cases and Increased Ischaemic Time. | Different objective |
| 64 | Yalamanchi, R et. al | 2020 | Cardiac Intensive Care Unit Admissions During COVID-19 Pandemic-Single Center Experience | Different objective |
| 65 | Cecelja, M et. al | 2021 | Cardiovascular health and risk of hospitalization with COVID-19: A Mendelian Randomization study | Different objective |
| 66 | Ying Su et. al | 2020 | Clinical characteristics of Covid-19 patients with re-positive test results: an observational study | Different objective |
| 67 | Matsushita K et. al | 2021 | Clinical features of patients with acute coronary syndrome during the COVID-19 pandemic. | Different objective |
| 68 | Sherpa, K et. al | 2021 | Clinical Profile and Outcome of COVID 19 Patients at Tertiary Cardiovascular Center of Nepal | Different outcome |
| 69 | L'Angiocola, PD et. al | 2021 | COVID-19 and its impact on acute coronary syndrome in-hospital epidemiology: a multifactorial analysis from a single-center Hospital in the north-east region of Italy | Different objective |
| 70 | Mafham MM et. al | 2020 | COVID-19 pandemic and admission rates for and management of acute coronary syndromes in England. | Different objective |
| 71 | Schwarz V et. al | 2020 | Decline of emergency admissions for cardiovascular and cerebrovascular events after the outbreak of COVID-19. | Different objective |
| 72 | Braiteh N et. al | 2020 | Decrease in acute coronary syndrome presentations during the COVID-19 pandemic in upstate New York. | Different objective |
| 73 | Uimonen M et. al | 2022 | Emergency department visits due to coronary artery disease during COVID-19 in Finland: A register-based study. | Different objective |
| 74 | Helal A et. al | 2021 | Global effect of COVID-19 pandemic on the rate of acute coronary syndrome admissions: a comprehensive review of published literature. | Review |
| 75 | Hauguel-Moreau M et. al | 2021 | Impact of Coronavirus Disease 2019 outbreak on acute coronary syndrome admissions: four weeks to reverse the trend. | Different objective |
| 76 | Fileti L et. al | 2020 | Impact of the COVID-19 pandemic on coronary invasive procedures at two Italian high-volume referral centers. | Different objective |
| 77 | Alhejily, W et. al | 2021 | Impact of the COVID-19 Pandemic on Patients With Acute Coronary syndrome: A Tertiary Center Experience With Primary Percutaneous Intervention and Early Invasive Strategy | Different objective |
| 78 | Østergaard L et. al | 2021 | Incidence of acute coronary syndrome during national lock-down: Insights from nationwide data during the Coronavirus disease 2019 (COVID-19) pandemic. | Different objective |
| 79 | Alasnag M et. al | 2021 | Management of Acute Coronary Syndrome During the MERS-CoV Outbreak - Single-Center Experience. | Different objective |
| 80 | Mueller, KAL et. al | 2021 | Numbers and phenotype of non-classical CD14(dim)CD16(+) monocytes are predictors of adverse clinical outcome in patients with coronary artery disease and severe SARS-CoV-2 infection | Different objective |
| 81 | Vacanti G et. al | 2020 | Reduced rate of admissions for acute coronary syndromes during the COVID-19 pandemic: an observational analysis from aÂ tertiary hospital in Germany. | Different objective |
| 82 | Flori M et. al | 2021 | Reduction in acute coronary syndromes during coronavirus disease 2019 global pandemic: data from the Marche region of Italy. | Different objective |
| 83 | Kapelios CJ et. al | 2021 | The effect of the COVID-19 pandemic on acute coronary syndrome hospitalizations and out-of-hospital cardiac arrest in Greece. | Different objective |
| 84 | Chan, DZL et. al | 2020 | The impact of a national COVID-19 lockdown on acute coronary syndrome hospitalisations in New Zealand (ANZACS-QI 55) | Different objective |
| 85 | Ruparelia N et. al | 2020 | The missing acute coronary syndromes in the COVID-19 era. | Different objective |
| 86 | Kazirod-Wolski, K et. al | 2021 | The Most Relevant Factors Affecting the Perioperative Death Rate in Patients with Acute Coronary Syndrome and COVID-19, Based on Annual Follow-Up in the ORPKI Registry | Different objective |
| 87 | Skoda, R et. al | 2021 | The secondary effect of the first wave of COVID-19 and its consequences on myocardial infarction care in a high volume Hungarian cardiovascular centre | Different objective |
| 88 | Kurt, E et. al | 2021 | The Usefulness of Shock Index and Modified Shock Index in Predicting the Outcome of COVID-19 Patients | Different objective |
| 89 | Kite TA et. al | 2021 | International Prospective Registry of Acute Coronary Syndromes in Patients With COVID-19. | Different objective |
| 90 | Tendulkar, P et. al | 2022 | Descriptive Epidemiology of COVID-19 Deaths during the First Wave of Pandemic in India: A Single-center Experience | Data of interest unavailable |
| 91 | Nekaeva et. al | 2022 | Gender Characteristics of the Novel Coronavirus Infection (COVID-19) in Middle-Aged Adults. | Data of interest unavailable |
| 92 | Gore et. al et. al | 2022 | Coronavirus Disease 2019 and Hospital Readmissions: Patient Characteristics and Socioeconomic Factors Associated With Readmissions in an Urban Safety-Net Hospital System. | Full text unavailable |
| 93 | Job et. al | 2022 | Renin-Angiotensin System Blocker in COVID-19. A Single Center Study. | Full text unavailable |
| 94 | Zdravkovic et. al | 2021 | Development and Validation of a Multivariable Predictive Model for Mortality of COVID-19 Patients Demanding High Oxygen Flow at Admission to ICU: AIDA Score. | Different outcome |
| 95 | Tiryaki et. al | 2022 | INVESTIGATION OF THE RELATIONSHIP BETWEEN THE CLINICAL COURSE OF THE ELDERLY PATIENTS DIAGNOSED WITH COVID-19 AND PNEUMOCOCCAL IMMUNIZATION | Full text unavailable |
| 96 | Puttegowda et. al | 2021 | Patterns of cardiovascular diseases in COVID-19 patients admitted to tertiary cardiac care centre. | Data of interest unavailable |
| 97 | Jackson et. al | 2020 | Predictors at Admission of Mechanical Ventilation and Death in an Observational Cohort of Adults Hospitalized With Coronavirus Disease 2019. | Data of interest unavailable |
| 98 | Gujski et. al | 2022 | The Prevalence of Acute Respiratory Distress Syndrome (ARDS) and Outcomes in Hospitalized Patients with COVID-19-A Study Based on Data from the Polish National Hospital Register. | Data of interest unavailable |
| 99 | Giannis et. al | 2021 | Incidence of Venous Thromboembolism and Mortality in Patients with Initial Presentation of COVID-19. | Data of interest unavailable |
| 100 | Jain et. al | 2020 | A Retrospective Observational Study to Determine the Early Predictors of In-hospital Mortality at Admission with COVID-19 | Data of interest unavailable |
| 101 | Davoudi-Monfared et. al | 2020 | A Randomized Clinical Trial of the Efficacy and Safety of Interferon β-1a in Treatment of Severe COVID-19 | Data of interest unavailable |
| 102 | Luykx et. al | 2021 | Are psychiatric disorders risk factors for COVID-19 susceptibility and severity? a two-sample, bidirectional, univariable, and multivariable Mendelian Randomization study | Data of interest unavailable |
| 103 | Øverstad | 2020 | Seventy patients treated for COVID-19 by √òstfold Hospital Trust. | Full text not available in English |
