# Supplementary material for: A systematic review and meta-analysis on the prevalence and impact of coronary artery disease in hospitalized COVID-19 patients
Source: Heliyon. 2023 Aug 25;9(9):e19493. doi: 10.1016/j.heliyon.2023.e19493 (PMC10480662; doi:10.1016/j.heliyon.2023.e19493)
Supplement: Multimedia component 3 [file mmc3.docx]

**A Systematic Review and Meta-Analysis on the Prevalence and Impact of Coronary Artery Disease in Hospitalized COVID-19 Patients**

**Contents:**

[**Supplementary Figure 1:** Risk of Bias Assessment 2](#_Toc115171993)

[**Supplementary Figure 2:** Sensitivity analysis (leave-one-out estimates) of prevalence of Coronary Artery Diseases (CAD) among hospitalized COVID19 patients 2](#_Toc115171994)

[**Supplementary** **Figure 3:** Forest plot estimating pooled prevalence of Coronary Artery Diseases (CAD) among COVID -19 hospitalized patients after removing the influential and the high risk studies 3](#_Toc115171995)

[**Supplementary Figure 4:** Forest plot of the sensitivity analysis for the association between Coronary Artery Diseases (CAD) and mortality among hospitalized COVID-19 4](#_Toc115171996)

[**Supplementary Figure 5:** Forest plot of the association between Coronary Artery Diseases (CAD) and mortality among hospitalized COVID-19 after removing high risk of bias study 4](#_Toc115171997)

[**Supplementary Figure 6:** Forest plot for the sub-group analysis of the prevalence rate of Coronary Artery Diseases (CAD) among hospitalized COVID-19 5](#_Toc115171998)

[**Supplementary Figure 7:** Forest plot for the sub-group analysis of the association estimate between Coronary Artery Diseases (CAD) and mortality among hospitalized COVID-19 6](#_Toc115171999)

**Supplementary Figure 1:** Risk of Bias Assessment

Using Newcastle-Ottawa quality assessment scale (NOS)(1), we identified three studies(2) (3) (4) out of seventy-six to have a high risk of bias.


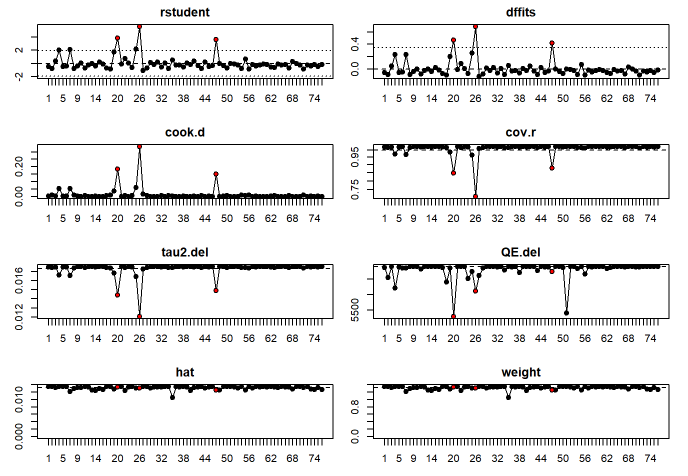


**Supplementary Figure 2:** Sensitivity analysis (leave-one-out estimates) of prevalence of Coronary Artery Diseases (CAD) among hospitalized COVID19 patients

- The influential studies were marked with red color and they were removed from further analysis. The removed studies were (Scoccia 2021(5), Salinas, 2021(2), Mousseaux, 2021(6)).


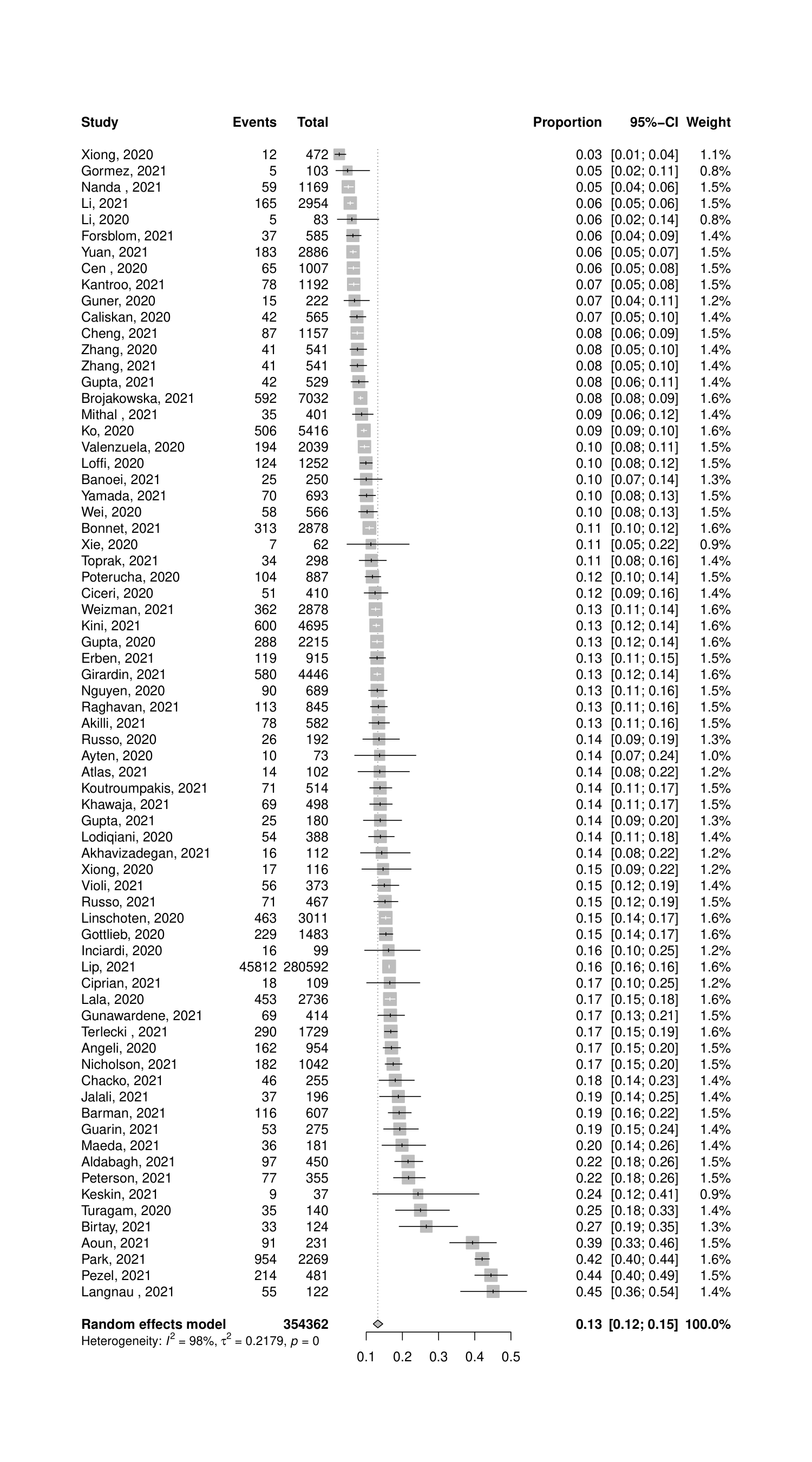


**Supplementary** **Figure 3:** Forest plot estimating pooled prevalence of Coronary Artery Disease (CAD) among COVID -19 hospitalized patients after removing the influential and the high risk studies


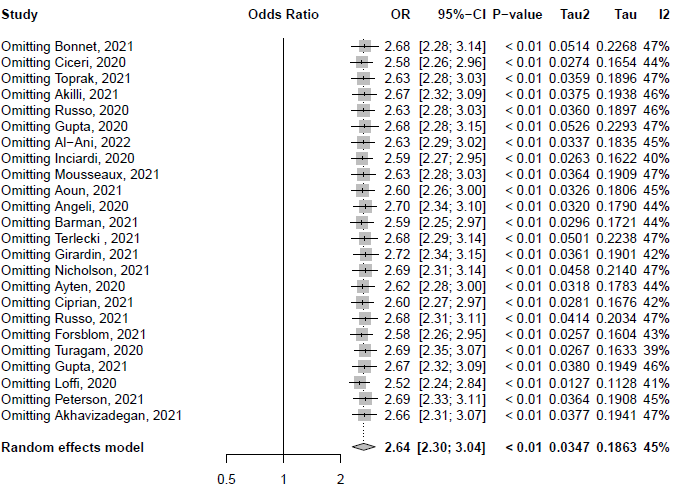


**Supplementary Figure 4:** Forest plot of the sensitivity analysis for the association between Coronary Artery Disease (CAD) and mortality among hospitalized COVID-19


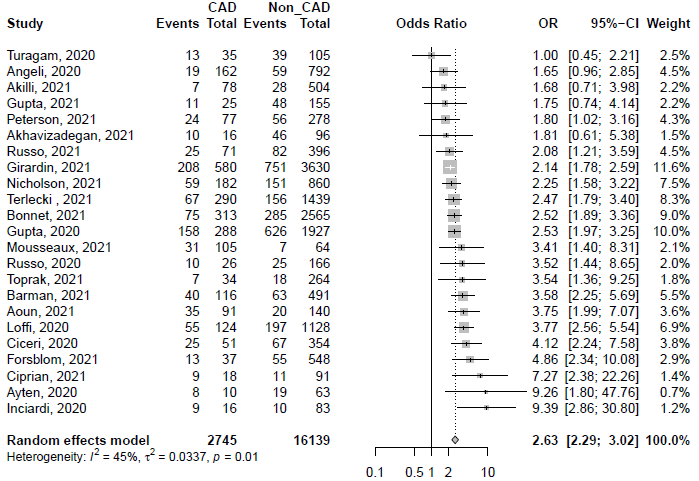


**Supplementary Figure 5:** Forest plot of the association between Coronary Artery Disease (CAD) and mortality among hospitalized COVID-19 after removing high risk of bias study

- The removed study was Al-Ani, 2022(4)


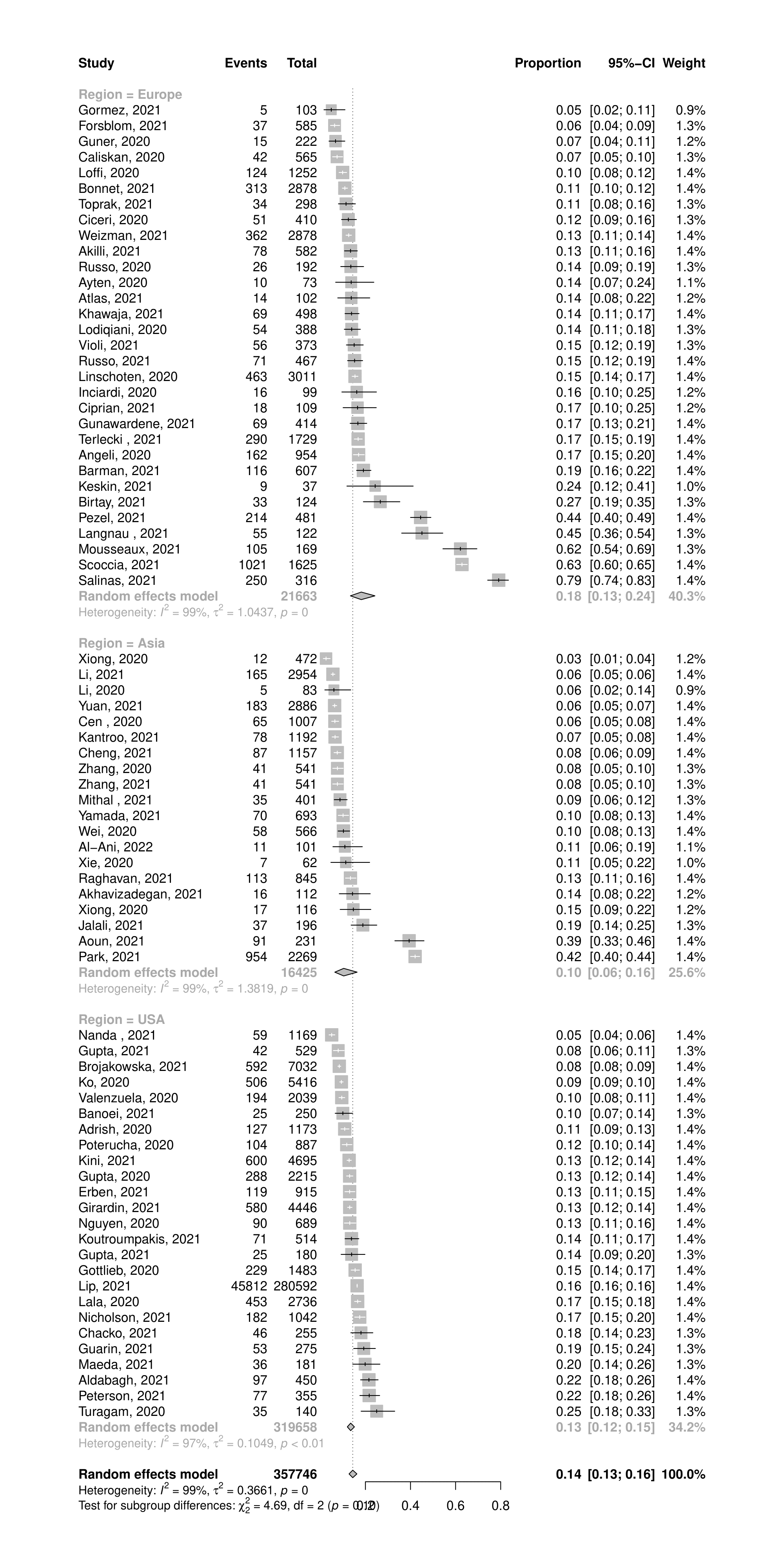


**Supplementary Figure 6:** Forest plot for the sub-group analysis of the prevalence rate of Coronary Artery Disease (CAD) among hospitalized COVID-19


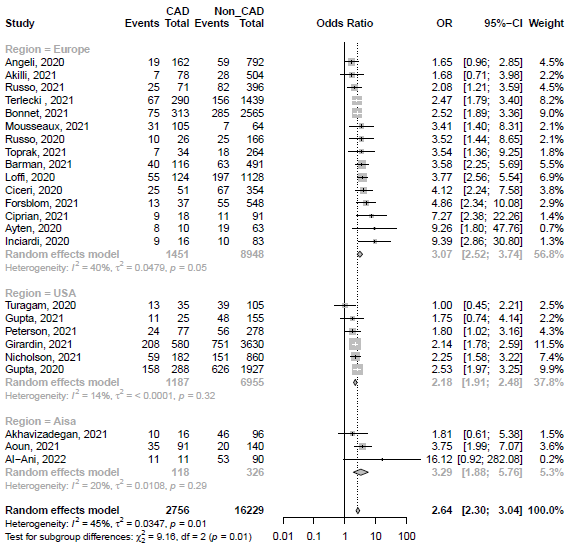


**Supplementary Figure 7:** Forest plot for the sub-group analysis of the association estimate between Coronary Artery Disease (CAD) and mortality among hospitalized COVID-19

**References:**

1. Wells G, Shea B, O’Connell D, Peterson J, Welch V, Losos M, et al. The Newcastle–Ottawa Scale (NOQAS) for Assessing the Quality of Non-Randomized Studies in Meta-Analysis. Ottawa Hosp. 2004;

2. Salinas P, Travieso A, Vergara-Uzcategui C, Tirado-Conte G, Macaya F, Mejía-Rentería H, et al. Clinical profile and 30-day mortality of invasively managed patients with suspected acute coronary syndrome during the COVID-19 outbreak. Int Heart J. 2021;62(2):274–81.

3. Adrish M, Chilimuri S, Mantri N, Sun H, Zahid M, Gongati S, et al. Association of smoking status with outcomes in hospitalised patients with COVID-19. BMJ Open Respir Res. 2020;7(1):1–6.

4. Al-Ani A, Ghazzay HI, Al Shawi AF, Al-Koubaisy HNE, Al-Ani F, Aldouri M. Association of chronic diseases with mortality among hospitalized patients with COVID-19 treated with convalescent plasma: Evidence from a single center - Iraq. J Emerg Med Trauma Acute Care. 2022;2022(2).

5. Scoccia A, Gallone G, Cereda A, Palmisano A, Vignale D, Leone R, et al. Impact of clinical and subclinical coronary artery disease as assessed by coronary artery calcium in COVID-19. Atherosclerosis. 2021;328(Jualy 2021):136–43.

6. Mousseaux E, Fayol A, Danchin N, Soulat G. Association between coronary artery calcifications and 6-month mortality in hospitalized patients with COVID-19. Diagn Interv Imaging. 2021;102(12):717−725 Original.
